# Community health workers and health equity in low- and middle-income countries: systematic review and recommendations for policy and practice

**DOI:** 10.1186/s12939-021-01615-y

**Published:** 2022-04-11

**Authors:** Sonia Ahmed, Liana E. Chase, Janelle Wagnild, Nasima Akhter, Scarlett Sturridge, Andrew Clarke, Pari Chowdhary, Diana Mukami, Adetayo Kasim, Kate Hampshire

**Affiliations:** 1 Department of Anthropology, Durham University, South Road, Durham, DH1 3LE UK; 2 Durham Research Methods Centre, Durham University, South Road, Durham, DH1 3LE UK; 3 Save the Children UK, 1 St John’s Ln, Farringdon, London, EC1M 4AR UK; 4 CARE USA, 151 Ellis St NE, Atlanta, GA 30303 USA; 5 Amref Health Africa, Langata Rd, Nairobi, Kenya

**Keywords:** Health equity, Community health workers, Low- and middle-income countries, Global health

## Abstract

**Background:**

The deployment of Community Health Workers (CHWs) is widely promoted as a strategy for reducing health inequities in low- and middle-income countries (LMIC). Yet there is limited evidence on whether and how CHW programmes achieve this. This systematic review aimed to synthesise research findings on the following questions: (1) How effective are CHW interventions at reaching the most disadvantaged groups in LMIC contexts? and (2) What evidence exists on whether and how these programmes reduce health inequities in the populations they serve?

**Methods:**

We searched six academic databases for recent (2014–2020) studies reporting on CHW programme access, utilisation, quality, and effects on health outcomes/behaviours in relation to potential stratifiers of health opportunities and outcomes (e.g., gender, socioeconomic status, place of residence). Quantitative data were extracted, tabulated, and subjected to meta-analysis where appropriate. Qualitative findings were synthesised using thematic analysis.

**Results:**

One hundred sixty-seven studies met the search criteria, reporting on CHW interventions in 33 LMIC. Quantitative synthesis showed that CHW programmes successfully reach many (although not all) marginalized groups, but that health inequalities often persist in the populations they serve. Qualitative findings suggest that disadvantaged groups experienced barriers to taking up CHW health advice and referrals and point to a range of strategies for improving the reach and impact of CHW programmes in these groups. Ensuring fair working conditions for CHWs and expanding opportunities for advocacy were also revealed as being important for bridging health equity gaps.

**Conclusion:**

In order to optimise the equity impacts of CHW programmes, we need to move beyond seeing CHWs as a temporary sticking plaster, and instead build meaningful partnerships between CHWs, communities and policy-makers to confront and address the underlying structures of inequity.

**Trial registration:**

PROSPERO registration number CRD42020177333.

**Supplementary Information:**

The online version contains supplementary material available at 10.1186/s12939-021-01615-y.

## Background

The deployment of Community Health Workers (CHWs) has been advocated by the World Health Organisation (WHO) as a key strategy for reaching the most marginalised populations to achieve Universal Health Coverage and reduce health inequities, especially in Low- and Middle-Income Countries (LMIC) [1, 2]. Because of their geographic and cultural proximity to the populations they serve, CHWs often are described as vital bridges between health services and communities, uniquely positioned to extend care to poor, hard-to-access and underserved groups that fall beyond the reach of institution-based services [1, 3].

However, despite the strong equity justification for CHW programmes in policy discourse, important gaps remain in our understanding of how and to what extent CHWs contribute to reducing disparities in healthcare access and outcomes. In a systematic review of reviews, conducted to inform the latest WHO guideline on optimizing CHW programmes [2, 4, 5], only three of the 122 reviews identified considered equity as an outcome in LMIC [6–8]; of these, two were limited to specific health issues: mental healthcare and maternal & newborn health respectively [7, 8]. Barnett and colleagues’ review [8] (papers published 1990–2015) found evidence that incorporation of CHWs can reduce mental healthcare disparities experienced by underserved populations. Blanchard and colleagues’ [7] review (papers published 1996–2017) concluded that CHW programmes may contribute to reducing socioeconomic inequities in maternal and newborn health, but it also highlighted a need for further research that looks beyond equitable coverage to examine equity of effects on health outcomes and behaviours. McCollum and colleagues [6], whose review covered a more comprehensive set of CHW activities (papers published 2004–2014), found evidence of equitability in accessibility and utilisation of CHW services for underserved groups, but did not examine the health impacts in these groups. The resulting WHO guideline identifies equity implications of CHW policies as an important area for future research; it also calls for the development of conceptual models of the roles CHWs play in community mobilization for health [2].

The 2015 adoption of the SDGs has generated renewed interest in health inequities and a ‘rapid growth of evidence’ on the role of CHW programmes in addressing these ([4], p. 2). Given that the bulk of studies considered by previous reviews were published pre-2015, there is a pressing need to take stock of what we know now about the impact of such programmes on health equity in LMIC. The present systematic review constitutes an update of McCollum et al.’s [6] comprehensive review, beginning where they left off (April 2014), to synthesise recent research findings on the equitability of CHW interventions in LMIC. Specifically, it seeks to address two important questions:

1. How effective are CHW interventions at *reaching* the most disadvantaged groups in LMIC contexts?
2. What evidence exists on whether and how these programmes *reduce health inequities* in the populations they serve?

### Definitions

We adopt the WHO’s definition of CHWs as health workers who have received some training (up to 2 years) but are not considered health professionals, and who are based in communities, meaning they provide services outside of health facilities or at peripheral facilities not staffed by health professionals [2, 3]. *Health inequities* are defined as unfair and avoidable differences in health between groups, including those based on place of residence, social identity, socioeconomic status, gender, and disability, while *health equity* is defined as the absence of such differences [9, 10]. Given that health inequity is a ‘normative concept, and thus cannot be precisely measured’, we followed WHO guidance on using measurable differences between subgroups within a population (or *health inequalities*) to gain insight into health inequity ([9], p. 6). Following O’Neill and colleagues, we use the term ‘equity stratifiers’ to refer to ‘socially stratifying factors that drive variations in health outcomes’ ([10], p. 58), taking as our starting point those listed in the PROGRESS framework: **P**lace of residence, **R**ace/ethnicity/culture/language, **O**ccupation, **G**ender/sex, **R**eligion, **E**ducation, **S**ocioeconomic status, and **S**ocial capital [10, 11]; based on our previous work, we additionally included caste and disability. For analytical purposes, we define a ‘pro-equity’ programme as one that reduces existing health inequities by reaching and/or benefitting disadvantaged groups the most. By contrast, ‘anti-equity’ programmes disproportionately reach/benefit already-advantaged groups, while ‘equity-neutral’ programmes reach/benefit both advantaged and disadvantaged groups equally. Notably, ‘equity neutral’ and even ‘anti-equity’ initiatives may improve healthcare overall, but they do not reduce pre-existing health inequities.

## Methods

### Search strategy

In order to maximise efficiency and avoid duplication, this systematic review was designed as an update of McCollum and colleagues’ prior review, for which searches were carried out in April 2014 ( [7]; see Additional file 1 for detailed search strategy). Following McCollum et al.’s search strategy, three sets of search terms were used:

- “community health worker terms” (including all the different names used for various categories of CHW in LMIC), **AND**
- “equity terms”: terms representing known equity stratifiers based on the PROGRESS Framework [10, 11] and used by used by McCollum et al. [6] plus additional terms “caste” and “ethnicity” **AND**
- “outcomes terms”, including those associated with programme delivery (coverage, reach, access, uptake and acceptability of care) plus health behaviours and outcomes.

Searches were conducted in spring 2020 in six scholarly databases (Pubmed, SCOPUS, Cochrane Central Register of Controlled Trials, Web of Science (Social Science Citation Index), CINAHL, and Anthrosource), with delimiters of English language and publication date of 2014-present.

### Selection criteria

Eligibility criteria were guided by recommendations for equity-related reviews and tailored to capture the widest possible evidence base to inform policy and practice [11, 12]. We included a range of *study designs* including qualitative, quantitative (controlled and non-controlled), and mixed-methods. Relevant systematic reviews were included for reference screening only. With regard to *publication type*, we included peer-reviewed journal articles published in English between 2014 and 2020, which reported findings of primary research carried out on CHW interventions in LMIC. A CHW *intervention* was defined as any intervention that aims to improve health and is delivered in primary or community settings by CHWs meeting the above-mentioned definition; no restrictions were imposed on *patients/populations* served. Eligible studies reported on differences by equity stratifier in *service delivery* (including coverage, accessibility, acceptability, utilisation, and quality of CHW-delivered services), CHW-promoted *outcomes* (health indicators and behaviours), or both.

We excluded studies on interventions that did not entail the deployment or involvement of CHWs meeting the WHO definition set out above [2] (for example, interventions delivered by self-defined health professionals or trainee health professionals, those provided as part of other professional roles (e.g. by teachers), patient support groups, self-help interventions, training provided to family members to care for an ill member, peer support and peer counselling programmes, and short-term one-off projects such as those which train volunteers for a single vaccination campaign).

### Screening

All database search results were imported into Covidence software for screening, automatically removing duplicates in the process. Two authors independently screened titles and abstracts to assess potential relevance, with 20% overlap to ensure consistency. A threshold of 90% agreement before arbitration was required for authors to screen the remaining abstracts independently. Discussion was used to resolve discrepancies between the reviewers [13]. Full-text copies of articles were then obtained and the first 10% assessed against inclusion criteria by two reviewers to identify any discrepancies. Any disagreements were resolved through discussion and when necessary by seeking a third author’s opinion. The reviewers then completed screening independently. The reference lists of included studies were searched for further relevant publications.

### Quality appraisal

Quality and risk of bias were assessed using different tools depending on study design. Risk of bias in randomised controlled trials was assessed using the Revised Cochrane Risk of Bias Tool for Randomized Trials [14]. The quality of non-randomised studies that were not cross-sectional (e.g., quasi-experimental studies) was assessed using the NIH Quality Assessment Tool for Before-After (Pre-Post) Studies with No Control Group. Quality assessment of cross-sectional studies was done using the NIH Quality Assessment Tool for Observational Cohort and Cross-sectional Studies. Funnel plots were produced to assess reporting/publication bias (see Additional file 5).

Qualitative studies were appraised using a method whereby experienced qualitative researchers ranked studies as ‘key’ (methodologically and conceptually strong as well as highly relevant to review questions), ‘satisfactory’ (methodologically and conceptually acceptable or strong, some relevance to research questions), or ‘thin’ (methodologically or conceptually flawed and/or containing little relevant data) (modified from [15, 16]).^1^ Appraisal was conducted with 10% overlap to ensure consistency. In the case of mixed-methods studies, qualitative and quantitative methods were appraised separately.

In line with guidance on equity-related reviews (which emphasizes inclusion based on ‘fitness for purpose’ rather than a standard hierarchy of evidence [11, 12]) and given that most eligible studies were deemed of satisfactory or good quality (see Results), we did not exclude any studies from the present narrative synthesis on the basis of quality.

### Data synthesis

Different methods of preliminary synthesis were adopted for quantitative and qualitative findings following guidance on narrative synthesis [17]. Quantitative data were first extracted and tabulated using a structured Excel template. Textual descriptions of statistically significant findings were then produced and grouped by pre-specified equity stratifiers using the modified PROGRESS framework (see above) to generate a narrative synthesis. Meta-analysis of pooled data from the outcomes and equity stratifiers was performed using the R software package where data from at least three studies were available. Random effects models were used for all meta-analyses, accounting for heterogeneity (*τ*^2^) between trials [18]. Based on the extracted metrics (*β*_*k*_) from study *k*, the pooled effect was calculated as:

$$Pooled\ \beta =\frac{\sum_{k=1}^K{W}_k{\beta}_k}{\sum_{k=1}^K{W}_k}$$

where *W*_*k*_ = (*SE*_*k*_^2^ + *τ* ^2^)^−1^ is the weight for the individual study based on variability within and between studies [19, 20]. Note that *β*_*k*_ for categorical data denoted log-odds ratio and *τ*^2^ is estimated from the reported standard errors (*SE*_*k*_). Forest plots were used to present the results with Cochrane’s-Q test, I^2^ statistics and visual dispersion of individual results to understand statistical heterogeneity.

Qualitative findings were synthesised using thematic analysis in NVivo version 12.6.0. A coding framework was developed based on an existing framework for research on health equity (PROGRESS-Plus [10],; see Additional file 2); additional codes were added for emergent themes identified inductively during analysis and subject to discussion within the wider research team. Two authors coded the first 10% of publications independently and discussed to resolve any discrepancies. These two authors then divided the remaining papers for independent coding. Finally, qualitative and quantitative findings on each equity stratifier were grouped and triangulated to generate the final narrative synthesis.

## Results

**Fig. 1:**
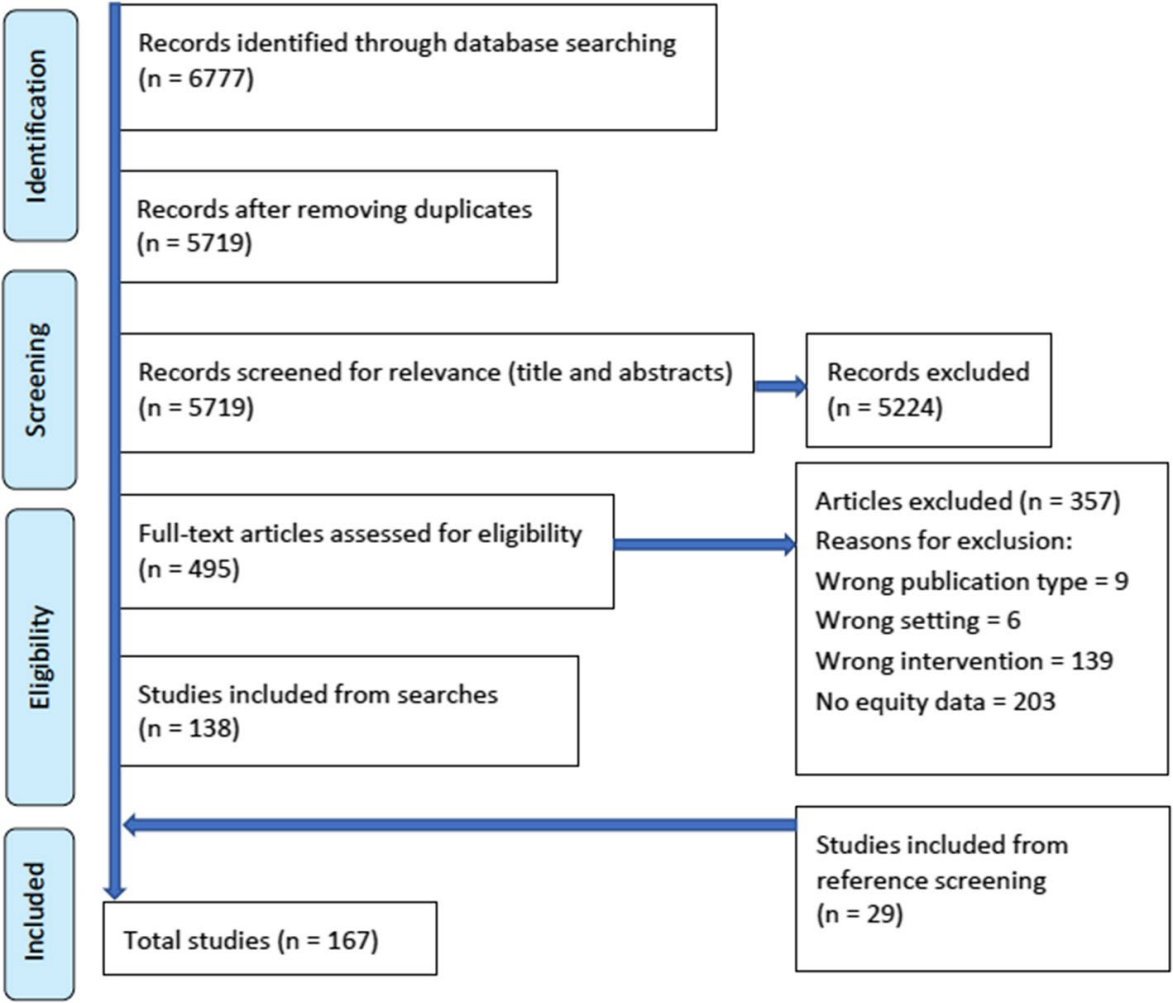
PRISMA flow diagram

**Fig. 2:**
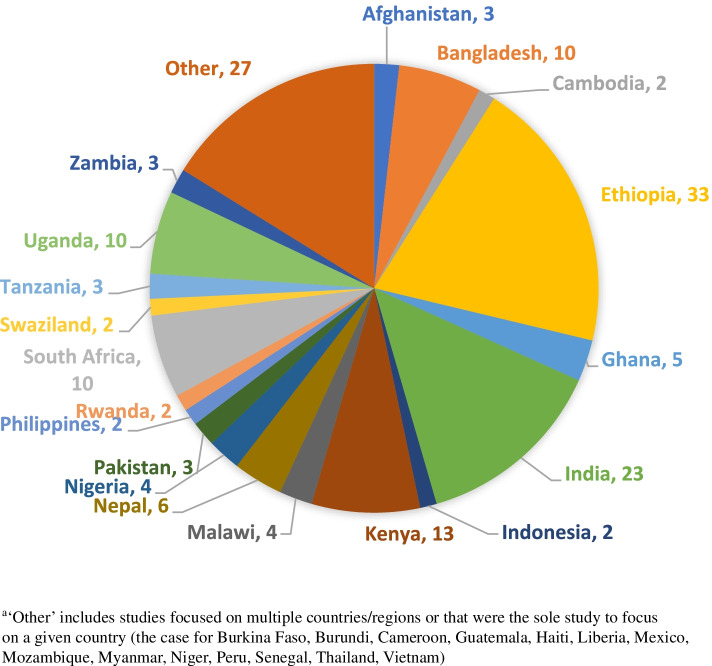
Countries of eligible studies^a^

Altogether, 167 studies were identified that met the eligibility criteria (see Fig. 1). These were carried out in 33 LMIC, with Ethiopia (20%) and India (14%) being particularly strongly represented (see Fig. 2). Of the 167 studies, 87 were quantitative or mixed-method, adopting a range of study designs (predominantly cross-sectional, quasi-experimental and RCTs), 72 used qualitative methods, and eight were systematic reviews (included for reference screening; see Table 1). There was a high level of heterogeneity in types of interventions and outcomes reported on. Of the 87 studies reporting quantitative findings, 66 were found to be of good quality, 11 of moderate quality, 8 of poor quality and 2 could not be assessed. However, only 8 studies met the ‘gold standard’ of high-quality randomised controlled trials (RCTs) with strong study design and low risk of bias. Of the 82 papers reporting qualitative findings, 18 were assessed as being ‘key’, 40 were ‘satisfactory’ and 24 were ‘thin’. Overlap with other key systematic reviews noted above was as follows: no overlap with McCollum et al. [6] or Barnett et al. [8]; nine papers in common with Blanchard et al. [7]. Methodological characteristics and risk of bias for all studies are reported in Additional files 3 and 4. Owing to the heterogeneity of study designs and variables, it was only possible to conduct meta-analyses on a very limited number of relationships: facility delivery by distance from facility; and breastfeeding and use of maternity services by maternal SES and maternal education.

**Table 1:** Methodology of eligible studies

Study type	Number
Quantitative or mixed-methods	
Randomised Control Trial (RCT)	14 (1)^a^
Cross-sectional	49 (6)
Pre-Post or quasi-experimental	20 (1)
Observational Cohort	3
Case Study	1 (1)
Qualitative	72
Systematic Review	7
Combined	
Systemic Review + Qualitative	1
Total	167

^a^Numbers in brackets indicate mixed-methods studies

Review findings are presented as follows. In sections “Overview of quantitative findings” and “Synthesis of qualitative and quantitative findings by equity stratifier”, we summarize available data on whether and how CHWs reach and improve health in different marginalized groups relative to more advantaged groups within the populations they serve. The first summarises general trends evident in quantitative findings on CHW service delivery and outcomes across all stratifiers. The next section then provides a more in-depth narrative synthesis of qualitative and quantitative findings pertaining to each equity stratifier, including a synthesis of programme elements common to pro-equity outcomes. The final section identifies some additional pathways (beyond straightforward service delivery) through which CHW programmes can contribute to health equity.

### Overview of quantitative findings

**Fig. 3:**
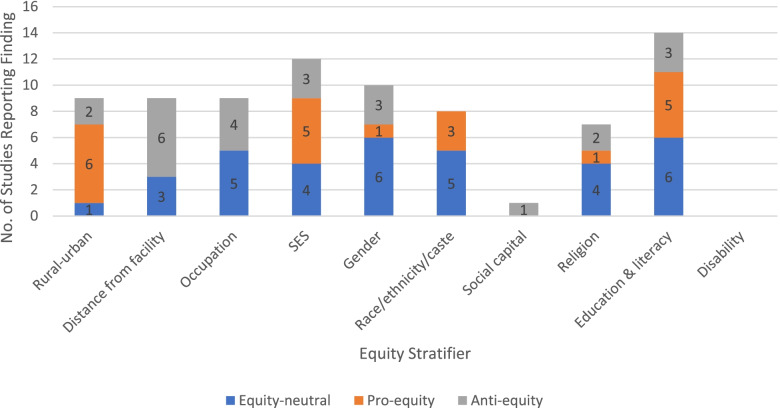
Quantitative findings on CHW service delivery

**Fig. 4:**
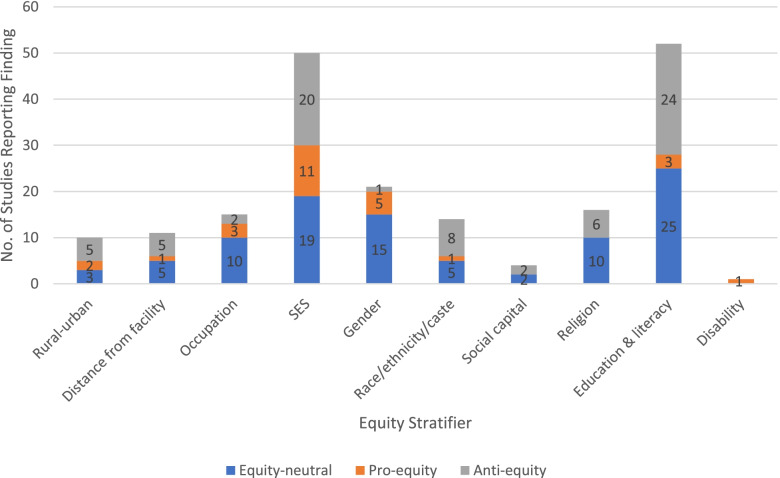
Quantitative findings on CHW-promoted outcomes

In line with the definitions presented above, we characterized quantitative findings on CHW service delivery (reach, uptake, etc.) and CHW-promoted outcomes (health, behavioural) as ‘pro-equity’ (better reach or outcomes in disadvantaged groups), ‘equity-neutral’ (no significant differences between groups in reach or outcomes), ‘mixed’ (different findings for the same outcome depending on dimension of equity stratifier examined), and ‘anti-equity’ (lower reach or poorer outcomes in disadvantaged groups).^2^ Details of findings by outcome and equity stratifier for each eligible study are included in Additional file 4. Figures 3 and 4 provide an overview of the total number of quantitative studies reporting each type of finding by equity stratifier.

Generally speaking, across multiple stratifiers, CHW programmes appeared to achieve greater equitability in *service delivery* than in *outcomes*. There was evidence that CHW programmes successfully reach many (although not all) disadvantaged groups. Overall, of 79 reported findings on CHW service delivery, 21 (27%) were pro-equity, 24 (30%) were anti-equity, and 34 (43%) were equity-neutral (Fig. 3). Pro-equity findings outnumbered anti-equity ones across several stratifiers, including rural/urban, socioeconomic status, race/ethnicity/caste and education. However, this was not the case for distance from facility, occupation or religion, indicating that some marginalised groups are still being excluded.

Even where CHW programmes reach disadvantaged groups, however, the evidence is less clear that this translated into more equitable health outcomes. Overall, of 193 reported findings on CHW-promoted health behaviours or outcomes, just 26 (13%) were pro-equity, while 94 (49%) were equity-neutral and 73 (38%) were anti-equity (Fig. 4). For only two stratifiers (gender and occupation) did pro-equity findings outnumber anti-equity ones. Across all other stratifiers (rural/urban, distance from facility, socioeconomic status, race/ethnicity/caste, social capital, religion and education/literacy), already privileged groups continued to enjoy better health outcomes than disadvantaged ones, despite the presence of CHW programmes. Altogether, the ratio of anti-equity:pro-equity findings for CHW programme *reach* was approaching parity (8:7) while, for outcomes, it was almost 3:1.

Because the majority of quantitative studies included in the review lack a strong causal design, we attempted to conduct a sub-group analysis of the eight high-quality RCT studies in order to test the robustness of the findings. Unfortunately the number of reported findings on CHW service delivery (*N* = 3) was too small to analyse meaningfully. There were 18 reported findings on health outcomes, of which 12 were equity-neutral, four were anti-equity and two were pro-equity, indicating a broadly similar picture to the full set of studies, with little evidence of a strong pro-equity effect on health outcomes. In the next section we elaborate and contextualize trends specific to each equity stratifier, drawing on both quantitative and qualitative evidence.

### Synthesis of qualitative and quantitative findings by equity stratifier

#### Place of residence

Studies on the impacts of place of residence on the reach and outcomes of CHW programmes focused largely on two key dimensions: rural-urban differences and distance from health facilities. Evidence from quantitative data on coverage and utilisation suggests that CHW programmes have been successful in reaching rural communities (normally underserved in the wider health system). Six studies reported that rural communities were better served than urban communities across a number of different programme types [21–26], with only two studies finding better coverage in urban areas [26, 27] and one reporting no significant differences [28]. However, there was evidence that CHW programmes often fail to reach the *most remote* rural areas: those which are far even from CHW-led health facilities/posts. Six studies found higher utilisation and coverage among those living closer to such facilities [25, 29–33], while only three studies found no association between distance and CHW service coverage/uptake [25, 34, 35].

Qualitative data shed some light on these findings. CHW programmes often employed CHWs who themselves lived in rural communities, greatly facilitating access for this group; in urban settings, CHWs reported more difficulties making contact with and securing trust from people due to the lack of clear community leadership, busy lifestyles, a tendency to work outside the home, and the wide availability of private health professionals [36, 37]. However, disparities in access to CHWs reportedly often persisted within rural areas; large catchment areas in sparsely populated regions often meant that the majority of villages or settlements could not have a resident CHW, and health facilities in the nearest town were sometimes more accessible than CHWs based in another village [38].

A number of strategies were discussed for overcoming place-related barriers to equitable CHW service delivery. Providing CHWs bicycles and motorcycles increased their ability to reach rural and remote populations, although some communities deemed it inappropriate for women CHWs to use these modes of transportation. Hiring male CHWs was described as a strategy for promoting access in some rural and remote communities as men were able to travel more quickly and freely [39, 40]. Mobile phones were frequently used to overcome place-related access difficulties; however, connectivity could be problematic in the most remote areas and the costs of phone ownership, credit, and even battery charging could be prohibitive for the poorest residents as well as for CHWs themselves [36, 41–44].

**Fig. 5:**
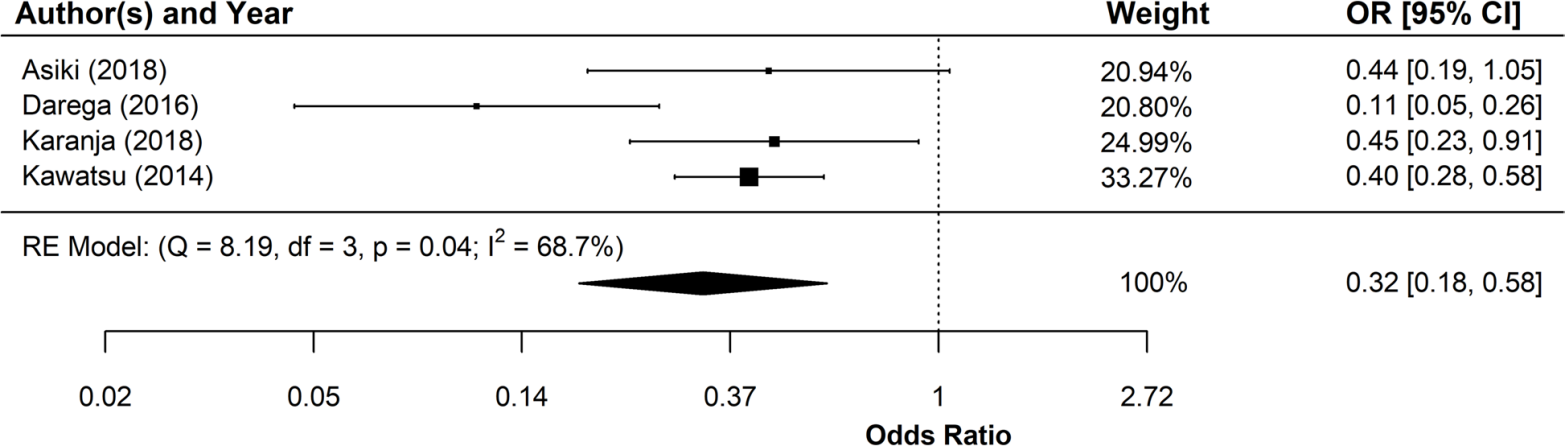
Meta-analysis of the association between facility delivery and distance between place of residence and health facility

A second subset of articles reported on differences in CHW-promoted health behaviours and outcomes by place of residence. These findings suggested that equitable service delivery does not necessarily translate into improved outcomes in disadvantaged groups. Five studies investigating rural/urban differences in CHW programme outcomes reported poorer outcomes for rural compared with urban populations [27, 45–48]. Three studies found no significant rural/urban differences [28, 48, 49], and only two studies reported better outcomes for rural residents [48, 50]. Similarly, five studies on the effects of distance found that, following CHW MNCH interventions, those living further from health facilities had poorer outcomes compared with those living closer by [51–55]. By contrast, only one study reported a pro-equity effect [56]. The other five reported no significant differences across groups [33, 57–60]. Meta-analysis confirmed that mothers living further from a health facility were less likely to give birth in the facility than those who lived closer despite CHW promotion of facility birth (pooled effect: 0.32, confidence interval (CI): 0.18, 0.58) (see Fig. 5).

Qualitative findings point to some possible reasons place-based differences in health outcomes and behaviours might persist in communities where CHWs work to address these. Most importantly, CHWs reported difficulties convincing clients in rural, remote areas to engage in treatments and behaviours that required travelling to health facilities. Transportation between rural and remote communities and health facilities where CHWs referred clients was often unreliable and costly, particularly when arranged on short notice or at night [44, 61–65]. Transportation-related difficulties were also exacerbated in sites of active conflict; during the rainy season; and when clients were children, had a disability, were pregnant or were gravely ill [65–67]. For example, a CHW in India described the challenges she faced convincing pregnant women to visit health facilities for antenatal care (ANC) services: ‘Our village is about 5 hours walking distance from the road [nearest functional PHC is 85 KM]; with no proper transportation pregnant mothers find it difficult to go for ANC check-up’ ([65], p. 9).

Place-related barriers appeared to interact with health system weaknesses to multiply disadvantage: when clients had to make long and costly journeys to get care, a single negative experience (e.g. absent health professional) could dissuade them from seeking care again in the future and even undermine their trust in CHWs [67]. Some questioned the value of CHW initiatives to increase demand for professional services in places where supply remained inadequate, arguing that encouraging clients to make long, arduous commutes for low-quality services could create risks to health that outweighed potential benefits [68]. At the same time, CHWs often lacked the training to address serious problems themselves, although, concerningly, some resorted to delivering curative services anyway when they knew clients had no way of reaching facilities [62, 69].

Low-cost emergency transportation services or ambulances that CHWs were empowered to call upon were found helpful in addressing some of these challenges; however these could be hampered in reaching the most marginalised by poor road conditions, long distances, and lack of mobile network coverage [70–73]. For example, a CHW in Ethiopia recounted the challenges of serving an Afari community in the desert:

Once I assisted a woman whose labour lasted more than 1 day to get to the hospital. I walked 20 kms to get a phone signal to call the ambulance but when that did not work I went home and we carried the woman for 6 or 7 h on a stretcher to the road. Then we called the…ambulance to come the last 40 kms. ([44], p. 154).

Financial incentives or transport stipends may further encourage more clients from remote rural communities to access health facilities, provided these are sufficient to cover actual transportation costs [74, 75]. Providing CHWs with a medical kit containing essential supplies was another effective strategy for reducing the need for lengthy visits to health facilities [65]. Community members in several very remote communities called for training their local CHWs to provide more curative services, given community members’ limited ability to take up facility referrals [62, 69].

Finally, a few studies examined other types of place-based disadvantage. Angeles et al. [76] reported that CHW programme activities appeared to have narrowed some disparities in health behaviours between slum and non-slum residents but did not significantly reduce disparities in the primary health outcome (childhood stunting). Qualitative findings furthermore pointed to difficulties migrant, mobile, and homeless populations faced in accessing CHW services [63, 77]; strategies for reaching these populations included the deployment of mobile CHW teams [63, 78] and improved transfer-of-care mechanisms [64].

#### Socioeconomic status

Socioeconomic status (SES) refers to a combination of social and economic factors that determine one’s class or standing within society, including education, income, employment, and social support. This section reviews studies reporting directly on SES (when this was used as a composite measure) or on financial and material indicators of SES (e.g. wealth quintile/quartile, income, home ownership/quality).^3^ Quantitative findings on SES were mixed, but suggest many CHW programmes are successful in reaching low-SES groups. Five studies reported greater coverage and/or utilisation proportionately for lower-income groups [22, 58, 79–81] and four reported no significant differences in coverage or utilisation by SES [21, 25, 31, 82]. However, three studies found that CHW programmes reached wealthier groups to a greater extent [83–85].

Qualitative analysis revealed that most CHW programmes provided services free of cost, greatly facilitating equitable coverage of all SES groups. However, rumours that CHWs would demand bribes or payment could nonetheless hinder utilisation [67]. When CHWs did charge user fees (e.g. in entrepreneurial models), this was found to incentivize CHWs to pursue a wealthier clientele [38]. Strategies for promoting equitable utilisation of CHW programmes included providing food parcels as part of CHW services; this practice made clients feel their economic struggles were recognized and strengthened trust in CHWs [64, 86]. Trust arrangements that allowed clients to reimburse CHWs when they were able were also a facilitating factor in some rural settings [36]. Offering financial incentives to identify and serve ‘indigents’ seemed to improve coverage in this group; however, CHWs reported problems distinguishing who qualified for this status and resulting feelings of jealousy and injustice in the community [77]. Offering incentives to serve the most vulnerable may also end up privileging those with highly visible vulnerabilities, such as the physically handicapped, over other marginalised groups (ibid.).

**Fig. 6:**
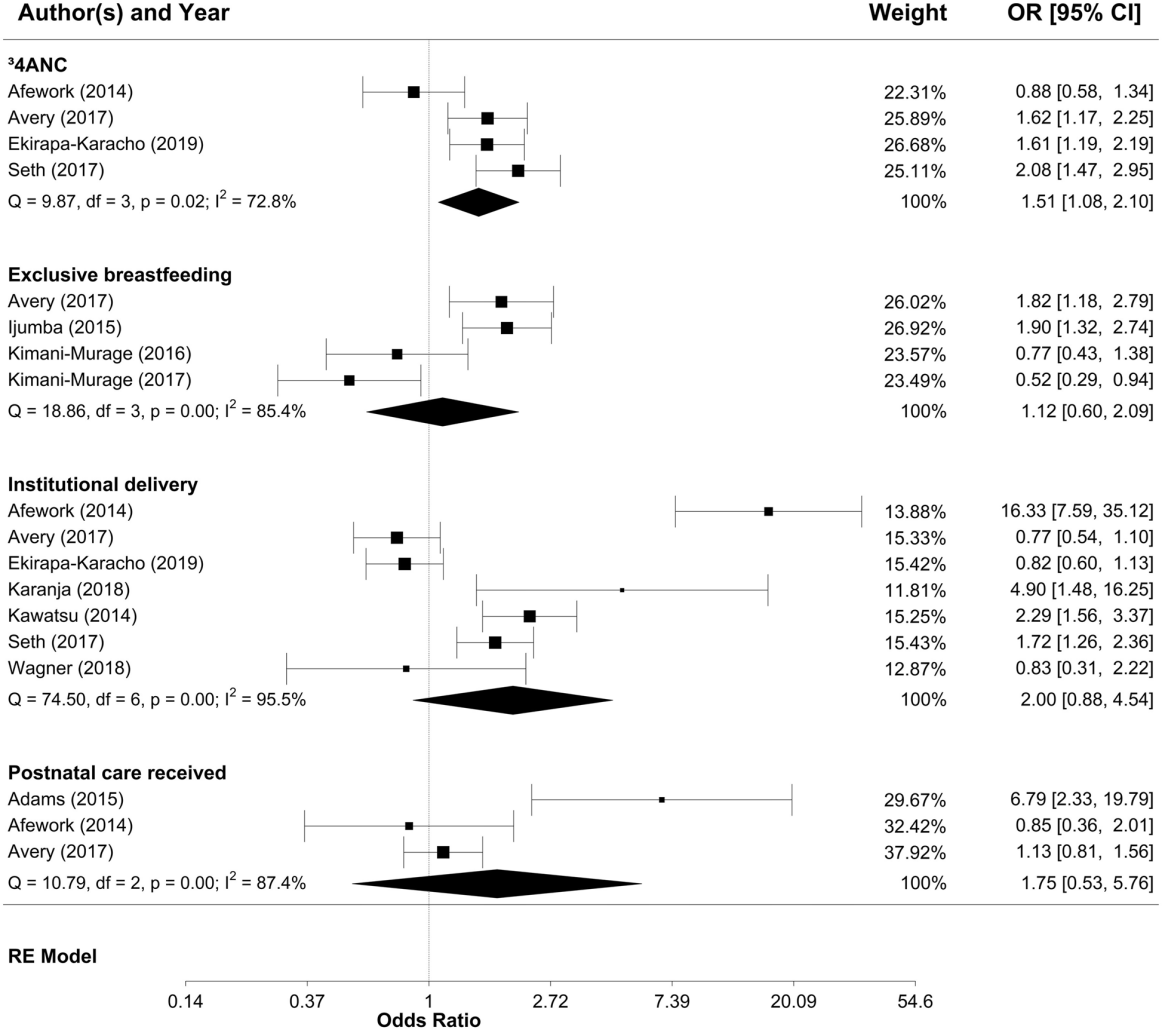
Meta-analysis of the associations of breastfeeding practices and utilisation of maternal health services with mothers’ SES

Findings on the impact of CHW programmes on socioeconomic inequities in health were less conclusive. Only 11 studies reported better outcomes in low-SES groups following CHW interventions [23, 28, 58, 76, 87–93], while 20 studies reported that people of lower SES showed poorer health outcomes and behaviours [27, 50–53, 56, 57, 76, 82, 85, 92, 94–102]. The remaining 19 studies reported no significant differences by SES [25, 27, 29, 32, 34, 56, 82, 85, 94, 95, 97, 103–110]. Meta-analysis showed that mothers of higher SES (as defined by household wealth and asset quintiles ranked as 1–5) were more likely to attend at least four antenatal care appointments (as per WHO recommendation) than mothers of lower SES (pooled effect:1.51, CI: 1.08–2.10), with no statistically significant differences for exclusive breastfeeding, institutional delivery, or postnatal care (see Fig. 6).

Qualitative findings highlighted a number of reasons CHWs may have limited or inconsistent impact on health behaviours and outcomes among low-SES community members. While CHWs mostly deliver services for free, the health professionals they routinely refer clients to often charged fees, which were one of the most ubiquitous barriers to taking up CHW health advice and referrals [111]. Clients feared having to pay for services, transportation, overnight accommodation, prescribed medications, or food should they be hospitalized [77, 112]. Even when medication and consultation fee waivers existed for the poorest community members, arduous and unreliable processes for obtaining these and restrictive quotas for distributing them were a barrier for some [66]. In one study, a number of clients sought to disengage from CHWs’ care when they were unable to get a fee waiver for their medications [41].

Beyond their lower uptake of facility referrals, low-SES individuals were sometimes unable to adhere to CHWs’ health advice and instructions. For example, one CHW explained how lack of food interfered with uptake of nutritional messages: ‘…when you counsel someone on what to eat, sometimes they look at you funnily because you can see that they cannot afford what you are telling them’ ([94], p. e432). In another case, CHWs’ educational messages about identifying infected pigs (a contributor to epilepsy) were ignored by farmers dependent on income from pig sales [113]. In the poorest families, all members might simply be too busy earning to visit health facilities or adhere to CHW guidance [112].

Multiple strategies were described for improving health outcomes for low-SES beneficiaries of CHW programmes. In some cases, CHWs played a role in negotiating with health services to accommodate clients’ SES, for example by writing ‘fee-free referral vouchers’ or accompanying the most vulnerable to facilities to ensure they received quality care without being erroneously charged [114, 115]. CHWs in one study recommended a triaging system to help health facility staff identify which patients were particularly poor and high risk and take advantage of their relatively rare visits to facilities [64]. Offering women incentives for facility delivery was also mentioned as an effective strategy, although it did not increase this behaviour in the poorest group [85].

#### Gender

Gender refers to ‘socially constructed roles and other traits that society generally associates with the sexes’ [10]. Although no eligible study reported on the experience of sexual and gender minorities (e.g. LGBTQI+), we found ample evidence on the reach and benefit of CHW programmes among women and girls, who face more barriers to accessing conventional services in some social settings. With regard to CHW service delivery, three studies found better coverage/uptake of CHW services among men [84, 116, 117], while six studies found no significant difference by gender in coverage or utilisation of CHW services [25, 32, 34, 79, 118, 119] and one study found improved coverage for females [119]. A more substantial body of evidence on CHW programme outcomes suggests that women/girls benefit equally, and in some cases more, from CHW programmes compared with men/boys. Fifteen studies found no significant gender differences in health outcomes or behaviours associated with CHW programmes [21, 32, 48, 49, 66, 100, 101, 104, 107, 110, 116, 120–123], while five studies found an advantage for females [50, 57, 123–125]. Only one study found worse outcomes for females [93].

Despite these encouraging findings, qualitative data suggest the story may be more complicated. Numerous qualitative and mixed-methods studies reported that women lacked agency over their own health and healthcare in ways that might not be captured in data on service coverage and outcomes. In many settings, women were expected to defer to their husbands in decisions about health, including decisions on when they could disclose having a disease, when and where to seek treatment, where to give birth, and how many children to have [44, 53, 72, 73, 111, 118, 126–130]. For example, participants in one study in Nigeria reported that, ‘Women who made independent health care decisions were considered to be arrogant, disrespectful and…“too forward”’ ([111], p. 72). Another study found that women with neglected tropical diseases sought care from CHWs later than men in part because of the threat of violence if they disclosed their symptoms [129]. The gendered distribution of childcare and other household work might also contribute to women’s inability to seek care outside the house [44].

Gendered constraints were particularly salient in the domain of sexual and reproductive health. In many settings the onus to prevent pregnancy was on women, while women’s limited financial and social autonomy made seeking out contraception difficult [131]. Young and unmarried women might avoid seeking family planning services from CHWs altogether for fear of stigma (e.g. being called ‘prostitutes’ [131];). Many women CHWs felt unable to discuss family planning with couples for fear of being ‘shouted at’, scolded, or accused by men of brainwashing young wives to abandon traditional gender roles [40, 128, 129]. In the starkest manifestation of gender inequity, interviewees in one qualitative study suggested a preference for boys led to greater utilisation of CHW services for male children compared with female children [73].

Interestingly, one study argued that hegemonic masculine norms in patriarchal settings have a detrimental impact on male health as well; concerns with demonstrating strength and power may inhibit men from seeking and utilising CHW services, particularly in the context of ‘emasculating’ illnesses such as HIV [132]. Moreover, the fact that many CHW programmes rely exclusively on female CHWs may be a barrier to men seeking sexual health services [39, 67, 129]. In Ethiopia, some men avoided CHWs’ health posts altogether because they were perceived to be a ‘woman’s space’; this had the additional effect of leaving their wives responsible for children’s healthcare, despite women’s limited access to money [67]. Men were generally more comfortable seeking family planning support such as condoms from male CHWs [133]. Yet in Afghanistan, social taboos prevented even male CHWs from distributing condoms to men [126].

A number of studies support a division of tasks between male and female CHWs as a strategy for improving equitability [39, 127, 134]. Female CHWs were described as a more accessible and acceptable source of information and care for women clients, particularly where sexual and reproductive health was concerned; male CHWs were at times better positioned to offer services to men as well as to provide some general services such as accompaniment to the hospital late at night [127]. Several studies reported that hiring male CHWs to act as ‘ambassadors’ to other men in the community on issues of reproductive and maternal health was an effective way to improve women’s access to needed care [40, 131, 135]. However, male CHWs in some studies also reported difficulties working with men [40, 127, 132]. Conducting home visits in mixed-gender pairs of CHWs may also be an effective strategy for reducing gender-related barriers to service delivery, including by countering suspicions that CHWs have ‘ulterior motives’ for visiting clients of the opposite sex [134, 135].

Home visits were one crucial way CHWs addressed health inequities experienced by women with restricted mobility [136]. EHealth/mHealth could also promote gender equity by allowing CHWs to consult with women confidentially [43]. With regard to family planning and reproductive health, some CHW programmes attempted to more directly engage with men as a way to facilitate women’s healthcare access [40, 131, 137]. In one programme, CHWs used general medical outreach camps as an opportunity to provide women family planning services, as husbands expected women and children to attend these routinely [138].

#### Education

A number of quantitative studies reported on differences in CHW service delivery and outcomes according to beneficiaries’ educational attainment. Eleven of these showed that CHWs delivered equal [21, 25, 31, 82, 90, 139] or better [22, 30, 32, 140, 141] service to less educated groups compared with more educated groups in terms of coverage, utilisation, and acceptability. Only three studies found that more educated individuals were more likely to receive or use CHW services [28, 84, 117]. However, findings with respect to CHW programme outcomes were once again less encouraging. Only three studies showed more positive CHW programme outcomes in less educated groups [32, 142, 143], while 24 studies reported more favourable health outcomes and behaviours among more highly educated beneficiaries [27, 35, 51, 52, 54–56, 59, 76, 80, 92, 94, 95, 99, 100, 105, 106, 110, 121, 143–147]. Twenty-five studies found that health outcomes or behaviours promoted by CHWs did not vary significantly by education level [45, 46, 50, 53, 56–59, 66, 88, 93, 97, 98, 103, 104, 107, 108, 110, 122–124, 139, 148–150].

**Fig. 7:**
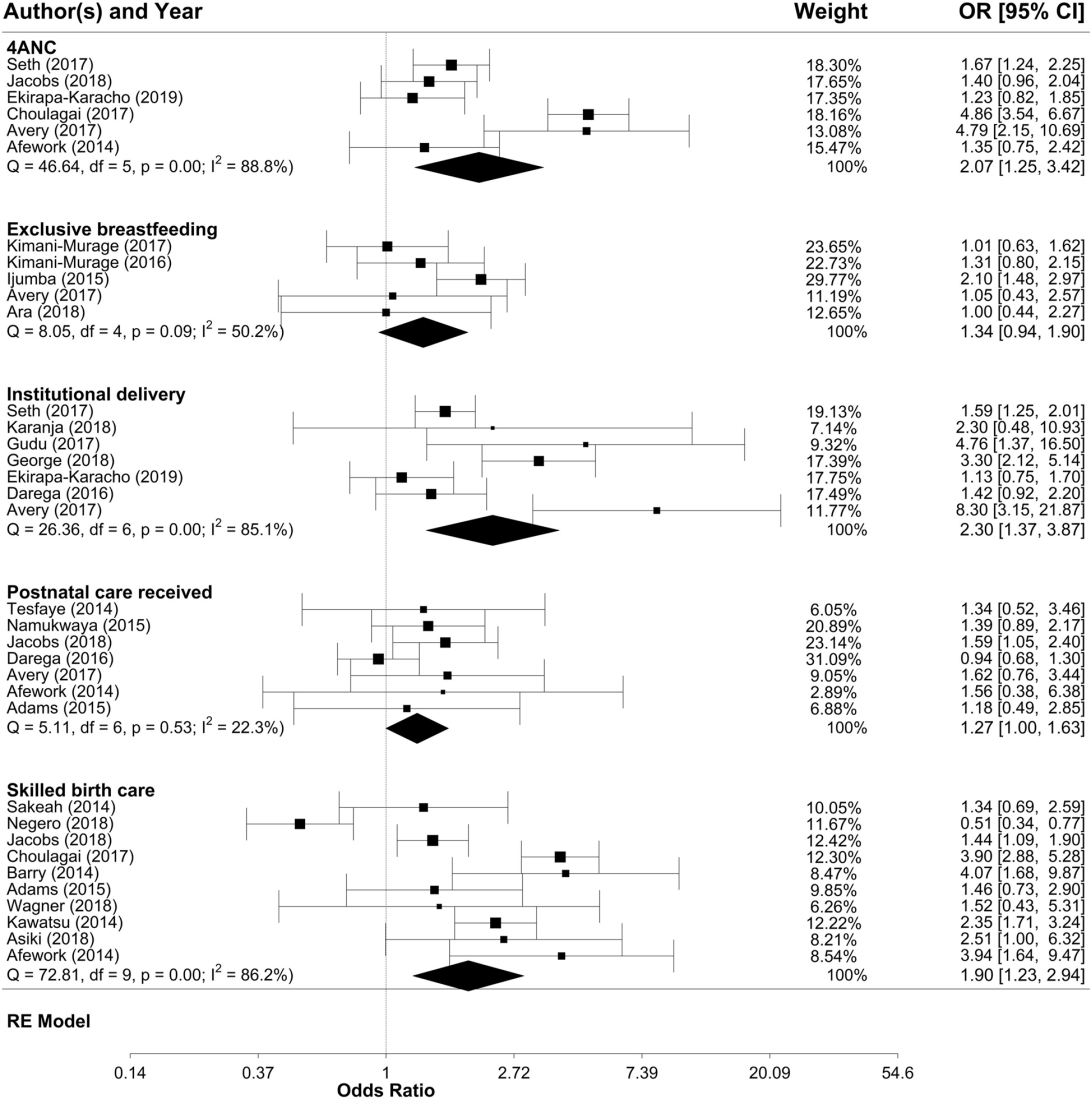
Meta-analysis of the associations of breastfeeding practices and utilisation of maternal health services with mothers’ level of education

Meta-analysis confirms that, in the context of CHW-delivered health promotion activities, mothers with at least secondary education had a greater likelihood of attending four or more ANC sessions (pooled effect: 2.07, CI: 1.25–3.42), having skilled birth attendance (pooled effect: 1.90, CI: 1.23–2.94), and institutional delivery (pooled effect: 2.30, CI: 1.37–3.87) than mothers with no education (Fig. 7). However, no significant differences in breastfeeding practices and PNC by maternal education were found.

Qualitative findings on education level were more limited. A few studies problematized CHWs’ use of text-based educational media, which were less likely to change the health behaviour of illiterate or low-literacy clients [42, 78]. In addition, low education levels contributed to CHWs’ perceptions of some social demographics as ‘ignorant’ or ‘backward’ with possible implications for quality of care. For example, one mother in Ethiopia complained that her local CHW ‘thinks we are ignorant and do[es] not care for our children’ ([67], p. 661). Strategies for overcoming education-related barriers included use of illustrated informational materials [151] and CHWs accompanying clients to health facilities when they did not feel able to express themselves to medical professionals [114].

#### Race/ethnicity/culture/language PLUS caste

This composite category in the PROGRESS framework addresses inequity based on membership in a particular cultural, linguistic, ethnic, or racialized group (emphasising that racial identity is socially rather than biologically defined); we additionally included studies reporting on caste and tribal group under this heading [10]. Although we did not identify any eligible studies reporting on language or racial identity, several studies investigated how well CHW programmes served historically marginalized caste, tribal, and minority ethnic groups. Of these, five showed coverage, acceptability, or utilisation of CHW services on par with that of non-marginalized groups [31, 32, 140, 147, 152], with two studies showing marginalized caste groups were more likely to access CHW services [21, 28]. However, findings were less encouraging with regard to inter-group differences in health outcomes and behaviours promoted by CHWs. Eight studies showed lower use of CHW-promoted services and behaviours among marginalized caste [96, 100, 122], tribal [32] and ethnic groups [35, 98, 104, 153]. Five studies found no difference between ethnic/caste/tribal groups [60, 93, 145, 147, 150], while only one study showed a pro-equity effect in favour of marginalized groups [91].

Qualitative findings on the healthcare experience of minority caste, ethnic, and tribal groups were sparse. In two studies ethnic minority participants described mistrusting or feeling looked down on by CHWs [118, 128]. More often, a confluence of education, age, language, place of residence and cultural factors seemed to act in concert to limit utilisation of biomedical services, including CHW services, by some social demographics. These groups were often glossed as ‘backward’ with little effort to unpack the different factors contributing to marginalization. Two studies mentioned lack of familiarity with the ‘culture of hospitals and biomedicine’ and biomedical explanations of affliction as a deterrent to CHW service utilisation and uptake of CHW referrals [42, 118].

Clients in a number of studies complained that CHWs looked down on them because of their cultural beliefs about heath, regarding them as ignorant, traditional, or bad parents [67, 118, 128]. Indeed, CHWs sometimes used these terms to talk about people who were difficult to engage or frequented traditional healers, conceiving their cultural practices as a barrier to service delivery. For example, one Ethiopian CHW described an encounter with an elderly pastoralist who resisted advice to boil her milk:

So instead of wasting time to this kind of people whose cultural issues are deep rooted, it’s better to talk to someone moderate in the family who can at least understand what I’m trying to tell him/her…. For us, dealing with rural people is a hell on earth ([25], p. 6)

These dynamics eroded trust and led to lower perceived quality of care and utilisation.

Recruiting CHWs from the same minority ethnic and language groups they serve and showing respect for traditions (e.g. by involving traditional healers in care) may promote equitable access and quality of care [53, 154]. For example, clients in a community-based care programme for indigenous Mexican women described language congruence as instrumental to their positive experience of CHW services [154]. However, one study reported mixed findings on the influence of CHWs sharing the same caste as beneficiaries [80]. CHWs’ accompaniment of minority or marginalized clients to health facilities may also help prevent mistreatment by other professionals [128].

#### Social capital

Social capital denotes an individual’s relationships and social networks [10]. We considered the following indicators of social capital: social capital quintile, social status, having CHWs as part of one’s social network, having spouse and in-laws as part of one’s social network, being a local leader or member of the village council, and level of social support. Data on how CHW programmes served those with low social capital were limited but suggest CHWs may be failing both to provide services in an equitable fashion and to reduce associated health inequities. The only study on coverage showed an inequitable bias towards serving individuals with high social status [32]. In terms of the impact of interventions on health inequities, two studies found greater uptake of CHW-promoted health behaviours among those with more social support [142] or who counted CHWs among their social networks [97], while in two studies there was no association with social capital found [32, 51].

The limited available qualitative data shed some light on why CHW programmes might fail to translate into better health outcomes and behaviours among those with low social capital. People with higher social capital had access to a social and financial safety net that enabled them to take up CHW advice and referrals to health services, for example by allowing them to raise funds needed for treatment through borrowing [42, 66, 155]. However, those community members most in need of financial support often had the lowest social capital because they were perceived as not being able to reciprocate [66]. Social stigma associated with abusing drugs could also lead to poor treatment of addicts at health facilities, deterring them from taking up referrals [114]. In one case, substance abuse was associated with neglect by CHWs [156].

One study described how CHWs helped to overcome challenges related to low social capital by accompanying patients to health facilities and writing them referral slips that encouraged health professionals to treat them appropriately [114]. However, in another study, people referred to health facilities by CHWs were taken less seriously due to CHWs’ own low status as an ‘informal provider’ [112].

#### Occupation

This section summarizes findings on possible disadvantage related to multiple dimensions of occupation, including unemployment, informal employment, and employment type. Five quantitative studies found comparable coverage and utilisation of CHW programmes across all occupational groups [32, 83, 90, 117, 149], while four studies reported lower trust or utilisation of CHWs among farmers, miners or the unemployed [22, 84, 157, 158]. Ten studies found no significant differences between occupational groups in CHW-promoted behaviours and outcomes [35, 45, 51, 52, 95, 106, 109, 121, 145, 147]. Among studies that did identify inter-group differences, three studies showed more favourable outcomes for the un- or informally employed [93, 104, 150] while two showed less favourable outcomes among farmers and the unemployed [98, 146]. One study reported mixed findings [45].

Qualitative findings revealed occupation and SES to be closely interrelated axes of marginalization. Occupation most often interfered with healthcare in poor families that were highly dependent on low-wage labour or subsistence farming. In such cases the opportunity cost of attending health-related appointments, particularly when this required travelling some distance, might outweigh the need for care [41, 159, 160]. Strategies to improve the accessibility of CHW services included adjusting CHW schedules to fit with those of working clients [36, 37, 41].

#### Religion

Findings on how CHW programmes served religious minority communities were limited and inconsistent. Available quantitative data on CHW service delivery suggest CHW programmes may be reasonably successful at reaching religious minorities. Four studies reported no statistically significant differences in coverage or utilisation of CHW services across religious groups [21, 28, 30, 96] and one reported an advantage for minority groups [84]. However, two studies reported a disadvantage for minority groups [32, 90]. Findings were once again less positive with regard to health outcomes and behaviours. While ten studies found no significant differences by religion in CHW-promoted health behaviours or outcomes [32, 35, 52, 57, 93, 107, 122, 141, 148, 149], six reported that members of religious minority groups – in most cases Muslims living in Christian- or Hindu-majority settings – fared worse on at least some indicators [21, 54, 95, 96, 100, 106]. No study reported better outcomes for religious minority groups.

Few qualitative studies reported on religion-related barriers to equitable engagement with and benefit from CHW services. Some studies reported that religious beliefs inhibited utilisation of CHWs who offered family planning as part of their package of services; while the choice not to engage with family planning for religious reasons is not indicative of inequity, this may have reduced religious minorities’ access to other CHW-provided services [61, 131]. A lack of sufficient female doctors led to lower uptake of CHW referrals for MNCH among religious minorities in India [73]. No strategies for overcoming religion-related inequities were reported.

#### Disability

Only one study reported quantitative data on CHW services in relation to disability, finding no association between disability and uptake of CHW referrals to mental health services [47]. Qualitative studies reported that visual impediments were a barrier to engagement with text-based education materials [42] and that CHWs were unable to communicate with the deaf [156]. In some cases, families refused CHW services for children with disabilities due to the stigma attached to disability [161]. Strategies for reaching and promoting health among those with disabilities include proactive community outreach via home visits to reach those unable to travel due to disability [66] and further training of CHWs on addressing the needs of community members with disabilities [161].

#### Intersectionality

Whilst we organized the above findings by equity stratifier to facilitate targeted policy recommendations, it is well established that in real-world settings different types of marginalization and oppression intersect to multiply disadvantage for certain groups [162]. Qualitative analysis revealed the importance of considering intersectionality in analyses of CHW programmes and health equity, most notably in relation to gender. For example, several studies found that gender intersected with SES to further marginalise poor women; women in many studies reported being financially dependent on their husbands and struggling to get care because of their husbands’ reluctance to spend money [67, 111]. Another challenge was women’s inability to make care decisions when their husbands were away, which was more common in poor rural regions where men were forced to migrate to earn [163]. Sometimes gender roles intersected with age and marital status, as when parents-in-law controlled decisions about the health of younger married women in the family [73]. Other reports of intersectional disadvantage highlighted the interrelationships among SES, place of residence, disability, and social capital [66, 131].

#### Summary of key findings and recommendations

Table 2 summarises, for each equity stratifier, the key findings from qualitative and quantitative studies and associated recommendations discussed above. Table 3 provides a synthesis of common programme elements found to be associated with pro-equity outcomes in the quantitative dataset, with examples of each. Although the successes of a programme in one setting do not necessarily translate straightforwardly elsewhere, the interventions that produced pro-equity outcomes all included *at least one* of these key elements: expansion of CHWs’ remit and scope of activities; increased training and monitoring of CHWs; addressing financial barriers to uptake of CHW advice and referrals; promoting effective partnerships between CHW and other stakeholders; and adapting programmes to local social and cultural contexts.

**Table 2:** Summary of key findings and recommendations on CHW services for disadvantaged groups

Group	Key findings on CHW services	Strategies for improving reach and impact
Rural & remote place of residence	• CHW programmes may help to attenuate urban-rural differences in healthcare access, but there are often differences in access to CHW services within rural areas due to large catchment areas (with those living closer to CHWs/health posts advantaged) • CHW services may be less accessible to migrant, mobile, and homeless populations • CHW referrals, education and promotion may reduce, but not fully overcome place-based differences in utilisation of professional facility-based services (especially MNCH services)* • Weaknesses in the wider health system exacerbate place-based differences and may undermine trust in CHWs charged with promoting health services	• Hire CHWs who live in rural and remote communities to serve these communities • CHW catchment areas should be manageable in size and account for local transportation infrastructure and difficulty of terrain • Where access to health facilities is very limited, consider expanding CHWs’ remit (e.g. to include more direct/curative service provision)^+^ • Provide torch light, radio, medical kits (containing essential medicines and equipment), mobile phones, and/or mobile phone credit to CHWs • Establish linkages with free or low-cost (emergency) transportation services^+^ • Provide bicycles or motorcycles to CHWs • Hire male CHWs in addition to female CHWs in settings where women’s mobility is restricted • Deploy ‘mobile’ CHWs in addition to community-based CHWs to reach nomadic and homeless populations • Strengthen transfer-of-care processes to facilitate continuous care when people move or migrate • Provide financial incentives or transport stipends for clients referred to health facilities^+^
Poor/low-SES	• CHWs services are generally equitable with respect to SES, with a number of studies reporting greater coverage and utilisation of CHW programmes by the poor • Low-SES clients experienced more barriers to taking up CHW referrals and health advice • There is some evidence that CHW programmes reduce differences in health across socioeconomic groups, but most studies showed that significant differences persist (especially for MNCH outcomes)*	• Provide CHW and other health services at low or no cost^+^ • Provide free or subsidized transportation to health facilities and home-based care where possible • Establish linkages with free or low-cost transportation services^+^ • Provide financial incentives for CHWs to identify and serve the poorest^a^ • Incorporate food parcels and social welfare grants within CHW services • Empower CHWs to provide ‘fee-free referral vouchers’ to low-SES clients in need of facility-based treatment • Establish CHW triaging system to flag poor, high-risk patients to facility-based professionals for immediate attention (preventing need for costly overnight stay and repeat visits)
Women & girls	• Quantitative data suggest that CHW programmes provide comparable coverage and have similar effects on health outcomes in males and females • Qualitative data revealed that many women still lack agency to make their own decisions about when and how to engage with CHW and other health services (especially for sexual and reproductive health needs)	• Provide home-based care/home visits where possible (countering women’s restricted mobility) • Use mHealth to facilitate confidential consultations for women • Embed family planning services within general medical outreach camps • Hire male CHWs to engage with men and act as ‘ambassadors’ on women’s health rights and needs • Strategic distribution of tasks between male and female CHWs • In some cases, deploy CHWs in mixed-gendered pairs for home visits
Illiterate/ less educated	• CHW services are generally equitable with respect to less educated groups in terms of coverage, utilisation, and acceptability • There was some evidence of better MNCH outcomes when women or their husbands were more educated*	• Use illustrated (rather than text-based) informational materials • Use other non-textual locally appropriate channels for behaviour change communication (e.g. radio, mobile video drama)^+^ • Arrange for CHWs to accompany clients to health facilities
Marginalized caste, tribal, and ethnic groups	• CHW services were generally found to provide comparable coverage, acceptability, and utilisation for marginalized and non-marginalized groups • CHW may provide lower quality services to groups they see as ‘backward’, ‘traditional’, or adhering to cultural beliefs about illness • Marginalized caste, tribal, and ethnic groups may be less likely to take up CHW referrals and health advice	• Recruit CHWs from ethnic and linguistic minority groups • Show respect for cultural traditions in CHW service provision, e.g. by involving traditional healers in care • CHWs can accompany clients to health facilities to advocate on their behalf and prevent mistreatment
Low social capital	• There is some evidence that CHWs provide more services to those with greater social capital or whom are part of their own social networks • Those with low social capital may have reduced ability take up CHW referrals to health centres	• Arrange for CHWs to accompany clients to health facilities • CHW referral slips that facilitate access to facility-based services
Un- & informally employed	• CHWs appear to serve all occupational groups equally, although there was some evidence of lower trust and utilisation among farmers and the unemployed • In families that are highly dependent on low-wage labour or subsistence farming, the opportunity cost of attending health-related appointments may be a barrier to engagement	• Schedule CHW working hours to accommodate clients’ work commitments (e.g., evenings and weekends)
Religious minorities	• Findings were inconclusive, though there is some limited evidence of persistent poorer health outcomes for religious minorities (mainly Muslims living in Christian- or Hindu-majority countries)	• None reported
Disabilities	• Limited data available	• Provide home-based care/home visits to overcome travel-related barriers • Train CHWs on physical and mental disabilities

^*^Supported by findings of meta-analysis

^+^ Common element of interventions with pro-equity outcomes in quantitative data set (see Table 3)

^a^Some important limitations of this approach are discussed in Section “Socioeconomic status”

**Table 3:** Common elements of CHW programmes associated with pro-equity outcomes

Programme element	Examples of programmes with pro-equity outcomes
Expanding CHWs’ remit	Bangladesh: CHWs providing skilled birth assistance and ANC resulted in increased ANC attendance (≥ 4 visits) and use of SBAs, with the greatest improvements in hard-to-reach locations [56]. Mozambique: CHWs promoting, diagnosing and treating childhood illnesses resulted in early care-seeking behaviour (within 24 h of onset) in lower SES groups [90].
Increased CHW training & mentoring	Ghana: Enhanced CHW training for assessment and referral of newborn illnesses and follow up with addressing barriers to compliance was associated with higher compliance with referrals and doubled independent care seeking for newborn illnesses in women in the poorest quintile [23].
Addressing financial barriers	India: Cash transfers to women for institutional delivery and to CHWs for conducting ANC led to an increase in ANC attendance and facility delivery 5–6 years later, with the largest increase among women of low SES and educational attainment [88]. Uganda: reducing cost outlay for CHW-provided services led to improvements in care-seeking for childhood illness among lower SES groups [91].
Promoting effective partnerships	Ethiopia: Effective collaboration between trained CHWs and unpaid volunteers led to increased use of SBAs and PNC, and decreased use of untrained providers or no provider, with the greatest improvements for women of lower SES [58].
Adapting to local contexts	Ethiopia: Use of locally appropriate channels for behaviour change communication (e.g. radio spots, mobile video drama) and adopting local solutions for pregnancy identification, registration, birth notification (+ extended service provision + ongoing training and mentoring) were associated with better care seeking for pregnancy complications, specifically in lower SES groups [89]. Nigeria: Development of more practical and user-oriented workshops were associated with greater likelihood of use of bed nets among people with lower levels of formal education [142].

### Beyond service delivery: additional pathways to addressing health inequities

As Blanchard and colleagues [7] observed, the guiding assumption behind most CHW programmes in LMIC is that expanding access to health information and services within marginalised groups is the key to achieving health equity. However, our analysis of qualitative findings identified three *additional* pathways through which CHW programmes may influence health equity in the populations they serve.

#### Advocacy

First, CHWs in some countries have become involved in advocacy to address social, political and structural problems that lie at the root of health inequities. At times, this work was recognized and built into formal CHW roles. For example, CHWs in the Mitanin programme in India have developed an identity as agents of social change and have a successful track record of taking action on social determinants of health [164]. Like Mitanins, Indian ASHAs are expected to do advocacy work around clients’ rights and entitlements, although one study found ASHAs were confused about their responsibilities as activists [65]. Likewise, CHWs at Casas de Maternas in Mexico aspired to take on structural determinants of health by strengthening and claiming the rights of indigenous women [154].

In other cases, CHWs went beyond their designated roles to advocate for change. In one study, Tanzanian grassroots volunteers noted that clients were not adhering to treatment due to food insecurity and successfully lobbied the NGO employing them to add food distribution to their portfolio of services [86]. In another compelling example, a group of women CHWs in South Africa used a participatory action research project to challenge gender inequalities contributing to violence against women in their community [165].

#### Personal investments by CHWs

There was also considerable evidence of CHWs going beyond formal roles to invest their own personal resources (financial, physical, social and emotional) in bridging equity gaps. The data set was replete with examples of CHWs spending their own money for clients’ treatment or transport to health facilities, even when they had limited means themselves (‘the last money in my pocket’ [65 p. 390]; [69, 70, 72, 120, 151, 195]). CHWs in some studies emphasised the difficult position they found themselves in providing frontline, often home-based care to society’s most vulnerable; as one CHW in South Africa explained:

They advise us that, when we encounter a difficult situation, we must also consider ourselves. But you cannot ignore a situation when you meet a sick patient who does not have food and has not yet received the [disability] grant. You do [have to] provide the patient with something that you have ([64], p. 387)

Some CHWs also physically carried ill clients to health centres [64], tried to raise funds for clients’ treatment from others in the community [41, 163], brought food to their clients [86, 166], or walked long distances on foot carrying heavy medical supplies to reach clients [62]. CHWs routinely paid for their own mobile airtime credit or charging to call clients, and sometimes had to travel to get mobile service [43]. Some female CHWs risked the threat of violence by providing family planning services to women in secret [126, 138]. Others leveraged their social capital by getting involved in clients’ family disputes, for example, to advocate for a woman’s right to treatment [44].

While providing such care was described as rewarding for some [86, 136], the personal investment this required sometimes had a negative effect on CHWs’ own health and wellbeing. CHWs reported neglecting their own farms to help clients [75, 159]. In a Tanzanian programme where grassroots volunteers often brought flour to cook for ill clients, many had to quit because of the declining economic situation of their own families [86]. CHWs expressed feelings of frustration about their inability to overcome the many barriers militating against health equity, and the cases in which they were unable to help weighed on them [136, 166]. Moreover, CHWs sometimes found themselves blamed for wider systemic and structural failures affecting the most vulnerable – for example, when they encouraged clients to exercise their rights to free services or fee waivers that the public health system subsequently failed to deliver on [63, 75].

#### CHW hiring and employment practices

Finally, experience from a number of studies suggests CHW programmes can contribute towards addressing social determinants of health by making stable and well-regarded jobs available to members of disadvantaged groups. In particular, women CHWs may experience empowerment through their work as they become financially autonomous and respected in their communities [127, 130, 154]. A female CHW in Mexico, for example, described how her work encouraged her to stand up to gender-based violence in her own home:

The truth is that I’ve talked with them [women service recipients], told them not to allow themselves to be treated that way, because before, before we put up with the hitting. It even happened to me and I was one of them… Since I talked with them, I stopped being frightened and my children too. They say ‘it’s okay mom, because you can defend yourself (survive) on your own.’ ([154], p. 5)

Other studies suggest that employing CHWs from the poorest and most stigmatized social groups can challenge structural inequalities and improve the health and wellbeing of CHWs and their families [70].

At other times, however, CHW programme employment practices failed to address, or even exacerbated, the social and economic inequalities that underpin health inequities. CHWs were often from low SES backgrounds and many reported financial hardship compounded by the indirect and direct costs of their work and inadequate remuneration [65, 77, 130, 167]. CHWs complained of having insufficient opportunities to get further education and training, with clients and health professionals often looking down on them for their low education levels [75, 130, 159, 168].

In some programmes, there was apparent discrimination *between* CHWs. Female CHWs were sometimes treated differently than male CHWs – for example, being expected to volunteer their time without remuneration or deprived of training and material incentives males received [126]. Unmarried female CHWs reported gender and age discrimination, while those married struggled to balance poorly remunerated CHW work with a heavy burden of domestic responsibilities [37, 169].^4^ Some CHW employment practices also replicated existing patterns of place-based disadvantage. For example, in Malawi, rural CHWs received less support in terms of training and incentives than urban CHWs, and yet they were expected to deliver services in a far more challenging context [170]. CHWs in remote communities complained of having to travel long distances carrying heavy supplies and missing out on opportunities for feedback and training available to CHWs living closer to health facilities [62, 130, 171]. Such disparities in access to training and resources have obvious detrimental implications for the disadvantaged communities these CHWs serve.

## Discussion

In recent years, CHWs have been looked to as a panacea for global health inequities. This review offers the most comprehensive and up-to-date account of the evidence on whether and how CHW programmes in LMIC are living up to this vision. Broadly speaking, our review findings support the implementation of CHW programmes as a strategy for extending healthcare access to hard-to-reach groups. Although findings were mixed, a majority of studies reported that coverage and utilisation of CHW services were either comparable across groups or greater among disadvantaged groups. At the same time, our analysis raises important questions about the implicit assumption underlying many CHW policies and programmes that ‘equity is achieved once everyone has access’ [7]. Below, we summarise key findings and discuss strategies for improving the impact of CHW programmes in disadvantaged groups, based on a reconceptualization of the ways that CHWs can contribute to more equitable health outcomes.

### Are CHWs reaching and improving health in disadvantaged groups?

Our review found compelling evidence that CHWs are effective at reaching several groups that experience barriers to accessing conventional health services, including rural dwellers, women and girls, the poor, and those with limited literacy/education. This was reflected in levels of CHW programme coverage, utilisation, acceptability, and accessibility in these groups that were largely comparable with, or in some cases greater than, those of more privileged groups. This is broadly consistent with the findings of McCollum et al.’s [6] earlier review. However, CHW programmes do not appear to be reaching *all* marginalised groups effectively, with those in the most remote areas consistently missing out. Moreover, our findings also suggest that enhanced access to and utilization of CHW services did not always translate into better health outcomes and behaviours for marginalised sectors of the populations served. Disadvantaged groups often showed significantly poorer health behaviours and outcomes post-intervention than more privileged groups. Although most of the studies used designs that precluded causal inference, the subset of high-quality RCTs indicated a similar picture, with relatively few (2/18) reporting pro-equity outcomes. Meta-analysis also found poorer MNCH outcomes for those with lower SES, less formal education, or who lived farther from health facilities, confirming the overall picture.

Qualitative data illuminated some of the reasons why this might be the case. Most notably, members of some disadvantaged groups were less able than their more privileged counterparts to follow CHW advice and take up referrals to other services. For example, educating parents about proper child nutrition proved futile in cases where families could not afford sufficient food, while sometimes CHW health advice came into direct conflict with people’s livelihood strategies. Likewise, CHWs might reach and educate poor women living in remote areas about the importance of facility delivery but, unless affordable transportation to facilities is available, these women will have little option other than to continue giving birth at home. The cost and poor quality of services available at health facilities, as well as reportedly discriminatory treatment by health professionals, also appeared to disproportionately hinder disadvantaged groups from taking up CHW referrals, including marginalized ethnic/tribal/caste groups, rural dwellers, and the poor. In some cases, a single past experience of mistreatment at a health facility might be enough to dissuade a poor, rural dwelling client from agreeing to shoulder the high costs (direct and indirect) of returning to health facilities a second time. These observations are consistent with Blanchard et al.’s [7] finding that CHW programmes had a less equitable impact on care seeking in formal health services than on utilization of CHW-delivered home-based care practice.

Qualitative data also cast some doubt on the interpretation of quantitative findings on equitable service delivery, especially with regard to gender. While women may access CHW services in similar proportions to males, this is not necessarily on their own terms, as men in many settings continue to control when and how women utilize healthcare. More research is also needed to understand how well CHWs meet the needs of individuals with disabilities, religious minorities, sexual and gender minorities, marginalized ethnic/caste/tribal groups, and some occupational groups.

Taken as a whole, these findings corroborate those of Blanchard and colleagues’ previous review [7], which also found little evidence that CHW interventions can overcome the effects of structural determinants of health such as poverty and geographic marginalization. Although CHWs are often conceptualized as links between communities and formal health services, in practice they have limited influence over many of the factors that impede people from accessing these services, including poor road conditions, long distances, and the cost and quality of care provided by health professionals. In other words, providing health education and information may not be enough to change the practices and health-seeking behaviours of marginalized populations, without complementary investments in poverty alleviation measures, improving transportation infrastructure and health system strengthening. In settings where people grapple with both geographic and economic marginalization, there may also be a need to revaluate the weighting of CHW responsibilities towards health promotion and prevention and consider expanding CHWs’ remit to include more direct/curative treatment [3, 59, 66].

### Reconceptualizing pathways to health equity

The findings of this review suggest that the conventional wisdom on CHW programmes can both over-estimate and under-estimate their potential impact on health inequities. The over-estimation comes from a failure to recognise the significance and weight of structural drivers of health inequities. While well-designed CHW-delivered services may go some distance toward addressing these, they are unlikely to fully overcome the systemic challenges experienced by the most disadvantaged unless accompanied by substantial complementary investments in poverty alleviation, health systems reform, transport infrastructure, etc. This is consistent with Blanchard et al.’s ([7], p. 9) identification of an ‘urgent need to support CHWs’ efforts by addressing, rather than compensating for, gaps in formal health services’ accessibility, availability, quality and affordability’ and the recent WHO guideline, emphasising that CHWs should be viewed as an *integrated element of* rather than a *cost-saving substitute for* functioning primary healthcare services [2].

However, our analysis suggests that conventional wisdom also *underestimates* the potential contribution of CHWs by viewing them in narrow, instrumentalist terms as simply ‘an extra pair of hands’ delivering ‘technical fixes’ [172]. There is a small but compelling body of evidence that CHWs’ contribution can go beyond bridging existing healthcare access gaps, to addressing *upstream* causes of disparities in vulnerability and access. Three pathways in particular deserve attention.

First, CHWs’ positioning within communities gives them unique insights into where programmes are failing to serve marginalized groups equitably and strong motivation to address these failures, sometimes by investing personal resources [44, 60, 63, 91, 109, 155]. This on-the-ground expertise and commitment should be recognised and harnessed by bringing CHWs into the heart of planning processes. Where appropriate, CHWs should be supported in taking their own initiatives to respond to the challenges they encounter, while ensuring that they are not bearing unreasonable personal costs to the detriment of their own wellbeing [67, 173]. Second, and relatedly, CHWs can play important advocacy and activist roles (formal and ad hoc), challenging social inequalities, enforcing respect of rights, and calling for health system reform [154, 164, 165]. Following Kane [172], we call for CHWs to be recognised as skilled and agentic forces within communities and health systems, and for appropriate and supportive working environments that enable them to realize this potential [67, 161].

Finally, CHW programmes can contribute to addressing social and structural inequalities by creating qualified employment opportunities within disadvantaged communities [2, 174]. Adequately remunerated employment that is meaningful and impactful can be a pathway to improvements in health and wellbeing for CHWs from low-SES backgrounds [70]; it can also help to address social inequalities and promote empowerment, especially for women from disadvantaged groups [127, 175]. Unfortunately, however, our review found substantial evidence of poor working conditions for CHWs, including unstable employment, inadequate remuneration, and discriminatory treatment of rural-dwelling, minority and female CHWs. Recent research suggests such working arrangements act as barriers to CHW empowerment, fostering feelings of being ‘unsupported, underappreciated, and undervalued’ [174]. Our findings thus add to a growing literature on how CHW programmes may inadvertently reproduce the very social and economic inequalities they set out to address [176–183]. To optimise their impacts, CHW hiring practices and working conditions need to redress, rather than replicate these wider patterns of inequality [2, 184].

**Fig. 8:**
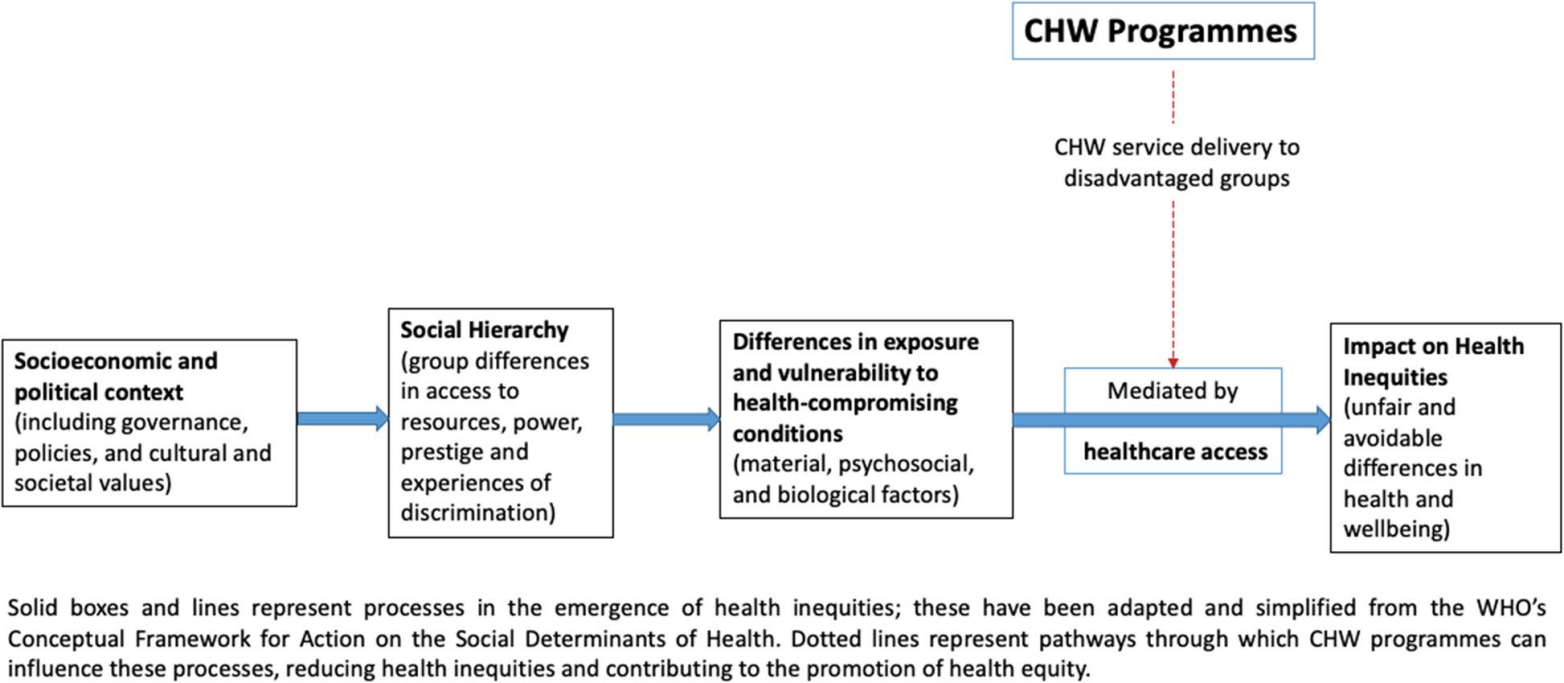
Conventional thinking on CHW programme contributions to health equity

**Fig. 9:**
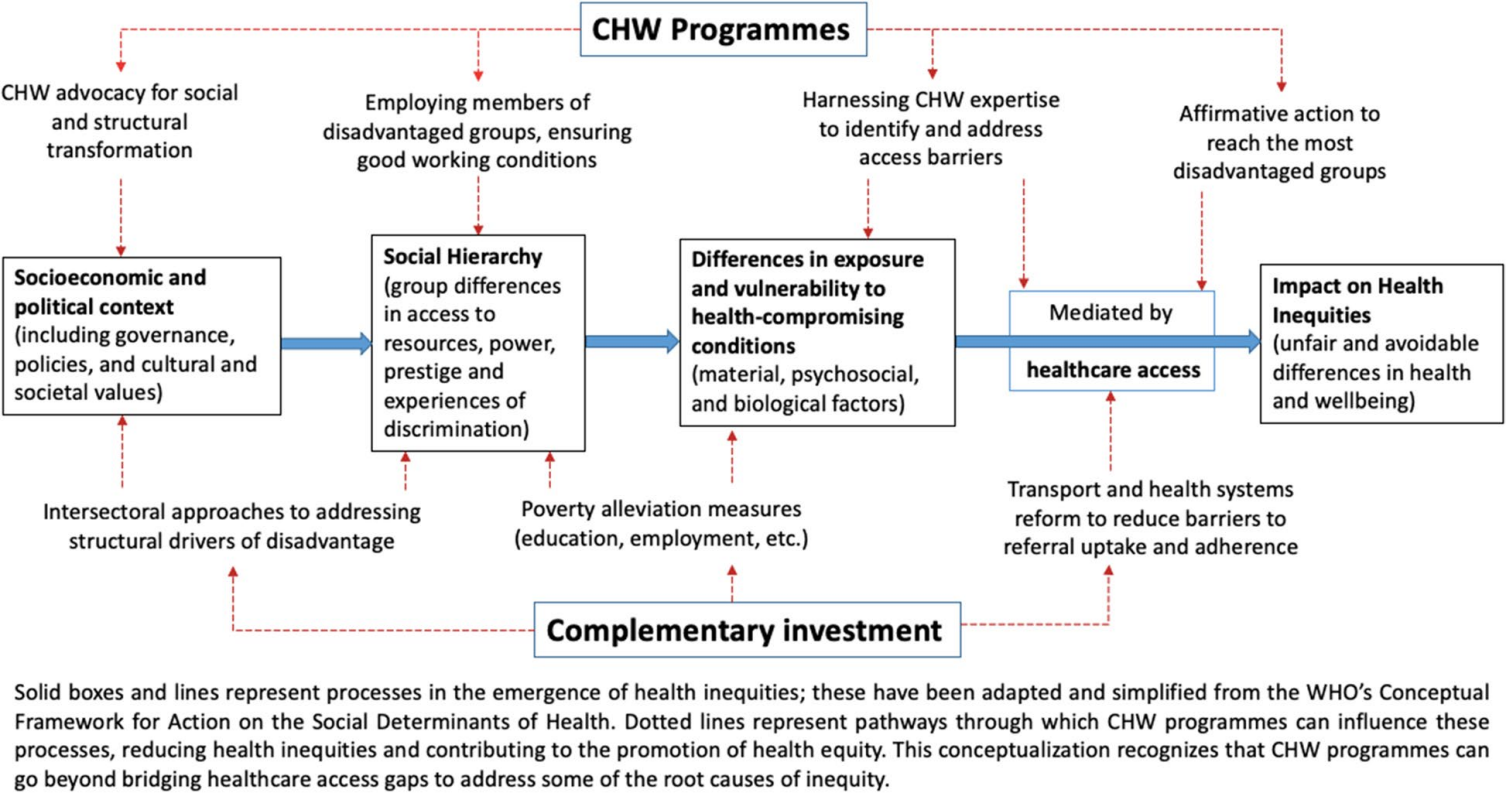
Reconceptualizing CHW programme contributions to health equity

**Fig. 10:**
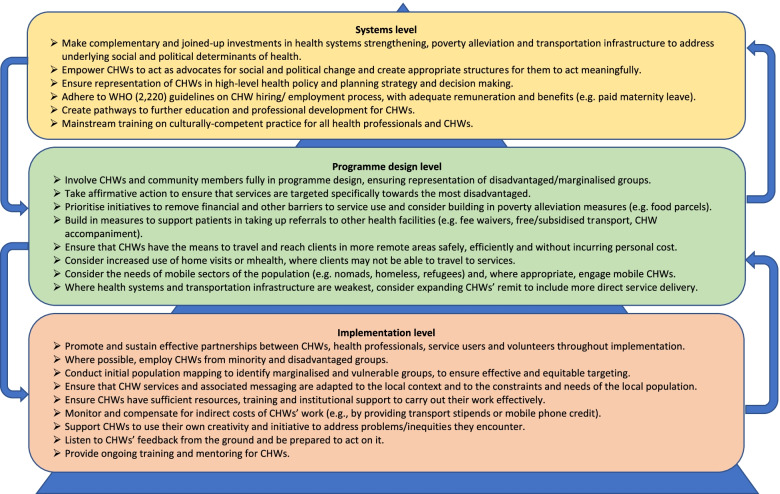
Optimising equity impacts of CHW programmes: an integrated, evidence-based approach

Taken together, the evidence reviewed here suggests the need to think differently about CHW contributions to health equity, based on two key insights. First, sustained wider investment in health systems reform and poverty alleviation, and sustained inter-sectoral working, will be required for CHW interventions to reach their full potential. Second, by confining the role of CHWs to service delivery, we are missing important opportunities to address upstream causes of disparities in vulnerability and access. In other words, CHWs have the potential to rise above serving as a temporary sticking plaster in settings of deep, existing inequity, to play a role in catalysing social, political, and health system transformation. Figures 8 and 9 illustrate this reconceptualization, based on the WHO’s Conceptual Framework for Action on the Social Determinants of Health [185]. Figure 10 then presents recommendations for optimising the equity impacts of CHWs programmes in light of this broader conceptualization of available evidence.

### Strengths and limitations of the evidence base

#### Inclusions and exclusions

The large number of studies captured in this review compared with previous reviews [6–8] reflects both modifications to search terms and eligibility criteria employed to capture the widest possible evidence base and the burgeoning interest in CHW programmes and health equity in recent years [5]. However, studies not published in English were excluded from the review, which may have led to under-representation of data from certain world regions (e.g. francophone/lusophone Africa and Latin America). It is notable that the geographical distribution of studies captured in the review is very uneven, with more than a third coming from just two countries (Ethiopia and India) and another quarter coming from just four countries (Kenya, Uganda, South Africa and Bangladesh). It is not clear why this is the case but there are clearly implications for the generalisability of the review findings. Moreover, the published literature captures only a small proportion of the vast number of CHW programmes implemented worldwide by governments and NGOs that have not been subject to rigorous research. Future studies might consider exploring axes of marginalization beyond those captured in the PROGRESS framework, for example, citizenship/nationality or migrant/refugee status.

#### Evidence gaps

Our analysis revealed several notable gaps in the evidence base on CHWs and health equity. Five years on from McCollum and colleagues’ review [6], there remains a dearth of studies reporting on CHW service *quality* for disadvantaged groups. Another important outcome rarely considered is the impact of programmes on the rights, needs and wellbeing of CHWs [4]. We also noted that most available literature focused on stable development contexts, lending support to Gilmore and colleagues’ [186] claims of a pressing need for more evidence on CHWs in humanitarian settings. The health issues addressed most frequently in included studies were MNCH and infectious diseases (e.g. TB, HIV). Despite the rise of non-communicable diseases across the Global South and their prominence in the SDGs, there remains a serious gap in the evidence base on how well CHWs address these conditions in disadvantaged groups.

With regard to equity stratifiers, the evidence base is most robust for place of residence, gender, education, and SES. There was a notable dearth of evidence on how CHWs serve those with disabilities. This may in part reflect stigma, conflicting definitions of disability, and a lack of data on or registration of disability in many LMIC [187]. In addition, we found a concerning absence of research on how CHWs serve sexual and gender minorities, likely due to high levels of stigmatization and criminalization of sexual and gender diversity in many LMIC [188]. Research on how CHWs serve linguistic, religious, and ethnic minorities was also limited, perhaps reflecting a common assumption that CHWs share a common language and culture with their clients despite the enormous social diversity that exists within many LMIC communities [189]. Some studies suggest the social identities of CHWs may play a role in determining the access of disadvantaged groups; it would be helpful to explore this in future reviews [189, 190]. Finally, qualitative findings and social theory suggest different axes of marginalization intersect to multiply disadvantage; there is a need to develop modes of collecting and reporting quantitative data on CHW interventions that allow for the capture and analysis of these interactions.

#### Study quality, reliability, and heterogeneity

As noted above, we tailored search terms and eligibility criteria to capture the largest possible evidence base. However, equity was not the primary outcome for most of the quantitative studies reviewed, which limited in some cases the scope of available data. Moreover, high levels of heterogeneity in data collected and reported limited our ability to extract and synthesize quantitative findings relevant to equity; as such, it was only possible to conduct meta-analyses for a limited set of variables. Our findings thus support calls for incorporating greater attention to all dimensions of equity in the design, implementation, and evaluation of CHW programmes [2, 7]. In the future, it would be helpful to devise a common set of equity indicators and standardized reporting guidelines for CHW intervention evaluation.

The majority of the quantitative studies reviewed were assessed as being of good quality, but relatively few had strong causal designs. Altogether, only eight studies met the ‘gold standard’ of randomised controlled trials (RCTs) with low risk of bias. Twenty studies (of which 17 were ‘good quality’) used pre-post comparison, while most were cross-sectional, making it very difficult to infer causality. The subset analysis of the eight RCTs with low risk of bias shows a broadly similar pattern of findings to the full set of studies, but there is clearly a need for more high-quality studies with good baseline data and effective controls to improve the robustness of the evidence base. This is particularly problematic when trying to assess health outcomes associated with programmes, where no credible baseline data are available. In most cases, it was also not clear whether or how the studies had been powered statistically; as such, it is often not possible to know whether apparent equality of service coverage and outcomes were ‘real’ effects or just the consequence of an under-powered study. Most of the qualitative studies reviewed were ranked as ‘thin’ or ‘satisfactory’ during quality appraisal, suggesting a lack of methodologically-rigorous and conceptually-rich qualitative evidence on equity issues in CHW programmes. We therefore second Maes et al.’s [191] call for further ethnographic research to illuminate the complex relationships between CHWs and the communities they serve.

**Table:** 

**Box 1. Recommendations for Strengthening the Evidence Base on CHW Programmes and Health Equity** • Incorporate equity analyses in routine CHW programme evaluations • Adopt common indicators and procedures for reporting on equitability of CHW programmes • Further research is needed on whether and how CHWs serve linguistic, ethnic, religious, sexual and gender minorities; those with disabilities; and those suffering from noncommunicable diseases • More in-depth qualitative and ethnographic research is needed to understand the mechanisms through which CHW programmes influence health equity as well as possible unintended consequences (e.g. impacts on CHW wellbeing) • Explore how CHWs’ own social identities influence access, utilization, and quality of care for disadvantaged groups • Account for intersectionality in research on CHW programmes and health equity	

## Conclusion

This systematic review synthesised findings on CHW programmes and health equity in LMIC from 167 studies published in the last 6 years, presenting an important update of previous reviews. The evidence reviewed here confirms the findings of previous systematic reviews [6–8] that, broadly speaking, CHW programmes have been effective in reaching disadvantaged segments of the population, extending healthcare access to those in rural areas, those with limited formal education, those of lower socioeconomic status, and other marginalised groups. However, our findings suggest that equitable CHW service delivery alone cannot fully compensate for the barriers to health experienced by society’s most disadvantaged. In particular, some marginalized groups were less able to take up CHW health advice and referrals to formal health services.

It is worth recalling the precise parameters of this review, which has focussed on equity *within* populations served by CHW programmes, rather than the effectiveness of CHW programmes overall or their impacts on wider (global) health inequities. Three points in particular are worth clarifying. First, an ‘equity-neutral’, or even an ‘anti-equity’ programme, may still improve the health of disadvantaged sectors of a population in absolute terms, even if the benefit to more privileged groups is proportionately greater. Second, and relatedly, the fact that CHW programmes do at least *reach* disadvantaged and marginalised groups suggests that they are an improvement on the status quo of relying on inaccessible formal-sector services. Finally, most CHW programmes are targeted towards disadvantaged populations and regions; any resulting health gain, even if inequitably distributed *within* that population or region, will still contribute to reducing health inequities on a larger (global) scale.

Nonetheless, it is clear that more must be done to optimise CHW programme contributions to health equity. Based on the available evidence, we suggest that this will require substantial complementary investments in health system strengthening, transportation infrastructure and poverty alleviation, as well as providing fair working conditions for CHWs and empowering them to take initiative and advocate for change. Crucially, we need to move beyond seeing CHWs as a temporary sticking plaster, and instead to build meaningful partnerships between CHWs, communities and policy-makers to confront and address the underlying structures of inequity.

## Supplementary Information

**Additional file 1.** Search Strategy: provides an example of the search strategy used in one database.

**Additional file 2.** Qualitative Synthesis Coding Framework: provides the coding framework used in thematic content analysis of qualitative evidence.

**Additional file 3.** Characteristics of Studies Included in Qualitative Synthesis: table including study characteristics and methodological quality ratings for included studies containing qualitative evidence.

**Additional file 4.** Characteristics and Findings by Outcome and Equity Stratifier of Studies Included in Quantitative Synthesis: a series of tables including study characteristics and methodological quality ratings for included studies containing quantitative evidence, along with details of each study’s findings by outcome and equity stratifier.

**Additional file 5.** Funnel Plots to Investigate Publication Bias: Presents funnel plots that were used to investigate publication bias.

## Data Availability

Data extracted from the articles included in the current study are available from the corresponding author upon reasonable request.

## Abbreviations

ANC: Antenatal care
CHW: Community health worker
LMIC: Low- and middle-income countries
HIV: Human immunodeficiency virus
MNCH: Maternal, newborn and child health
NGO: Non-governmental organization
PNC: Postnatal care
RCT: Randomised controlled trial
SBA: Skilled birth attendant
SDG: Sustainable development goal
TB: Tuberculosis
WHO: World Health Organisation

## Footnotes

1 There is considerable debate over whether to appraise quality in syntheses of qualitative data [192–195]. Evidence suggests that unprompted judgments by qualitative experts are less time-consuming and achieve similar inter-rater agreement to judgments based on available structured checklists [15, 192, 193].

2 Some papers reported findings on multiple outcomes; only two studies reported ‘mixed’ findings and have been excluded from Figures 3, 4.

3 Following the PROGRESS framework, we considered the effects of social capital, occupation (including employment status), and education independently wherever possible [10], however, some studies cited in this section reported only composite measures of SES and it was impossible to discern the independent effects of education, social capital, and/or employment status.

4 For a discussion of how gender relations impact the working lives of CHWs, see [127].

## References

[1] Tulenko K, Møgedal S, Afzal M, Frymus D, Oshin A, Pate M (2013). Community health workers for universal health-care coverage: From fragmentation to synergy. Bull World Health Organ.

[2] WHO (2018). WHO guideline on health policy and system support to optimize community health worker programmes.

[3] WHO (2008). The world health report 2008: primary health care now more than ever.

[4] Scott K, Beckham S, Gross M, Pariyo G, Rao K, Cometto G (2018). What do we know about community-based health programs? A systematic review of existing reviews on community health workers and their integration with health systems. Hum Resour Health.

[5] Cometto G, Ford N, Pfaffman-Zambruni J, Akl EA, Lehmann U, McPake B (2018). Health policy and system support to optimise community health worker programmes: an abridged WHO guideline. Lancet Glob Health.

[6] McCollum R, Gomez W, Theobald S, Taegtmeyer M (2016). How equitable are community health worker programmes and which programme features influence equity of community health worker services? A systematic review. BMC Public Health.

[7] Blanchard AK, Prost A, Houweling TAJJ (2019). Effects of community health worker interventions on socioeconomic inequities in maternal and newborn health in low-income and middle-income countries: A mixed-methods systematic review. BMJ Glob Health.

[8] Barnett ML, Gonzalez A, Miranda J, Chavira DA, Lau AS (2018). Mobilizing community health workers to address mental health disparities for underserved populations: A systematic review. Adm Policy Ment Health Ment Health Serv Res.

[9] World Health Organization (2013). Handbook on health inequality monitoring.

[10] O’Neill J, Tabish H, Welch V, Petticrew M, Pottie K, Clarke M (2014). Applying an equity lens to interventions: Using PROGRESS ensures consideration of socially stratifying factors to illuminate inequities in health. J Clin Epidemiol.

[11] Welch V, Petticrew M, Tugwell P, Moher D, O’Neill J, Waters E (2012). PRISMA-equity 2012 extension: Reporting guidelines for systematic reviews with a focus on health equity. PLoS Med.

[12] Welch VA, Petticrew M, O’Neill J, Waters E, Armstrong R, Bhutta ZA (2013). Health equity: Evidence synthesis and knowledge translation methods. Syst Rev.

[13] Belur J, Tompson L, Thornton A, Simon M (2018). Interrater reliability in systematic review methodology: Exploring variation in coder decision-making. Sociol Methods Res.

[14] Higgins JPT, Savović J, Page MJ, Elbers RG, Sterne JAC, Higgins J, Thomas J, Chandler J, Li T, Page M, M C (2019). Assessing risk of bias in a randomized trial. Cochrane handbook for systematic reviews of interventions version 60.

[15] Dixon-Woods M, Sutton AJ, Shaw RL (2007). Appraising qualitative research for inclusion in systematic reviews: A quantitative and qualitative comparison of three methods. J Heal Serv Res Policy.

[16] Pollard TM, Guell C, Morris S. Communal therapeutic mobility in group walking: A meta-ethnography. Soc Sci Med. 262:113241. 10.1016/j.socscimed.2020.113241 32777672

[17] Popay J, Roberts H, Sowden A, Petticrew M, Arai L, Rodgers M, et al. Guidance on the conduct of narrative synthesis in systematic reviews: A product from the ESRC methods programme 2006;(January 2006):1–92.

[18] Borenstein M, Hedges LV, Higgins JPT, Rothstein HR (2011). Introduction to meta-analysis.

[19] Piggot T (2012). Advances in meta-analysis.

[20] Hedges LV, Olkin I (1985). Statistical methods for meta-analysis.

[21] Vilms RJ, McDougal L, Atmavilas Y, Hay K, Triplett DP, Silverman J, et al. Gender inequities in curative and preventive health care use among infants in Bihar, India. J Glob Health. 2017;7(2). 10.7189/jogh.07.020402 PMC5592115 28959437

[22] Geldsetzer P, Vaikath M, De Neve JW, Bossert TJ, Sibandze S, Mkhwanazi M (2017). Distrusting community health workers with confidential health information: A convergent mixed-methods study in Swaziland. Health Policy Plan.

[23] Manu A, Hill Z, Ten Asbroek AH, Soremekun S, Weobong B, Gyan T (2016). Increasing access to care for sick newborns: Evidence from the Ghana Newhints cluster-randomised controlled trial. BMJ Open.

[24] Chudasama RK, Kadri AM, Verma PB, Patel UV, Joshi N, Zalavadiya D (2014). Evaluation of integrated child development services program in Gujarat, India. Indian Pediatr.

[25] Getnet F, Hashi A, Mohamud S, Mowlid H, Klinkenberg E (2017). Low contribution of health extension workers in identification of persons with presumptive pulmonary tuberculosis in Ethiopian Somali region pastoralists. BMC Health Serv Res.

[26] Matovu F, Nanyiti A, Rutebemberwa E (2014). Household health care-seeking costs: Experiences from a randomized, controlled trial of community-based malaria and pneumonia treatment among under-fives in eastern Uganda. Malar J.

[27] Afework MF, Admassu K, Mekonnen A, Hagos S, Asegid M, Ahmed S. Effect of an innovative community based health program on maternal health service utilization in north and south Central Ethiopia: A community based cross sectional study. Reprod Health 2014;11(1):1–9. 10.1186/1742-4755-11-28 PMC4041359 24708848

[28] Agarwal S, Curtis SL, Angeles G, Speizer IS, Singh K, Thomas JC (2019). The impact of India’s accredited social health activist (ASHA) program on the utilization of maternity services: A nationally representative longitudinal modelling study. Hum Resour Health.

[29] Ashenafi A, Karim AM, Ameha A, Erbo A, Getachew N, Betemariam W (2014). Effect of the health extension program and other accessibility factors on care-seeking behaviors for common childhood illnesses in rural Ethiopia. Ethiop Med J.

[30] Shaw B, Amouzou A, Miller NP, Tsui AO, Bryce J, Tafesse M (2015). Determinants of utilization of health extension workers in the context of scale-up of integrated community case management of childhood illnesses in Ethiopia. Am J Trop Med Hyg.

[31] Miyaguchi M, Yasuoka J, Poudyal AK, Silwal RC, Jimba M (2014). Female community health volunteers service utilization for childhood illness- improving quality of health services only is not enough: A cross-sectional study in mid-western region, Nepal. BMC Health Serv Res.

[32] Chami GF, Kontoleon AA, Bulte E, Fenwick A, Kabatereine NB, Tukahebwa EM (2017). Community-directed mass drug administration is undermined by status seeking in friendship networks and inadequate trust in health advice networks. Soc Sci Med.

[33] Yaya Y, Data T, Lindtjørn B (2015). Maternal mortality in rural South Ethiopia: Outcomes of community-based birth registration by health extension workers. PLoS One.

[34] Naidoo N, Railton JP, Khosa SN, Matlakala N, Marincowitz G, McIntyre JA (2018). Fidelity of HIV programme implementation by community health workers in rural Mopani district, South Africa: A community survey. BMC Public Health.

[35] Sakeah E, Doctor HV, McCloskey L, Bernstein J, Yeboah-Antwi K, Mills S (2014). Using the community-based health planning and services program to promote skilled delivery in rural Ghana: Socio-demographic factors that influence women utilization of skilled attendants at birth in northern Ghana. BMC Public Health.

[36] Adongo PB, Phillips JF, Aikins M, Arhin DA, Schmitt M, Nwameme AU (2014). Does the design and implementation of proven innovations for delivering basic primary health care services in rural communities fit the urban setting: The case of Ghana’s Community-based Health Planning and Services (CHPS). Heal Res Policy Syst.

[37] Nwameme AU, Tabong PTN, Adongo PB (2018). Implementing Community-based Health Planning and Services in impoverished urban communities: Health workers’ perspective. BMC Health Serv Res.

[38] Mumtaz Z, Levay A, Bhatti A, Salway S (2015). Good on paper: The gap between programme theory and real-world context in Pakistan’s Community Midwife programme. BJOG An Int J Obstet Gynaecol.

[39] Elazan SJ, Higgins-Steele AE, Fotso JC, Rosenthal MH, Rout D (2016). Reproductive, maternal, newborn, and child health in the community: Task-sharing between male and female health workers in an Indian rural context. Indian J Community Med.

[40] Fotso JC, Higgins-Steele A, Mohanty S (2015). Male engagement as a strategy to improve utilization and community-based delivery of maternal, newborn and child health services: Evidence from an intervention in Odisha, India. BMC Health Serv Res.

[41] Asher L, Hanlon C, Birhane R, Habtamu A, Eaton J, Weiss HA (2018). Community-based rehabilitation intervention for people with schizophrenia in Ethiopia (RISE): A 12 month mixed-methods pilot study. BMC Psychiatry.

[42] Baum A, Mulwafu W, Phiri M, Polack S, Bright T (2019). An intervention to improve uptake of referrals for children with ear disease or hearing loss in Thyolo district, Malawi: Acceptability and feasibility. Int J Environ Res Public Health.

[43] Dusabe-Richards JN, Tesfaye HT, Mekonnen J, Kea A, Theobald S, Datiko DG (2016). Women health extension workers: Capacities, opportunities and challenges to use ehealth to strengthen equitable health systems in southern Ethiopia. Can J Public Heal.

[44] Jackson R, Tesfay FH, Godefay H, Gebrehiwot TG (2016). Health extension workers’ and mothers’ attitudes to maternal health service utilization and acceptance in Adwa Woreda, Tigray region, Ethiopia. PLoS One.

[45] Namukwaya Z, Barlow-Mosha L, Mudiope P, Kekitiinwa A, Matovu JN, Musingye E (2015). Use of peers, community lay persons and Village Health Team (VHT) members improves six-week postnatal clinic (PNC) follow-up and Early Infant HIV Diagnosis (EID) in urban and rural health units in Uganda: A one-year implementation study. BMC Health Serv Res.

[46] Negero MG, Mitike YB, Worku AG, Abota TL. Skilled delivery service utilization and its association with the establishment of Women’s Health Development Army in Yeky district, south West Ethiopia: A multilevel analysis. BMC Res Notes 2018;11(1):1–9. 10.1186/s13104-018-3140-0 PMC5791222 29382372

[47] Hailemariam M, Fekadu A, Medhin G, Prince M, Hanlon C (2019). Equitable access to mental healthcare integrated in primary care for people with severe mental disorders in rural Ethiopia: A community-based cross-sectional study. Int J Ment Heal Syst.

[48] Borg J, Ekman BO, Östergren PO. Is centre-based provision of hearing aids better than community-based provision? A cluster-randomized trial among adolescents in Bangladesh. 2018;13(6):497–503. 10.1080/17483107.2017.1332110 28573939

[49] Fatti G, Jackson D, Goga AE, Shaikh N, Eley B, Nachega JB (2018). The effectiveness and cost-effectiveness of community-based support for adolescents receiving antiretroviral treatment: An operational research study in South Africa. J Int AIDS Soc.

[50] Kawakatsu Y, Tanaka J, Ogawa K, Ogendo K, Honda S (2017). Community unit performance: Factors associated with childhood diarrhea and appropriate treatment in Nyanza Province, Kenya. BMC Public Health.

[51] Kawakatsu Y, Sugishita T, Oruenjo K, Wakhule S, Kibosia K, Were E (2014). Determinants of health facility utilization for childbirth in rural western Kenya: Cross-sectional study. BMC Pregnancy Childbirth.

[52] Karanja S, Gichuki R, Igunza P, Muhula S, Ofware P, Lesiamon J (2018). Factors influencing deliveries at health facilities in a rural Maasai Community in Magadi sub-county, Kenya. BMC Pregnancy Childbirth.

[53] Stollak I, Valdez M, Rivas K, Perry H (2016). Casas maternas in the rural highlands of Guatemala: A mixed-methods case study of the introduction and utilization of birthing facilities by an indigenous population. Glob Heal Sci Pract.

[54] Asiki G, Newton R, Kibirige L, Kamali A, Marions L, Smedman L (2018). Feasibility of using smartphones by village health workers for pregnancy registration and effectiveness of mobile phone text messages on reduction of homebirths in rural Uganda. PLoS One.

[55] Jacobs C, Michelo C, Chola M, Oliphant N, Halwiindi H, Maswenyeho S (2018). Evaluation of a community-based intervention to improve maternal and neonatal health service coverage in the most rural and remote districts of Zambia. PLoS One.

[56] Huq NL, Ahmed A, al Haque N, Hossaine M, Uddin J, Ahmed F (2015). Effect of an integrated maternal health intervention on skilled provider’s care for maternal health in remote rural areas of Bangladesh: A pre and post study. BMC Pregnancy Childbirth.

[57] Shah R, Mullany LC, Darmstadt GL, Talukder RR, Rahman SM, Mannan I (2014). Neonatal mortality risks among preterm births in a rural Bangladeshi cohort. Paediatr Perinat Epidemiol.

[58] Spangler SA, Barry D, Sibley L (2014). An evaluation of equitable access to a community-based maternal and newborn health program in rural Ethiopia. J Midwifery Women’s Heal.

[59] Karim AM, Tamire A, Medhanyie AA, Betemariam W. Changes in equity of maternal, newborn, and child health care practices in 115 districts of rural Ethiopia: Implications for the health extension program. BMC Pregnancy Childbirth. 2015;15(1). 10.1186/s12884-015-0668-z PMC4595284 26438041

[60] Boone P, Eble A, Elbourne D, Frost C, Jayanty C, Lakshminarayana R (2017). Community health promotion and medical provision for neonatal health—CHAMPION cluster randomised trial in Nagarkurnool district, Telangana (formerly Andhra Pradesh), India. PLoS Med.

[61] Ahmed J, Ur Rehman S, Shahab M. Community midwives’ acceptability in their communities: A qualitative study from two provinces of Pakistan. Midwifery. 2017;47(September 2016):53–59. 10.1016/j.midw.2017.02.005 28242494

[62] Give CS, Sidat M, Ormel H, Ndima S, McCollum R, Taegtmeyer M (2015). Exploring competing experiences and expectations of the revitalized community health worker programme in Mozambique: An equity analysis. Hum Resour Health.

[63] Canavati SE, Lawpoolsri S, Quintero CE, Nguon C, Ly P, Pukrittayakamee S (2016). Village malaria worker performance key to the elimination of artemisinin-resistant malaria: A Western Cambodia health system assessment. Malar J.

[64] Loeliger KB, Niccolai LM, Mtungwa LN, Moll A, Shenoi SV (2016). “I have to push him with a wheelbarrow to the clinic”: Community health workers’ roles, needs, and strategies to improve HIV care in rural South Africa. AIDS Patient Care STDs.

[65] Saprii L, Richards E, Kokho P, Theobald S (2015). Community health workers in rural India: Analysing the opportunities and challenges Accredited Social Health Activists (ASHAs) face in realising their multiple roles. Hum Resour Health.

[66] Hailemariam M, Fekadu A, Prince M, Hanlon C (2017). Engaging and staying engaged: A phenomenological study of barriers to equitable access to mental healthcare for people with severe mental disorders in a rural African setting. Int J Equity Health.

[67] Shaw B, Amouzou A, Miller NP, Tafesse M, Bryce J, Surkan PJ (2016). Access to integrated community case management of childhood illnesses services in rural Ethiopia: A qualitative study of the perspectives and experiences of caregivers. Health Policy Plan.

[68] Wester KC, Medhanyie AA, Spigt M, Beumer C, Alemayehu M, Beyene SA (2018). Best practices for addressing socio-cultural barriers to reproductive, maternal and neonatal health service utilization among women from pastoralist communities of Afar, Ethiopia: A qualitative study. Ethiop J Heal Dev.

[69] Ochieng BM, Akunja E, Edwards N, Mombo D, Marende L, Kaseje DCO (2014). Perceptions of health stakeholders on task shifting and motivation of community health workers in different socio demographic contexts in Kenya (nomadic, peri-urban and rural agrarian). BMC Health Serv Res.

[70] Labonté R, Sanders D, Packer C, Schaay N. Is the Alma Ata vision of comprehensive primary health care viable? Findings from an international project. Glob Health Action. 2014;7(1). 10.3402/gha.v7.24997 PMC4141965 25150030

[71] Mambulu-Chikankheni FN, Eyles J, Ditlopo P (2018). Exploring the roles and factors influencing community health workers’ performance in managing and referring severe acute malnutrition cases in two subdistricts in South Africa. Heal Soc Care Community.

[72] Kea AZ, Tulloch O, Datiko DG, Theobald S, Kok MC (2018). Exploring barriers to the use of formal maternal health services and priority areas for action in Sidama zone, southern Ethiopia. BMC Pregnancy Childbirth.

[73] Gupta M, Bosma H, Angeli F, Kaur M, Chakrapani V, Rana M (2017). Impact of a multi-strategy community intervention to reduce maternal and child health inequalities in India: A qualitative study in Haryana. PLoS One.

[74] Shah R, Rehfuess EA, Paudel D, Maskey MK, Delius M (2018). Barriers and facilitators to institutional delivery in rural areas of Chitwan district, Nepal: A qualitative study. Reprod Health.

[75] Panday S, Bissell P, Van Teijlingen E, Simkhada P (2017). The contribution of female community health volunteers (FCHVs) to maternity care in Nepal: A qualitative study. BMC Health Serv Res.

[76] Angeles G, Ahsan KZ, Streatfield PK, El Arifeen S, Jamil K (2019). Reducing inequity in urban health: Have the intra-urban differentials in reproductive health service utilization and child nutritional outcome narrowed in Bangladesh?. J Urban Health.

[77] Flink IJE, Ziebe R, Vagaï D, Van De Looij F, Van ‘T Riet H, Houweling TAJ (2016). Targeting the poorest in a performance-based financing programme in northern Cameroon. Health Policy Plan.

[78] Liverani M, Nguon C, Sok R, Kim D, Nou P, Nguon S (2017). Improving access to health care amongst vulnerable populations: A qualitative study of village malaria workers in Kampot, Cambodia. BMC Health Serv Res.

[79] Jefferds MED, Mirkovic KR, Subedi GR, Mebrahtu S, Dahal P, Perrine CG (2015). Predictors of micronutrient powder sachet coverage in Nepal. Matern Child Nutr.

[80] Kosec K, Avula R, Holtemeyer B, Tyagi P, Hausladen S, Menon P (2015). Predictors of essential health and nutrition service delivery in Bihar, India: Results from household and frontline worker surveys. Glob Heal Sci Pr.

[81] Cros M, Cavagnero E, Alfred JP, Sjoblom M, Collin N, Mathurin T. Equitable realization of the right to health in Haiti: How household data inform health seeking behavior and financial risk protection. Int J Equity Health. 2019;18(1). 10.1186/s12939-019-0973-7 PMC6537186 31133035

[82] Bâ EH, Pitt C, Dial Y, Faye SL, Cairns M, Faye E (2018). Implementation, coverage and equity of large-scale door-to-door delivery of Seasonal Malaria Chemoprevention (SMC) to children under 10 in Senegal. Sci Rep.

[83] Yitayal M, Berhane Y, Worku A, Kebede Y. Health extension program factors, frequency of household visits and being model households, improved utilization of basic health services in Ethiopia. BMC Health Serv Res. 2014;14. 10.1186/1472-6963-14-156 PMC4234278 24708661

[84] Teklehaimanot HD, Teklehaimanot A, Yohannes M, Biratu D (2016). Factors influencing the uptake of voluntary HIV counseling and testing in rural Ethiopia: A cross sectional study. BMC Public Health.

[85] Edmond KM, Foshanji AI, Naziri M, Higgins-Steele A, Burke JM, Strobel N (2019). Conditional cash transfers to improve use of health facilities by mothers and newborns in conflict affected countries, a prospective population based intervention study from Afghanistan. BMC Pregnancy Childbirth.

[86] Corbin JH, Mittelmark MB, Lie GT (2016). Grassroots volunteers in context: Rewarding and adverse experiences of local women working on HIV and AIDS in Kilimanjaro, Tanzania. Glob Health Promot.

[87] Kimani-Murage EW, Griffiths PL, Wekesah FM, Wanjohi M, Muhia N, Muriuki P (2017). Effectiveness of home-based nutritional counselling and support on exclusive breastfeeding in urban poor settings in Nairobi: A cluster randomized controlled trial. Glob Health.

[88] Vellakkal S, Gupta A, Khan Z, Stuckler D, Reeves A, Ebrahim S (2017). Has India’s national rural health mission reduced inequities in maternal health services? A pre-post repeated cross-sectional study. Health Policy Plan.

[89] Sibley LM, Amare Y, Abebe ST, Belew ML, Shiffra K, Barry D (2017). Appropriateness and timeliness of care-seeking for complications of pregnancy and childbirth in rural Ethiopia: A case study of the maternal and newborn health in Ethiopia partnership. J Health Popul Nutr.

[90] Sharkey AB, Martin S, Cerveau T, Wetzler E, Berzal R. Demand generation and social mobilisation for integrated community case management (iCCM) and child health: Lessons learned from successful programmes in Niger and Mozambique. J Glob Health. 2014;4(2). 10.7189/jogh.04.020410 PMC4267098 25520800

[91] Soremekun S, Kasteng F, Lingam R, Vassall A, Kertho E, Settumba S (2018). Variation in the quality and out-of-pocket cost of treatment for childhood malaria, diarrhoea, and pneumonia: Community and facility based care in rural Uganda. PLoS One.

[92] Gope RK, Tripathy P, Prasad V, Pradhan H, Sinha RK, Panda R (2019). Effects of participatory learning and action with women’s groups, counselling through home visits and crèches on undernutrition among children under three years in eastern India: A quasi-experimental study. BMC Public Health.

[93] Jolly SP, Rahman M, Afsana K, Yunus FM, Chowdhury AM (2016). Evaluation of maternal health service indicators in urban slum of Bangladesh. PLoS One.

[94] Avery LS, Du Plessis E, Shaw SY, Sankaran D, Njoroge P, Kayima R (2017). Enhancing the capacity and effectiveness of community health volunteers to improve maternal, newborn and child health: Experience from Kenya. Can J Public Heal.

[95] Ekirapa-Kiracho E, Kananura RM, Tetui M, Namazzi G, Mutebi A, George A (2017). Effect of a participatory multisectoral maternal and newborn intervention on maternal health service utilization and newborn care practices: A quasi-experimental study in three rural Ugandan districts. Glob Health Action.

[96] Seth A, Tomar S, Singh K, Chandurkar D, Chakraverty A, Dey A (2017). Differential effects of community health worker visits across social and economic groups in Uttar Pradesh, India: A link between social inequities and health disparities. Int J Equity Health.

[97] Adams AM, Nababan HY, Hanifi SM, Manzoor Ahmed Hanifi SM (2015). Building social networks for maternal and newborn health in poor urban settlements: A cross-sectional study in Bangladesh. PLoS One.

[98] Gonzalez-Casanova I, Nguyen PH, Young MF, Harding KB, Reinhart G, Nguyen H (2017). Predictors of adherence to micronutrient supplementation before and during pregnancy in Vietnam. BMC Public Health.

[99] Johri M, Chandra D, Koné GK, Dudeja S, Sylvestre MP, Sharma JK (2015). Interventions to increase immunisation coverage among children 12-23 months of age in India through participatory learning and community engagement: Pilot study for a cluster randomised trial. BMJ Open.

[100] McDougal L, Atmavilas Y, Hay K, Silverman JG, Tarigopula UK, Raj A (2017). Making the continuum of care work for mothers and infants: Does gender equity matter? Findings from a quasi-experimental study in Bihar, India. PLoS One.

[101] Kawakatsu Y, Tanaka J, Ogawa K, Ogendo K, Honda S (2015). Effects of three interventions and determinants of full vaccination among children aged 12-59 months in Nyanza province, Kenya. Public Health.

[102] Nair N, Tripathy P, Sachdev HS, Pradhan H, Bhattacharyya S, Gope R (2017). Effect of participatory women’s groups and counselling through home visits on children’s linear growth in rural eastern India (CARING trial): A cluster-randomised controlled trial. Lancet Glob Health.

[103] Ijumba P, Doherty T, Jackson D, Tomlinson M, Sanders D, Swanevelder S (2015). Effect of an integrated community-based package for maternal and newborn care on feeding patterns during the first 12 weeks of life: A cluster-randomized trial in a south African township. Public Health Nutr.

[104] Kimani-Murage EW, Norris SA, Mutua MK, Wekesah F, Wanjohi M, Muhia N (2016). Potential effectiveness of community health strategy to promote exclusive breastfeeding in urban poor settings in Nairobi, Kenya: A quasi-experimental study. J Dev Orig Health Dis.

[105] Gudu W, Addo B (2017). Factors associated with utilization of skilled service delivery among women in rural northern Ghana: A cross sectional study. BMC Pregnancy Childbirth.

[106] Muhumuza Kananura R, Tetui M, Bua J, Ekirapa-Kiracho E, Mutebi A, Namazzi G (2017). Effect of a participatory multisectoral maternal and newborn intervention on birth preparedness and knowledge of maternal and newborn danger signs among women in eastern Uganda: A quasi-experiment study. Glob Health Action.

[107] Mitra DK, Mullany LC, Harrison M, Mannan I, Shah R, Begum N (2018). Incidence and risk factors of neonatal infections in a rural Bangladeshi population: A community-based prospective study. J Health Popul Nutr.

[108] Peltzer K, Babayigit S, Rodriguez VJ, Jean J, Sifunda S, Jones DL (2018). Effect of a multicomponent behavioural PMTCT cluster randomised controlled trial on HIV stigma reduction among perinatal HIV positive women in Mpumalanga province, South Africa. SAHARA J J Soc Asp HIV/AIDS Res Alliance.

[109] Wagner AL, Xia L, Ghosh A, Datta S, Pandey P, Santra S (2018). Using community health workers to refer pregnant women and young children to health care facilities in rural West Bengal, India: A prospective cohort study. PLoS One.

[110] Lee HY, Oh J, Heo J, Abraha A, Perkins JM, Lee JK, et al. Association between maternal literacy and child vaccination in Ethiopia and southeastern India and the moderating role of health workers: A multilevel regression analysis of the Young Lives study. Glob Health Action. 2019;12(1). 10.1080/16549716.2019.1581467 PMC6461100 30957685

[111] Akeju DO, Oladapo OT, Vidler M, Akinmade AA, Sawchuck D, Qureshi R (2016). Determinants of health care seeking behaviour during pregnancy in Ogun state, Nigeria. Reprod Health.

[112] Puett C, Alderman H, Sadler K, Coates J (2015). “Sometimes they fail to keep their faith in us”: Community health worker perceptions of structural barriers to quality of care and community utilisation of services in Bangladesh. Matern Child Nutr.

[113] Beam M, Spencer A, Fernandez L, Atto R, Muro C, Vilchez P (2018). Barriers to participation in a community-based program to control transmission of Taenia solium in Peru. Am J Trop Med Hyg.

[114] Ayon S, Ndimbii J, Jeneby F, Abdulrahman T, Mlewa O, Wang B (2018). Barriers and facilitators of access to HIV, harm reduction and sexual and reproductive health services by women who inject drugs: Role of community-based outreach and drop-in centers. AIDS Care.

[115] Khuzwayo L, Moshabela M. Benefits of health reform for households in rural South Africa following implementation of ward-based primary healthcare outreach teams: A qualitative inquiry. Glob Health Action. 2018;11(1). 10.1080/16549716.2018.1527666 PMC6197010 30326822

[116] Bello G, Faragher B, Sanudi L, Namakhoma I, Banda H, Malmborg R (2017). The effect of engaging unpaid informal providers on case detection and treatment initiation rates for TB and HIV in rural Malawi (triage plus): A cluster randomised health system intervention trial. PLoS One.

[117] Geldsetzer P, Vaikath M, De Neve JW, Bossert TJ, Sibandze S, Bärnighausen T (2017). Household coverage of Swaziland’s national community health worker programme: A cross-sectional population-based study. Trop Med Int Health.

[118] Shaw B, Amouzou A, Miller NP, Bryce J, Surkan PJ (2017). A qualitative exploration of care-seeking pathways for sick children in the rural Oromia region of Ethiopia. BMC Health Serv Res.

[119] Linn NYY, Kathirvel S, Das M, Thapa B, Rahman MM, Maung TM (2018). Are village health volunteers as good as basic health staffs in providing malaria care? A country wide analysis from Myanmar, 2015. Malar J.

[120] Nair N, Tripathy P, Sachdev HS, Pradhan H, Bhattacharyya S, Gope R (2017). Effect of participatory women’s groups and counselling through home visits on children’s linear growth in rural eastern India (CARING trial): A cluster-randomised controlled trial. Lancet Glob Health.

[121] Yirgu R, Molla M, Sibley L (2017). Determinants of neonatal mortality in rural northern Ethiopia: A population based nested case control study. PLoS One.

[122] Shidhaye R, Murhar V, Gangale S, Aldridge L, Shastri R, Parikh R (2017). The effect of VISHRAM, a grass-roots community-based mental health programme, on the treatment gap for depression in rural communities in India: A population-based study. Lancet Psychiatry.

[123] Coker M, Etiebet M-A, Chang H, Awwal G, Jumare J, Musa B (2015). Socio-demographic and adherence factors associated with viral load suppression in HIV-infected adults initiating therapy in northern Nigeria: A randomized controlled trial of a peer support intervention. Curr HIV Res.

[124] Gill CJ, MacLeod WB, Phiri-Mazala G, Guerina NG, Mirochnick M, Knapp AB (2014). Can traditional birth attendants be trained to accurately identify septic infants, initiate antibiotics, and refer in a rural African setting?. Glob Heal Sci Pract.

[125] Lusli M, Peters R, van Brakel W, Zweekhorst M, Iancu S, Bunders J (2016). The impact of a rights-based counselling intervention to reduce stigma in people affected by leprosy in Indonesia. PLoS Negl Trop Dis.

[126] Jackson R, Kilsby D, Hailemariam A (2019). Gender exploitative and gender transformative aspects of employing health extension workers under Ethiopia’s health extension program. Trop Med Int Health.

[127] Najafizada SAM, Bourgeault IL, Labonté R (2019). A gender analysis of a national community health workers program: A case study of Afghanistan. Glob Public Health.

[128] Steege R, Taegtmeyer M, McCollum R, Hawkins K, Ormel H, Kok M (2018). How do gender relations affect the working lives of close to community health service providers? Empirical research, a review and conceptual framework. Soc Sci Med.

[129] Panday S, Bissell P, Van Teijlingen E, Simkhada P (2019). Perceived barriers to accessing Female Community Health Volunteers’ (FCHV) services among ethnic minority women in Nepal: A qualitative study. PLoS One.

[130] Wharton-Smith A, Rassi C, Batisso E, Ortu G, King R, Endriyas M (2019). Gender-related factors affecting health seeking for neglected tropical diseases: Findings from a qualitative study in Ethiopia. PLoS Negl Trop Dis.

[131] Farmer DB, Berman L, Ryan G, Habumugisha L, Basinga P, Nutt C (2015). Motivations and constraints to family planning: A qualitative study in Rwanda’s southern Kayonza District. Glob Heal Sci Pract.

[132] Gittings L (2016). ‘When you visit a man you should prepare yourself’: Male community care worker approaches to working with HIV-positive male clients in Cape Town, South Africa. Cult Health Sex.

[133] Musoke D, Ssemugabo C, Ndejjo R, Ekirapa-Kiracho E, George AS (2018). Reflecting strategic and conforming gendered experiences of community health workers using photovoice in rural Wakiso district. Uganda Hum Resour Health.

[134] Jacobs C, Michelo C, Moshabela M (2018). Implementation of a community-based intervention in the most rural and remote districts of Zambia: A process evaluation of safe motherhood action groups. Implement Sci.

[135] Feldhaus I, Silverman M, Lefevre AE, Mpembeni R, Mosha I, Chitama D (2015). Equally able, but unequally accepted: Gender differentials and experiences of community health volunteers promoting maternal, newborn, and child health in Morogoro region, Tanzania. Int J Equity Health.

[136] Datiko DG, Yassin MA, Tulloch O, Asnake G, Tesema T, Jamal H (2015). Exploring providers’ perspectives of a community based TB approach in southern Ethiopia: Implication for community based approaches. BMC Health Serv Res.

[137] Tancred T, Mandu R, Hanson C, Okuga M, Manzi F, Peterson S (2018). How people-centred health systems can reach the grassroots: Experiences implementing community-level quality improvement in rural Tanzania and Uganda. Health Policy Plan.

[138] Rafiq MY, Wheatley H, Mushi HP, Baynes C (2019). Who are CHWs? An ethnographic study of the multiple identities of community health workers in three rural districts in Tanzania. BMC Health Serv Res.

[139] Tesfaye S, Barry D, Gobezayehu AG, Frew AH, Stover KE, Tessema H (2014). Improving coverage of postnatal care in rural Ethiopia using a community-based, collaborative quality improvement approach. J Midwifery Womens Heal.

[140] Juma PA, Mutombo N, Mukiira C (2015). Women’s attitudes towards receiving family planning services from community health workers in rural Western Kenya. Afr Health Sci.

[141] Negussie A, Girma G (2017). Is the role of health extension workers in the delivery of maternal and child health care services a significant attribute? The case of dale district, southern Ethiopia. BMC Health Serv Res.

[142] Russell CL, Sallau A, Emukah E, Graves PM, Noland GS, Ngondi JM (2015). Determinants of bed net use in Southeast Nigeria following mass distribution of LLINs: Implications for social behavior change interventions. PLoS One.

[143] Ara G, Khanam M, Papri N, Nahar B, Haque MA, Kabir I (2018). Peer counselling improves breastfeeding practices: A cluster randomized controlled trial in urban Bangladesh. Matern Child Nutr.

[144] Barry D, Frew AH, Mohammed H, Desta BF, Tadesse L, Aklilu Y (2014). The effect of community maternal and newborn health family meetings on type of birth attendant and completeness of maternal and newborn care received during birth and the early postnatal period in rural Ethiopia. J Midwifery Womens Heal.

[145] Choulagai BP, Onta S, Subedi N, Bhatta DN, Shrestha B, Petzold M (2017). A cluster-randomized evaluation of an intervention to increase skilled birth attendant utilization in mid- and far-western Nepal. Health Policy Plan.

[146] Darega B, Dida N, Tafese F, Ololo S (2016). Institutional delivery and postnatal care services utilizations in Abuna Gindeberet District, west Shewa, Oromiya region, Central Ethiopia: A community-based cross sectional study. BMC Pregnancy Childbirth.

[147] George AS, Mohan D, Gupta J, Lefevre AE, Balakrishnan S, Ved R (2018). Can community action improve equity for maternal health and how does it do so? Research findings from Gujarat, India. Int J Equity Health.

[148] Sam-Agudu NA, Ramadhani HO, Isah C, Anaba U, Erekaha S, Fan-Osuala C (2017). The impact of structured Mentor mother programs on 6-month postpartum retention and viral suppression among HIV-positive women in rural Nigeria: A prospective paired cohort study. J Acquir Immune Defic Syndr.

[149] Burke HM, Chen M, Buluzi M, Fuchs R, Wevill S, Venkatasubramanian L (2019). Factors affecting continued use of subcutaneous depot medroxyprogesterone acetate (DMPA-SC): A secondary analysis of a 1-year randomized trial in Malawi. Glob Heal Sci Pract.

[150] Brooks MI, Johns NE, Quinn AK, Boyce SC, Fatouma IA, Oumarou AO (2019). Can community health workers increase modern contraceptive use among young married women? A cross-sectional study in rural Niger. Reprod Health.

[151] Taleb F, Perkins J, Ali NA, Capello C, Ali M, Santarelli C (2015). Transforming maternal and newborn health social norms and practices to increase utilization of health services in rural Bangladesh: A qualitative review. BMC Pregnancy Childbirth.

[152] Balakrishnan R, Gopichandran V, Chaturvedi S, Chatterjee R, Mahapatra T, Chaudhuri I (2016). Continuum of Care Services for Maternal and Child Health using mobile technology - a health system strengthening strategy in low and middle income countries. BMC Med Inform Decis Mak.

[153] Matsumoto-Takahashi ELA, Tongol-Rivera P, Villacorte EA, Angluben RU, Yasuoka J, Kano S (2014). Determining the impact of community awareness-raising activities on the prevention of malaria transmission in Palawan, the Philippines. Parasitol Int.

[154] Pelcastre-Villafuerte B, Ruiz M, Meneses S, Amaya C, Márquez M, Taboada A (2014). Community-based health care for indigenous women in Mexico: A qualitative evaluation. Int J Equity Health.

[155] Bergen N, Mamo A, Asfaw S, Abebe L, Kurji J, Kiros G (2018). Perceptions and experiences related to health and health inequality among rural communities in Jimma zone, Ethiopia: A rapid qualitative assessment. Int J Equity Health.

[156] McCollum R, Otiso L, Mireku M, Theobald S, De Koning K, Hussein S (2016). Exploring perceptions of community health policy in Kenya and identifying implications for policy change. Health Policy Plan.

[157] Kelbessa Z, Baraki N, Egata G (2014). Level of health extension service utilization and associated factors among community in Abuna Gindeberet District, west Shoa zone, Oromia regional state, Ethiopia. BMC Health Serv Res.

[158] Luckow PW, Kenny A, White E, Dorr L, Erlandson K, Grant B (2020). Implementation research on health services in rural Liberia.

[159] Buchner DL, Brenner JL, Kabakyenga J, Teddy K, Maling S, Barigye C, et al. Stakeholders’ perceptions of integrated community case management by community health workers: A post-intervention qualitative study. PLoS One. 2014;9(6). 10.1371/journal.pone.0098610 PMC4057118 24927074

[160] Tschirhart N, Nosten F, Foster AM (2017). Migrant tuberculosis patient needs and health system response along the Thailand-Myanmar border. Health Policy Plan.

[161] Naidoo S, Naidoo D, Govender P. Community healthcare worker response to childhood disorders: Inadequacies and needs. African J Prim Heal Care Fam Med 2019;11(1):1–10. 10.4102/phcfm.v11i1.1871 PMC6556913 31038346

[162] Crenshaw K (1991). Mapping the margins: Intersectionality, identity politics, and violence against women of color. Stanford Law Rev.

[163] Tefera W, Tesfaye H, Bekele A, Kayessa E, Waltensperger KZ, Marsh DR (2014). Factors influencing the low utilization of curative child health services in Shebedino District, Sidama zone, Ethiopia. Ethiop Med J.

[164] Nandi S, Schneider H (2014). Addressing the social determinants of health: A case study from the Mitanin (community health worker) programme in India. Health Policy Plan.

[165] de Lange N, Mitchell C (2016). Community health workers as cultural producers in addressing gender-based violence in rural South Africa. Glob Public Health.

[166] Woods-Jaeger BA, Kava CM, Akiba CF, Lucid L, Dorsey S (2017). The art and skill of delivering culturally responsive trauma-focused cognitive behavioral therapy in Tanzania and Kenya. Psychol Trauma Theory Res Pract Policy.

[167] Miller JS, Musominali S, Baganizi M, Paccione GA (2014). A process evaluation of performance-based incentives for village health workers in Kisoro district, Uganda. Hum Resour Health.

[168] Edward A, Branchini C, Aitken I, Roach M, Osei-Bonsu K, Arwal SH (2015). Toward universal coverage in Afghanistan: A multi-stakeholder assessment of capacity investments in the community health worker system. Soc Sci Med.

[169] Sharma R, Webster P, Bhattacharyya S. Factors affecting the performance of community health workers in India: A multi-stakeholder perspective. Glob Health Action. 2014;7(1). 10.3402/gha.v7.25352 PMC4197397 25319596

[170] Kok MC, Namakhoma I, Nyirenda L, Chikaphupha K, Broerse JEW, Dieleman M (2016). Health surveillance assistants as intermediates between the community and health sector in Malawi: Exploring how relationships influence performance. BMC Health Serv Res.

[171] Kwon HJ, Ramasamy R, Morgan A (2014). “How often? How much? Where from?” knowledge, attitudes, and practices of mothers and health workers to iron supplementation program for children under five in rural Tamil Nadu, South India. Asia-Pacific J Public Heal.

[172] Kane S, Radkar A, Gadgil M, McPake B. Community health workers as influential health system actors and not “just another pair of hands”. Int J Health Policy Manag. 2020;(x):1–10. 10.34172/ijhpm.2020.58 PMC9056200 32610755

[173] Olaniran A, Smith H, Unkels R, Bar-Zeev S, van den Broek N. Who is a community health worker? - a systematic review of definitions. Glob Health Action. 2017;10(1). 10.1080/16549716.2017.1272223 PMC5328349 28222653

[174] Kane S, Kok M, Ormel H, Otiso L, Sidat M, Namakhoma I (2016). Limits and opportunities to community health worker empowerment : A multi-country comparative study. Soc Sci Med.

[175] Ved R, Scott K, Gupta G, Ummer O, Singh S, Srivastava A (2019). How are gender inequalities facing India’s one million ASHAs being addressed? Policy origins and adaptations for the world’s largest all-female community health worker programme. Hum Resour Health.

[176] Hampshire K, Porter G, Mariwah S, Munthali A, Robson E, Owusu SA (2017). Who bears the cost of “informal mhealth”? Health-workers’ mobile phone practices and associated political-moral economies of care in Ghana and Malawi. Health Policy Plan.

[177] Maes K (2014). “Volunteers are not paid because they are priceless”: Community health worker capacities and values in an AIDS treatment intervention in urban Ethiopia. Med Anthropol Q.

[178] Maes K, Kohrt BA, Closser S (2010). Culture, status and context in community health worker pay: Pitfalls and opportunities for policy research. A commentary on Glenton et al. (2010). Soc Sci Med.

[179] Maes K (2010). Examining health-care volunteerism in a food- and financially-insecure world. Bull World Health Organ.

[180] Maes K, Kohrt BA, Mendenhall E (2015). Task-shifting in global health: mental health implications for community health workers and volunteers. Global mental health: anthropological perspectives.

[181] Maes K (2012). Volunteerism or labor exploitation? Harnessing the volunteer spirit to sustain AIDS treatment programs in urban Ethiopia. Hum Organ.

[182] Chase LE, Gurung D, Shrestha P, Rumba S (2020). Gendering psychosocial care: Risks and opportunities for global mental health. Lancet Psychiatry.

[183] Bhatia K (2014). Community health worker programs in India: A rights-based review. Perspect Public Health.

[184] World Health Organization (2016). Global strategy on human resources for health: Workforce 2030.

[185] World Health Organization (2010). A conceptual framework for action on the social determinants of health.

[186] Gilmore B, Adams BJ, Bartoloni A, Alhaydar B, McAuliffe E, Raven J (2016). Improving the performance of community health workers in humanitarian emergencies: A realist evaluation protocol for the PIECES programme. BMJ Open.

[187] Devkota HR, Clarke A, Murray E, Groce N (2017). Do experiences and perceptions about quality of care differ among social groups in Nepal? : A study of maternal healthcare experiences of women with and without disabilities, and Dalit and non-Dalit women. Abe T, editor. PLoS One.

[188] Cáceres C, Pecheny M, Frasca T, Rios RR, Pocahy F (2008). Review of legal frameworks and the situation of human rights related to sexual diversity in low and middle income countries.

[189] Swartz L, Kilian S, Twesigye J, Attah D, Chiliza B (2014). Language, culture, and task shifting - an emerging challenge for global mental health. Glob Health Action.

[190] Sarin E, Lunsford SS (2017). How female community health workers navigate work challenges and why there are still gaps in their performance: A look at female community health workers in maternal and child health in two Indian districts through a reciprocal determinism framework. Hum Resour Health.

[191] Maes K, Closser S, Kalofonos I (2014). Listening to community health workers: How ethnographic research can inform positive relationships among community health workers, health institutions, communities. Am J Public Health.

[192] Dixon-Woods M, Bonas S, Booth A, Jones DR, Miller T, Sutton AJ (2006). How can systematic reviews incorporate qualitative research? A critical perspective. Qual Res.

[193] Dixon-Woods M, Shaw RL, Agarwal S, Smith JA (2004). The problem of appraising qualitative research. Qual Saf Heal Care.

[194] Toye F, Seers K, Allcock N, Briggs M, Carr E, Andrews J (2013). “Trying to pin down jelly” - exploring intuitive processes in quality assessment for meta-ethnography. BMC Med Res Methodol.

[195] Toye F, Seers K, Allcock N, Briggs M, Carr E, Barker K. Meta-ethnography 25 years on: Challenges and insights for synthesising a large number of qualitative studies. BMC Med Res Methodol. 2014;14(80). 10.1186/1471-2288-14-80 PMC4127190 24951054

