# Supplementary material for: Community health workers and health equity in low- and middle-income countries: systematic review and recommendations for policy and practice
Source: Int J Equity Health. 2022 Apr 11;21:49. doi: 10.1186/s12939-021-01615-y (PMC8996551; doi:10.1186/s12939-021-01615-y)
Supplement: Supplementary file 1 — Additional file 1. Search Strategy: provides an example of the search strategy used in one database. [file 12939_2021_1615_MOESM1_ESM.docx]

# Additional File 1: Search Strategy

This search strategy was modified from an earlier systematic review on the equitability of CHW programmes (McCollum *et al.* 2016). For the present review, additional equity terms were added (‘caste’ and ‘ethnicity’) to capture articles covering the broadest possible range of equity stratifiers. An example of the search strategy used in one database is included below.

**Scopus Search**

| Filters   - Year 2014-present - English language - Abstract - Publication type: article or review   Search #1 and #2 and #3 |
| --- |

COMMUNITY HEALTH WORKER TERMS

| #1 | "community health worker" OR "community health workers" OR "community health aides" OR "community health aide" OR "family planning personnel" OR "village health worker" OR "village health workers" OR "home health aide" OR "home health aides" OR "allied health personnel" OR "population program specialists" OR "nurses aides" OR "nurses aide" OR "nursing auxiliaries" OR "nursing auxiliary" OR "volunteerism" OR "non professional home care" OR "nonprofessional home care" OR "peer group" OR "social support" OR "psychosocial networks" OR "social networks" OR "lay worker" OR "lay workers" OR "lay visitors" OR "lay attendants" OR "lay support" OR "lay person" OR "lay personnel" OR "lay helpers" OR "lay carer" OR "lay carers" OR "lay caregiver" OR "lay caregivers" OR "lay staff" OR "lay midwife" OR "lay midwives" OR "lay providers" OR "lay health worker" OR "lay health workers" OR "lay counsellor" OR "lay counsellors" OR "lay volunteer" OR "lay volunteers" OR "lay mentor" OR "lay mentors" OR "voluntary workers" OR "voluntary worker" OR "voluntary visitor" OR "voluntary visitors" OR "voluntary support" OR "voluntary supporter" OR "voluntary supporters" OR "volunteer worker" OR "volunteer workers" OR "volunteer support" OR "volunteer supporters" OR "volunteer supporter" OR "volunteer caregivers" OR "volunteer caregiver" OR "volunteer staff" OR "volunteer providers" OR "volunteer provider" OR "volunteer care givers" OR "volunteer practitioners" OR "volunteer care" OR "volunteer nursing" OR "informal workers" OR "informal visitor" OR "informal visitors" OR "informal support" OR "informal supporter" OR "informal supporters" OR "informal helpers" OR "informal carer" OR "informal carers" OR "informal caregiver" OR "informal caregivers" OR "informal providers" OR "informal care givers" OR "informal practitioners" OR "untrained workers" OR "untrained attendants" OR "untrained person" OR "untrained personnel" OR "untrained staff" OR "untrained midwives" OR "untrained providers" OR "untrained practitioners" OR "Unlicensed visitor" OR "unlicensed visitors" OR "unlicensed support" OR "unlicensed supporters" OR "unlicensed supporter" OR "unlicensed personnel" OR "unlicensed caregivers" OR "unlicensed staff" OR "unlicensed providers" OR "unlicensed practitioners" OR "nonprofessional workers" OR "non professional workers" OR "non professional support" OR "nonprofessional supporter" OR "nonprofessional supporters" OR "nonprofessional personnel" OR "non professional personnel" OR "non professional carers" OR "nonprofessional caregivers" OR "non professional caregivers" OR "nonprofessional staff" OR "non professional staff" OR "non professional visitor" OR "non professional visitors" OR "non professional providers" OR Paraprofessional* OR paramedic OR paramedics OR "paramedical worker" OR "paramedical workers" OR "paramedical personnel" OR "allied health worker" OR "allied health workers" OR "support worker" OR "support workers" OR "Trained volunteer" OR "trained volunteers" OR "trained health worker" OR "trained health workers" OR "trained mothers" OR "trained health care workers" OR "trained healthcare workers" OR "community health care workers" OR "community healthcare workers" OR "community distributors" OR "community based health workers" OR "community based distributors" OR "village healthcare workers" OR "village health workers" OR "village health care workers" OR "community based providers" OR "community workers" OR "community based workers" OR "frontline health workers" OR "frontline health worker" OR "frontline workers" OR "frontline worker" OR "community volunteer" OR "community volunteers" OR "community based volunteers" OR "community support" OR "community based support" OR "birth attendant" OR "birth attendants" OR "birth assistants" OR Doula* OR douladural* OR Monitrice* OR "peer volunteer" OR "peer volunteers" OR "peer mentor" OR "peer mentors" OR "peer support" OR "peer intervention" OR "peer interventions" OR "peer counsellor" OR "peer counsellors" OR "church based intervention" OR "church based interventions" OR "church based program" OR Linkworker OR linkworkers OR "link worker" OR "link workers" OR "barefoot doctor" OR "barefoot doctors" OR Outreach OR "home care" OR "home aide" OR "home aides" OR "home nursing" OR "home support" OR "home intervention" OR "home interventions" OR "home treatment" OR "home treatments" OR "home visitor" OR "home visitors" OR "expert patient" OR "expert patients" OR "health promoter" OR "health promoters" OR "health extension worker" OR "health extension workers" OR "mentor mother" OR "mentor mothers" |
| --- | --- |

EQUITY TERMS

| #2 | "rural population" OR "rural populations" OR "rural community" OR "rural communities" OR "rural spatial distribution" OR "healthcare disparities" OR "healthcare disparity" OR "health care disparity" OR "health care disparities" OR Disadvantage* OR "vulnerable population" OR "vulnerable populations" OR "underserved patients" OR "underserved population" OR "underserved populations" OR "sensitive population groups" OR "sensitive population group" OR "sensitive populations" OR "sensitive population" OR "medically underserved area" OR "physician shortage area" OR Inequalit* OR "socioeconomic factors" OR "socioeconomic factor" OR "low income population" OR "low income populations" OR "standard of living" OR "gender identity" or Gender OR "sex role" OR "woman role" OR "women role" OR "man role" OR "men role" OR "gender role" OR equit* OR inequit* or disparit* OR equalit* OR disabilit* OR caste OR ethnicity |
| --- | --- |

OUTCOME TERMS

| #3 | "health services accessibility" OR "access to health care" OR "accessibility of health services" OR "health services geographic accessibility" OR "contraceptive availability" OR "program accessibility" OR "program availability" OR "availability of health services" OR "health services availability" OR "acceptability of health care" OR "acceptability of healthcare" OR "patient acceptance of health care" OR "patient acceptance of healthcare" OR "program acceptability" OR "health care seeking behaviour" OR "health seeking behaviour" OR "patient acceptance of healthcare" OR "acceptors" OR "acceptor characteristics" OR "community empowerment" OR "utilization" OR "coverage" OR "community governance" OR "quality of healthcare" OR "quality of health care" OR "healthcare quality" OR "health care quality" |
| --- | --- |
