# Supplementary material for: Community health workers and health equity in low- and middle-income countries: systematic review and recommendations for policy and practice
Source: Int J Equity Health. 2022 Apr 11;21:49. doi: 10.1186/s12939-021-01615-y (PMC8996551; doi:10.1186/s12939-021-01615-y)
Supplement: Supplementary file 2 — Additional file 2. Qualitative Synthesis Coding Framework: provides the coding framework used in thematic content analysis of qualitative evidence. [file 12939_2021_1615_MOESM2_ESM.docx]

**Additional File 2: Qualitative Synthesis Coding Framework**

**I. Equitability of CHW interventions & Influencing Factors**

1. Place of Residence:
   1. Barriers to equitability
   2. Facilitators of equitability
2. Race/Ethnicity/Culture/Language PLUS Caste:
   1. Barriers to equitability
   2. Facilitators of equitability
3. Occupation:
   1. Barriers to equitability
   2. Facilitators of equitability
4. Gender:
   1. Barriers to equitability
   2. Facilitators of equitability
5. Religion:
   1. Barriers to equitability
   2. Facilitators of equitability
6. Education:
   1. Barriers to equitability
   2. Facilitators of equitability
7. Socioeconomic Status:
   1. Barriers to equitability
   2. Facilitators of equitability
8. Social Capital:
   1. Barriers to equitability
   2. Facilitators of equitability
9. Age:
   1. Barriers to equitability
   2. Facilitators of equitability
10. Sexual orientation:
    1. Barriers to equitability
    2. Facilitators of equitability
11. Disability:
    1. Barriers to equitability
    2. Facilitators of equitability
12. Intersectional barriers (any discussion of how multiple axes of marginalization interact to prevent equitable service provision)
13. General perceptions of equitability (general comments, opinions, or views on equitability of CHW intervention/programme that are not specific to any of the above-mentioned equity stratifiers)
    1. Pro-equity (perceptions of CHW programmes being fair, equitable, or reaching marginalised groups)
    2. Anti-equity (perceptions of CHW programmes being unfair, inequitable, or excluding or failing to reach some marginalised groups)
    3. Other (other comments, views or perspectives on CHWs and health equity that don’t fit into the above categories; this could include for example CHWs’ suggestions of measures that could improve equitability in the future)

**II. Wider contributions of CHWs to Health Equity**

1. Addressing Social Determinants (any mention of CHWs’ role in addressing social inequalities that contribute to health inequities)
2. Individual CHWs Overcoming Equitability Barriers (individual CHW-level strategies for overcoming equitability barriers, i.e. strategies **not** built into intervention design or formal roles)

**III. Inequities Affecting CHWs (**any mention of how the same axes of social inequality mentioned above or the health inequities they contribute to affect CHWs’ own wellbeing or professional performance)
