# Supplementary material for: Community health workers and health equity in low- and middle-income countries: systematic review and recommendations for policy and practice
Source: Int J Equity Health. 2022 Apr 11;21:49. doi: 10.1186/s12939-021-01615-y (PMC8996551; doi:10.1186/s12939-021-01615-y)
Supplement: Supplementary file 3 — Additional file 3. Characteristics of Studies Included in Qualitative Synthesis: table including study characteristics and methodological quality ratings for included studies containing qualitative evidence. [file 12939_2021_1615_MOESM3_ESM.docx]

**Additional File 3. Characteristics of Studies Included in Qualitative Synthesis^a^**

**Table A3-1. Characteristics of Studies Included in Qualitative Synthesis**

| Authors | Year | | Methods | | Health issue addressed | | Study population | | Total sample size | | | Quality appraisal | |  |
| --- | --- | --- | --- | --- | --- | --- | --- | --- | --- | --- | --- | --- | --- | --- |
| Adongo et al. | 2014 | | Other ('qualitative appraisal' following consultations with key stakeholders) | | Primary care | | Key stakeholders | | Not specified | | | Thin | |  |
| Ahmed et al. | 2017 | | Individual & group interviews | | MNCH | | Community Midwives (CMWs), lady health supervisors and managers | | 134 | | | Satisfactory | |  |
| Akeju et al. | 2016 | | Group interviews | | MNCH | | Pregnant women, recently pregnant mothers, male decision-makers, opinion leaders, traditional birth attendants (TBAs), community health extension workers (CHEWs), nurses and midwives | | 403 | | | Satisfactory | |  |
| Asher et al. | 2018 | | Individual & group interviews | | Mental health | | people with schizophrenia and their families | | 21 | | | Satisfactory | |  |
| Avery et al. | 2017 | | Group interviews | | MNCH | | CHVs | | 40 | | | Thin | |  |
| Ayon et al. | 2019 | | Individual & group interviews | | SRH, mental health, HIV prevention | | women injection drug users and key stakeholders | | 45 at formative phase (all women who inject drugs) 44 at evaluation phase (14 women who inject drugs plus 30 stakeholders) | | | Satisfactory | |  |
| Ayon et al. | 2018 | | Individual & group interviews | | SRH & mental health | | women injection drug users | | 50 | | | Satisfactory | |  |
| Baum et al. | 2019 | | Individual interviews | | NCD (hearing) | | caregivers of children with hearing loss | | 26 | | | Satisfactory | |  |
| Beam et al. | 2019 | | Other (barriers to report survey) | | other (pork tapeworm, a leading cause of preventable epilepsy) | | households in intervention and control villages | | 166 | | | Thin | |  |
| Belaid & Ridde | 2015 | | interviews & observations | | MNCH | | women, traditional birth attendants, clinic management committees, and health workers and district health managers | | 177 | | | Thin | |  |
| Bergen et al. | 2018 | | individual & group interviews | | MNCH | | community members, religious leaders, health extension workers (HEW) and community volunteers (members of women and male development armies; WDA & MDA) | | 24 individual interviews; 12 FGDs with 6-12 participants each | | | Thin | |  |
| Buchner et al. | 2014 | | individual & group interviews | | Childhood illnesses | | Caregivers of children under 5, health workers, CHWs, and local leaders | | 106 | | | Satisfactory | |  |
| Canavati et al. | 2016 | | Individual & group interviews | | Malaria | | VMWs and MMWs | | 476 | | | Satisfactory | |  |
| Chourasia et al | 2017 | | Individual interviews | | Malaria | | Age-sex controlled two groups of Mitanins | | 10 | | | Satisfactory | |  |
| Corbin et al. | 2016 | | individual interviews | | HIV/AIDS | | women grassroots volunteers and staff at one NGO | | 18 | | | Key | |  |
| Datiko et al. | 2015 | | individual & group interviews | | TB | | health care providers (including health extension workers [HEWs] and community health promoters [CHPs]) | | 40 | | | Key | |  |
| de Lange & Mitchell | 2016 | | Participatory action research | | Social determinants of health (gender-based violence) | | women CHWs | | 5 | | | Satisfactory | |  |
| Dusabe-Richards et al. | 2016 | | Individual & group interviews | | TB & MCNH | | Health extension workers (HEWs), Health Centre Heads, District Health Officers, Zonal Health Department and Regional Health Bureau representatives | | 13 | | | Thin | |  |
| Edward et al. | 2015 | | Individual & group interviews | | Primary care | | Various stakeholders in CHW programme, Afghanistan | | 272 | | | Satisfactory | |  |
| Elazen et al. | 2016 | | individual intereviews | | RMNCH | | male health activists (MHAs) and various female CHWs (ASHAs, auxiliary nurse midwives [ANMs], anganwadi workers [AWWs]) | | 34 | | | Satisfactory | |  |
| Farmer et al. | 2017 | | individual & group interviews | | Family planning | | female community members, CHWs, nurses | | 159 | | | Satisfactory | |  |
| Feldhaus et al. | 2015 | | Individual interviews | | MNCH | | CHWs | | 60 | | | Satisfactory | |  |
| Flink et al. | 2016 | | individual & group interviews | | None specified | | COSA members (CHWs), health workers and managers, poor, vulnerable, and 'indigent' community members | | 297 | | | Key | |  |
| Fotso et al. | 2015 | | individual interviews | | RMNCH | | recently delivered women, ASHAs, ANMs, AWWs | | 35 | | | Key | |  |
| Geldsetzer et al. | 2017a | | Group interviews | | HIV (and other high-stigma, confidential health information) | | Household sampling, community members | | 169 | | | Thin | |  |
| George et al. | 2018 | | Individual interviews | | MNCH | | Stakeholders | | 56 | | | Thin | |  |
| Getnet *et al.* | 2017 | | Individual interviews | | TB | | HEWs | | 5 | | | Thin | |  |
| Gittings | 2016 | | Individual interviews | | HIV | | community care workers and clients (men with HIV) in South Africa | | 11 | | | Satisfactory | |  |
| Give et al. | 2015 | | Individual & group interviews | | None specified | | women caring for children under 5, community leaders, Agents Polivalentes Elementares (APEs) and their supervisors | | 96 | | | Key | |  |
| Gupta | 2017 | | Individual and group interviews | | MNCH | | community members, clinicians, and managers | | 75 | | | Key | |  |
| Hailerman et al. | 2017 | | Individual & group interviews | | Mental health (severe mental disorders) | | people with severe mental disorders, caregivers, health care professionals, and CHWs | | 70 | | | Key | |  |
| Jackson et al. | 2016 | | Individual interviews | | MNCH | | HEWs (who were interviewed and also interviewed women who had recently given birth at home or in a health facility and reported back) | | 16 HEWs and 45 women | | | Satisfactory | |  |
| Jackson et al. | 2016 | | Individual interviews | | MNCH | | HEWs (who were interviewed and also interviewed women who had recently given birth at home or in a health facility and reported back) | | 44 HEWs and 123 women | | | Satisfactory | |  |
| Jackson et al. | 2019 | | Individual & group interviews | | MNCH | | health extension workers | | 45 individual interviews + 17 group interview participants | | | Key | |  |
| Jacobs et al. | 2018 | | Individual & group interviews for process evaluation | | MNCH | | Those engaged with the intervention, including health care providers, SMAGs, and women with a child <1yo and living in the study community during the most recent pregnancy | | 78 | | | Satisfactory | |  |
| Karanja et al. | 2018 | | Individual & group interviews | | MNCH | | women who had recently delivered, health providers, chiefs, CHVs, key decision influencers, and TBAs | | 54 | | | Satisfactory | |  |
| Kea et al. | 2018 | | Individual & group interviews with community members, HEWs, health professionals | | MNCH | | HEWs, community members, TBAs, health professionals/coordinators | | 44 in-depth interviews + 14 FGDs | | | Satisfactory | |  |
| Khuzwayo et al. | 2018 | | Individual & group interviews with community members and CHWs | | Primary care | | Community members, CHWs, and community representatives | | 49 | | | Satisfactory | |  |
| Kok et al. | 2016 | | Individual & group interviews | | Primary care | | Community members, HSAs, members of the health sector (health centre, NGO representatives, district managers) | | 181 | | | Thin | |  |
| Kwon et al. | 2014 | | Individual & group interviews | | Childhood illnesses (iron deficiency anemia) | | Mothers, VHWs, community stakeholders | | 46 | | | Satisfactory | |  |
| Labonté et al | 2014 | | Qualitative synthesis of findings from 20 mixed methods research projects, incorporating analyses of qualitative data, secondary data, and key policy and program documents, case study analyses, and new surveys. | | Primary care | | Varied | | Varied | | | N/A | |  |
| Liverani et al. | 2017 | | Individual interviews | | Malaria | | VMWs, village authorities, forest goers and caregivers | | 71 | | | Key | |  |
| Loeliger et al. | 2016 | | Group interviews | | HIV/AIDS | | CHWs serving people with HIV in rural South Africa | | 21 | | | Key | |  |
| Lusli *et al.* | 2016 | | Individual & group interviews | | Leprosy (stigma) | | Leprosy patients and  other family members. | | 96 individuals interviewed (77 at baseline, 24 final; 5 of these were paired) 64 individuals took part in group interviews | | | Thin | |  |
| Mambulu-Chikankheni et al. | 2018 | | Individual interviews | | Childhood illnesses (severe acute malnutrition) | | CHWs and CHW leaders | | 15 (11 CHWs, 4 CHW leaders) | | | Satisfactory | |  |
| McCollum et al. | 2016a | | Individual & group interviews | | Primary care | | stakeholders in community health policy and programmes in Kenya (including managers, policy makers, CHWs, and beneficiaries) | | 124 | | | Key | |  |
| Miller et al. | 2014 | | Individual interviews | | Primary care | | VHWs and their supervisors | | 14 | | | Satisfactory | |  |
| Mumtaz et al. | 2014 | | Individual & group interviews, observations (institutional ethnography) | | MNCH | | community midwives, policy makers, community members, and healthcare providers in Pakistan | | 239 | | | Satisfactory | |  |
| Musoke et al. | 2018 | | Other: Photovoice and group discussions | | Primary care | | CHWs in Uganda | | 10 | | | Key | |  |
| Naidoo et al. | 2019 | | Individual & group interviews | | Childhood illnesses | | key informants: CHWs and health facility workers | | 32 | | | Satisfactory | |  |
| Najafizada et al. | 2019 | | Individual & group interviews | | None specified (general) | | CHWs and beneficiaries, health managers, policymakers | | 80 | | | Key | |  |
| Nandi & Schneider | 2014 | | Individual & group interviews to create a comparative case study between two sub-districts (Durgkondal and Manendragarh) | | Other (this is specifically looking at CHW action on nutrition or violence against women within their role in looking after MNCH and general health) | | stakeholders in the Mitanin (CHW) programme in India | | 17 individual interviews + 10 group interviews | | | Satisfactory | |  |
| Ngaya-an & Fowler | 2014 | | Individual & group interviews | | MNCH | | Community women who were volunteer health workers during the project, plus midwives and nutrition scholars | | 21 | | | Satisfactory | |  |
| Niyongabo *et al.* | 2018 | | Individual interviews | | MNCH | | community health workers, health committee members, health providers, local authorities, religious leaders  and managers of non-governmental organizations | | 138 | | | Thin | |  |
| Nwameme et al. | 2018 | | Individual interviews | | None specified (general health care delivery) | | Community health officers, their supervisors, District health people and nurses | | 19 | | | Satisfactory | |  |
| Ochieng et al. | 2014 | | Individual & group interviews | | None specified (general health care delivery) | | various stakeholders in CHW programme in Kenya, including health policy makers, managers and service providers, CHOs and service consumers | | 98 | | | Thin | |  |
| Owek et al. | 2017 | | Individual & group interviews | | Malaria | | mothers of children under 5 and key stakeholders in Kenya | | 100 CHWs, 100 community members, 10 CHEWs, 10 clinicians, and the PHO and district or county director of health for each of the five districts (so 230?) | | | Thin | |  |
| Panday et al. | 2017 | | Individual & group interviews | | MNCH | | female community health volunteers (CHWs), beneficiaries, and health workers in Nepal | | 56 | | | Key | |  |
| Panday et al. | 2019 | | Individual & group interviews | | MNCH | | female community health volunteers (CHWs), ethnic minority women, and health workers in Nepal | | 72 | | | Satisfactory | |  |
| Pelcastre-Villafuerte et al. | 2014 | | Individual interviews (and archival research) | | Basic health care among women with special emphasis on reproductive health care referrals | | stakeholders in in Casa intervention for indigenous women in Mexico (coordinators and advisors, operations personnel, women receiving services, and other participants) | | *62* | | | Key | |  |
| Puett et al. | 2015 | | Group interviews | | Preventive care and community case management of common childhood illnesses | | CHWs in Bangladesh | | 83 | | | Satisfactory | |  |
| Rafiq et al. | 2019 | | Ethnographic (individual and group interviews, participant observation) | | RMNHC plus preventive and curative services, disease surveillance | | CHWs, supervisors, and clients in rural Tanzania | | 88 individual interviews, 24 group interviews (averaging 12 respondents), observation of 6 WAJA over 12 weeks | | | Key | |  |
| Rahmawati & Bajorek | 2015 | | Individual interviews and one-off observation of a weekly meeting | | NCD (hypertension) | | members of the IHSP-Elderly program (patients aged ≥60, involved in IHSP for ≥1 year, diagnosed with hypertension), CHWs serving the IHSP-Elderly program, and a district health staff member | | 15 | | | Thin | |  |
| Saprii et al. | 2015 | | Individual & group interviews | | MNCH | | ASHAs (CHWs), community members, key stakeholders | | 36 | | | Key | |  |
| Sayinzoga et al. | 2019 | | Individual & group interviews | | RMNHC | | CHWs, nurses, social affairs officers | | 16 group interviews (6-7 participants per) plus 3 key informant interviews | | | Thin | |  |
| Shah et al. | 2018 | | Individual & group interviews | | MNCH | | mothers, husbands, in-laws, FCHVs (CHWs), TBAs, healthcare providers, district health managers | | 12 interviews and 10 focus group discussions | | | Satisfactory | |  |
| Shaikh et al. | 2017 | | Group interviews | | MNCH | | delivered mothers & husbands, including members and non-members of community-based savings groups | | 16 focus groups (each contained 6-10 participants) | | | Thin | |  |
| Sharkey et al. | 2014 | | multiple programme evaluations (qualitative process evaluation, baseline and endline surveys, qualitative study of stakeholders' perceptions) | | Childhood illnesses | | stakeholders in iCCM programmes | |  | | | Thin | |  |
| Sharma et al. | | 2014 | | Individual interviews | | None specified (general access to health care) | | ASHAs, their coworkers, and local representatives | | 63 | | Thin | |  |
| Shaw et al. | | 2016 | | Individual & group interviews, rapid ethnographic assessment | | Childhood illnesses | | mothers of children under 5, fathers, health extension workers & community health volunteers (CHWs) | | 210 | | Key | |  |
| Shaw et al. | | 2017 | | Individual & group interviews, rapid ethnographic assessment | | Childhood illnesses | | mothers of children under 5, fathers, health extension workers & community health volunteers (CHWs) | | 210 | | Satisfactory | |  |
| Sibley et al. | | 2017 | | Individual interviews & individual/family narratives | | MNCH | | mothers and witnesses to newborn illness events | | 51 in narratives | | Satisfactory | |  |
| Steege et al. | | 2018 | | individual & group interviews | | None specified | | CHWs, community members and other key stakeholders in Bangladesh, Ethiopia, Indonesia, Kenya, Malawi, Mozambique | | unclear (FGD size not specified) | | Satisfactory | |  |
| Stollak et al. | | 2016 | | Individual & group interviews | | MNCH | | women who had delivered within first year of intervention, community leaders, health workers, and TBAs | | 22 women who delivered; 42 community leaders, TBAs, staff members | | Satisfactory | |  |
| Taleb et al. | 2015 | | Individual & group interviews | | MNCH | | Pregnant women, mothers who gave birth recently, husbands, family members, HWs, TBAs, CHWs | | | | 67 | | Satisfactory | |
| Tancred et al. | 2018 | | Individual & group interviews + birth narratives | | MNCH | | VHV and groups, leaders, mothers and fathers, NGO And Government representatives | | | | 134 | | Thin | |
| Tefera et al. | 2014 | | Individual & group interviews | | Childhood illnesses | | community members, mothers of recently ill children, key informants, and HEWs | | | | 60 mothers, 6 HEWs, 6 HWs | | Thin | |
| Tschirhart et al. | 2017 | | Individual & group interviews | | TB | | TB patients and key stakeholders | | | | 15 FGDs, 13 individual interviews | | Satisfactory | |
| Tulloch et al. | 2015 | | Individual interviews | | TB | | Intervention beneficiaries (screened and tests via the intervention and were either on TB treatment or had a negative smear result) | | | | 36 | | Thin | |
| Wester et al. | 2018 | | Individual interviews | | RMNHC | | health care providers | | | | 12 | | Thin | |
| Wharton-Smith et al. | 2019 | | Individual & group interviews | | Neglected tropical diseases | | community members, volunteer health development army leaders (CHWs), health extension workers (CHWS), health workers | | | | 59 | | Satisfactory | |
| Woods-Jaeger et al. | 2017 | | Individual interviews | | Mental health | | lay counsellors delivering intervention in Kenya and Tanzania | | | | 12 | | Satisfactory | |

^a^ For mixed-methods studies, information on the qualitative portion of the study only is included in this table.
