# Supplementary material for: Community health workers and health equity in low- and middle-income countries: systematic review and recommendations for policy and practice
Source: Int J Equity Health. 2022 Apr 11;21:49. doi: 10.1186/s12939-021-01615-y (PMC8996551; doi:10.1186/s12939-021-01615-y)
Supplement: Supplementary file 4 — Additional file 4. Characteristics and Findings by Outcome and Equity Stratifier of Studies Included in Quantitative Synthesis: a series of tables including study characteristics and methodological quality ratings for included studies containing quantitative evidence, along with details of each study’s findings by outcome and equity stratifier. [file 12939_2021_1615_MOESM4_ESM.docx]

# Additional File 4. Characteristics and Findings by Outcome and Equity Stratifier of Studies Included in Quantitative Synthesis

**Table A4-1. Studies on Maternal Care-Focused CHW Interventions**

| Authors & Year | Characteristics | CHW Intervention | Summary results | Outcome by Equity stratifier |
| --- | --- | --- | --- | --- |
| Afework et al. 2014^(28)^* | Design: Cross-sectional  Duration: 9 years.  Population: Women who gave birth in previous 2 years  Quality: Good  Country: Ethiopia | HEW; home visits, CHW conducted ANC, PNC, and provided maternal and neonatal care services. | Visits by HEWs was associated with greater ANC attendance and facility delivery | **ANC:** Rural residence^↓^_,_ high women’s education^↑^, marital status^NS^, low wealth quintile^↑^  **Facility delivery:** Rural residence^↓^_,_ high women’s education^↑^, marital status^NS^, high wealth quintile^↑.^  **PNC:** Place of residence^NS^, high women’s education^↑^, unmarried^↓^, low wealth quintile^↑^ |
| Agarwal et al. 2019^(29)^* | Design: Quasi-experimental  Duration: Survey data of 2004–2005 and 2011–2012  Population: Women of childbearing age  Quality: Good  Country: India | ASHAs; home visits, counselling, CHW conducted ANC and PNC visits, and facilitate use of skilled birth attendance, provided incentives to women to  give birth in health facilities. | There were substantial inter-state variations in the receipt of ASHA services but it reached successfully women from poor and backward castes. | **Receipt of ASHA services for the most recent birth:** Place of residence ^NS^, high women’s education^↑^, low wealth quintile^↑.^, marginalised or scheduled caste^↑^, religion ^NS^. |
| Asiki et al. 2018^(47)^ | Design: Non RCT, pre-post, intervention vs control  Duration: 1.5 years  Population: Pregnant/post-partum women, rural  Quality: Good  Country: Uganda | CHW, VHW (village health workers; home visits, mobile message to register pregnancy, arrange visits and educate | The intervention led to lower odds of homebirths | **Homebirths:** Distance^↑^, mother’s education^↓^, Muslim^↑^ |
| Barry et al. 2014^(132)^* | Design: Quasi-experimental  Duration: 2 years  Population: Women who had given birth in past year, rural  Quality: Good  Country: Ethiopia | Female volunteers, HEW; family meetings to build skills to identify maternal threats and care-seeking behaviours, provide ANC, PNC, SBA | HEWs increased in women receiving complete MNH care | **Use of a SBA at Birth:** Mother’s education^↑^  **Community MNH Package coverage:** Mother’s education^NS^ |
| Brooks et al. 2019^(125)^* | Design: Cross-sectional  Duration: <3 years  Population: married women of 13 to 19 years and their husbands, rural  Quality: Fair  Country: Niger | Relais communautaires; home visits, health promotion, link with health facilities, distribution of family planning commodities | The use of  modern family planning methods almost doubled with relais visits | **Modern family planning:** Women’s unemployment^↑^, tribes^NS^, mother’s and father’s education^NS^  **CHW visit coverage:** Minority tribes^↑^ |
| Burke et al. 2019^(120)^* | Design: RCT  Duration: 1 year  Population: Rural women  Quality: Good (low risk)  Country: Malawi | CHWs; injectable contraceptive intervention | CHWs were equally effective in administering injectable contraceptive intervention as clinic based providers | **Injectable contraceptive continuation rates:** Women’s education^NS^, marital status^NS^, women’s employment^NS^, religion^NS^ |
| Choulagai et al. 2017^(121)^ | Design: Cluster RCT  Duration: 1 year  Population: women having given birth in the past year  Quality: Good (low risk)  Country: Nepal | Female community health volunteers (FCHVs); increased family support, financial and transportation assistance to health facility, and SBA security. | Intervention resulted in better use of skilled birth care services (OR: 1.57; 95% CI: 1.19–2.08). | **SBA utilisation:** High mother’s education^↑^, women’s occupation^NS^, Ethnicity^NS^  **More than four ANCs:** Ethnicity^NS^  **One ANC:** Low caste^↑^ |
| Darega et al. 2016^(126)^* | Design: Cross-sectional  Duration: Not reported  Population: Mothers, rural  Quality: Good  Country: Ethiopia | HEWs; home visits, HEW conducted ANC, PNC, skilled birth attendance | HEWs and other sources provided MNCH services where the former was found to be utilised for PNC support by majority (91.5%) | **Facility delivery:** Greater distance^↓^, mother’s education and occupation^NS^, husband’s education^↑^ and occupation as merchants^↑^  **PNC:** Greater distance^↓^, husband’s education^↑^, husband’s occupation as merchants^↑^, unemployment of husbands^↓^ |
| Edmond et al. 2019^(84)^ | Design: Non RCT, quasi-experimental  Duration: 1 year  Population: Rural pregnant women  Quality: Good  Country: Afghanistan | CHW; home visits, education and incentives for institutional delivery | Intervention of CHW with incentives improved ANC, PNC utilisation (but not facility delivery) when compared to controls (CHW services without incentive). | **ANC, PNC:** High SES quintile^↑^  **Facility delivery:** SES quintile^NS^  CHW visit coverage: High SES quintile^↑^ |
| George et al. 2018^(122)^ | Design: Non RCT, Cross-sectional  Duration: 3 years  Population: Women  Quality: Good  Country: India | ASHAs and AWWs; home visits, education, and distribution of learning materials, community actions | Reported increases in receipt of information and utilisation of antenatal and delivery care in the marginalised districts | **Facility delivery:** High mother’s education^↑^, women’s employment^NS^, caste^NS^ |
| Gonzalez-Casanova et al. 2017^(97)^* | Design: RCT  Duration: Not mentioned  Population: Women of reproductive age  Quality: Fair (Moderate risk)  Country: Vietnam | VHWs; home visits, education, micronutrient supplementations, follow-up and referral. | The intervention and VHW visits resulted in high adherence for supplementation. | **Supplement consumption:** High SES quintile^↑^  **Adherence:** Mother’s education^NS^, high SES quintile^↑^, husband’s unemployment^↓^ and husbands being farmers^↓^, minority ethnicity^↓^. |
| Gudu et al. 2017^(104)^* | Design: Cross-sectional  Duration: 5 years  Population: Women who gave birth  Quality: Good  Country: Ghana | CHO (community health officers); home visits, CHW conducted ANC, PNC, skilled delivery services and referral | CHO implementation resulted in a high  proportion of women who received ANC and utilised SBA (91.5%) and satisfaction with ANC services was high. | **Skilled delivery:** High mother’s education^↑^, marital status^NS^, SES quintile^NS^ |
| Huq et al. 2015^(49)^* | Design: Quasi-experimental  Duration: 2 years  Population: Pregnant women  Quality: Good  Country: Bangladesh | CSBAs (community skilled birth attendants); counselling, safe delivery kit distribution, promotion of skilled delivery, detection and referral, skilled delivery at home, pregnancy, post-partum and neonatal care | Intervention had an  effect on the individual performing area on use of skilled provider care during ANC, delivery and PNC; even in low-performing area that are mostly in hard to reach location. | **ANC:** Hard to reach area^↑^, high mother’s education^↑^, husband’s education^↑^, high SES quintile^↑^  **SBA utilisation:** Hard to reach area^↑^, high mother’s education^↑^, husband’s education^NS^, high SES quintile^↑^  **PNC:** Mother’s education^↑^, husband’s education^NS^, high SES quintile^NS^ |
| Jacobs et al. 2018^(197)^ | Design: Quasi-experimental  Duration: 2 years  Population: Mothers of <5 months babies  Quality: Fair  Country: Zambia | CHW, trained TBAs, Safe Motherhood Action Groups (SMAGs); intervention of health education, promotion and pregnancy referral | The intervention led to two-threefold increase in the odds of mothers receiving ANC, and PNC from skilled providers | **ANC:** Mother’s literacy^NS^, greater distance^↓^  **SBA use:** Mother’s literacy^↑^, greater distance^↓^  **PNC:** Mother’s literacy^↑^, distance^NS^ |
| Jolly et al. 2016^(91)^* | Design: Cross-sectional  Duration: Not reported  Population: pregnant women  Quality: Good  Country: Bangladesh | MANOSHI CHW; Home visits, health education, pregnancy identification, follow-up and referral, CHW provided and promoted ANC and PNC support and supplies safe delivery kit | The intervention improved ANC and PNC coverage in the intervention slums but 50% deliveries were still not conducted by SBA. | **ANC:** Mother’s literacy^↑^, husband’s literacy^NS^, high SES quintile^↑^  **Use of SBA:** mother’s literacy^↑^, husband’s literacy^NS^, high SES quintile^↑^  **PNC:** mother’s literacy^NS^, husband’s literacy^NS^, high SES quintile^↑^  **Treatment seeking for delivery complications:** mother’s literacy^↑^, husband’s literacy^NS^, high SES quintile^↑^  **Modern family planning:** mother’s literacy^NS^, husband’s literacy^NS^, low SES quintile^↑^ |
| Juma et al. 2015^(130)^* | Design: Cross-sectional  Duration: 3 years  Population: Women aged 15-49 y  Quality: Poor  Country: Kenya | CHW; RMNCH reproductive education, pills and condoms and referral services | Only a third of the respondents exhibited high acceptance for CHW-delivered family planning services | **Acceptance of family planning:** Lower women’s education^↑^, Ethnicity^NS^, Religion^NS^, |
| Karanja et al. 2018^(44)^ | Design: Cross-sectional  Duration: Not reported (intervention was there since 2007)  Population: Pregnant women, semi-mobile populations  Quality: Good  Country: Kenya | CHV, trained TBAs; links community with the formal health care system to improve MNCH utilisation | 39% of the women had delivery at the health facility. | **Health facility delivery:** Greater distance^↓^, women’s education^↑^ & occupation^NS^, high SES quintile^↑^, married^↓^, religion^NS^ |
| Karim et al. 2015^(51)^ | Design: Cross-sectional surveys (2008, 2010)  Duration: Not reported, HEW working since 2003  Population: pregnant women, newborns, and children  Quality: Good  Country: Ethiopia | CHP (community health promoter), & HEWs; free health care, education, health promotion & community mobilisation, behaviour change and more equity-specific HEP implementation since 2008 | With the HEW services, access to health care increased and improvements in 17 out of 19 MNCH indicators were found. Treatment receipt rates for childhood diarrhoea and acute respiratory infection (ARI) did not improve. | **Birth preparedness:** Women’s education^↑^, Distance^NS^  **Delivery at health facility:** Women’s education^↑^, Wealth index^↑^  **Delivery by SBA:** Women’s education^↑^  **HEW activity coverage:** Women’s education^NS^  **Newborn cord care:** Women’s education^NS^, greater distance^↓^  **Households possessing family health card:** Distance^NS^, wealth index^↑^  **Thermal care for neonates:** Wealth index^↑^  **HEW household visits:** Distance^NS^, Wealth index^NS^  **Model family households:** Distance^NS^, Wealth index^NS^  **Family planning:** Wealth index^NS^ |
| Kawakatsu et al. 2014^(45)^ | Design: Cross-sectional  Duration: >3 years  Population: Mothers with children aged 12–23 months  Quality: Good  Country: Kenya | CHW; door-to-door collection of health-related data and education on health-related preventive methods | 48% of births took place in the health facility and  involvement of TBAs was one of the most  important strategies to promote it. Performance of CHWs also had effect. | **Facility delivery:** Greater distance^↓^,  mother’s education^↑^ & health knowledge^↑^, high SES quintile^↑^, occupation ^NS^, social capital ^NS^. |
| Kelbessa *et al.*  2014^(117)^ | Design: Cross-sectional  Duration: Not reported; HEW working since 2003  Population: Household heads  Quality: Poor  Country: Ethiopia | HEW; Home visits, outreach activities, immunisations and providing contraceptives, case detection and treat malaria, dysentery, intestinal parasites and other ailments and referral. | The proportion of community utilisation of health extension service was 39%. Occupation, knowledge about HEW, community participation were predictors of utilisation. | **HEW service utilisation** (includes both maternal and other services): Occupation as government employment^↑^ |
| McDougal et al. 2017^(98)^ | Design: Quasi-experimental  Duration: Not reported  Population: Mothers of 0-5 month old child  Quality: Good  Country: India | Ananya FLWs, ASHAs and AWWs; home visits, improve health behaviours and promote service utilisation. | Intervention resulted in  significantly greater increase in RMNH co-coverage over time. | **RMNCH CoC:** Gender ^NS^, high SES quintile^↑^, scheduled caste^↓^, Muslims^↓^, mother’s education^↑^, husband’s education^↑^ |
| Muhumuza Kananura et al. 2017^(105)^ | Design: Non RCT pre-post design  Duration: 2 years  Population: Women with recent delivery  Quality: Good  Country: Uganda | CHW and village health teams; Home visits, MNCH counselling, referral, community mobilisation | The intervention improved participants’ knowledge about maternal and newborn danger signs and birth preparedness practices | **Birth preparedness:** Mother’s education ^NS^ & occupation ^NS^, Muslims and minority religion^↓^, SES quintile ^NS^  **Knowledge about pregnancy and danger signs:** Mother’s education^↑^ & occupation ^NS^, religion^NS^, SES quintile ^NS^. |
| Namukwaya et al. 2015^(39)^ | Design: Cross-sectional  Duration: Not reported  Population: Pregnant HIV women  Quality: Good  Country: Uganda | Peer counsellors; home visits for education, promotion and couple counselling and phone calls to follow up, and link to health facility | The intervention caused improvement in PNC attendance from 37.7 % to 78.5% at 6 weeks and early infant diagnosis | **PNC attendance**: Rural residence^↓^, Mother’s education ^NS^ and employment ^NS^, father’s employment ^↓^, marital status^NS^. |
| Negero et al. 2018^(38)^ | Design: Cross-sectional  Duration: >6 years  Population: Women with recent delivery  Quality: Good  Country: Ethiopia | Women’s Health Development Army (WHDA) and HEW; health and nutritional education and promotion for care utilisation and link to health facility, disease control, immunisation, family planning. | 45% of women utilised SBAs. A significant heterogeneity in SBA utilisation was observed between WHDA clusters. | **SBA utilisation**: Rural residence^↓^, greater distance^↓^, mother’s education^NS^, husband’s education^NS^ |
| Negussie et al. 2017^(131)^* | Design: Cross-sectional study  Duration: Not reported  Population: Women with recent delivery  Quality: Good  Country: Ethiopia | HEW; promotive, preventive and selected high impact curative health services, ANC, PNC, skilled delivery and family planning and basic treatment for child | Service coverage of ANC, delivery and PNC were low as the input and number of home visits  from the HEWs, were unsatisfactory and inadequate to support the mothers. | **Maternal and child health service (3 or more) utilisation:** Low mother’s Education^↑^, ethnicity ^NS^, religion |
| Peltzer et al. 2018^(110)^ | Design: Cluster RCT  Duration: 3 years  Population: perinatal HIV +ve women  Quality: Poor (high risk)  Country: S. Africa | Trained lay health workers; ANC, PNC group sessions on HIV knowledge, HIV tests, stigma, disclosure, infant feeding, safer conception and family planning. | CHWs were effective in decreasing HIV related stigma through behavioural intervention in postnatal women | **HIV related stigma:** Mother’s Education^NS^, SES quintile^NS^, marital status^NS^, being employed^↑^ |
| Sakeah et al. 2014^(37)^* | Design: Cross-sectional  Duration: NA (Ongoing from 2000)  Population: women who had given birth in last three years  Quality: Good  Country: Ghana | Community health officer-(CHOs) and midwives; Health education and management of minor ailments and CHO provided skilled birth attendance | CHO-midwives’ intervention resulted in over half of the skilled deliveries being conducted by them. | **Skilled delivery attendance:** Distance^NS^, mother’s Education^NS^, husband’s education^↑^, women’s occupation^NS^, religion ^NS^, minority ethnicity^↓^ |
| Sam-Agudu et al. 2017^(135)^ | Design: Quasi-experimental  Duration: Not reported  Population: HIV affected mother and their infants  Quality: Good  Country: Nigeria | Mentor mother (MM) and peer support; Education and psychosocial support to access services, maintain and adhere to treatments. | The intervention reduced total viral load and improved adherence to treatment | **Viral suppression and retention to treatment:** Distance^NS^, Mother’s Education^NS^, Religion^NS^, Marital status^NS^ |
| Seth et al. 2017^(95)^ | Design: Cross-sectional  Duration: Around 1 year  Population: Women who had a live birth in last 12 month  Quality: Good  Country: India | ASHA; Health education, delivery of basic health care, and promoting uptake | When interactions were checked between CHW visits & socio-demographic factors), CHW visits increased the odds of ≥4 ANC visits only among non-Muslim women, increased  both ≥ANC visits and facility delivery only among lower wealth women, facility delivery to a greater degree among illiterate women. | **CHW visit coverage:** Mother’s literacy ^NS^, caste^NS^, religion^NS^, SES quintile^NS^  **ANC:** Mother’s literacy^↑^, Muslim^↓^, low/scheduled caste^↓^, high wealth quintile^↑^  **Facility delivery:** Illiterate Mothers^↑^, Muslim^↓^, low/scheduled caste^↓^, high wealth quintile^↑^.  **Family planning:** Mother’s literacy^↑^, Muslim^↓^, Low/scheduled caste^↓^, high wealth/SES quintile^↑^. |
| Sibley et al. 2017^(87)^* | Design: Cross-sectional  Duration: 2 years during 2010 & 2012 surveys  Population: Perinatal women and newborns  Quality: Good  Country: Ethiopia | CHVs, HEWs; home visits, ANC, PNC and delivery care through HEWs, community meetings and mobilisation | Women who attended family meetings were more likely to seek biomedical care for pregnancy complications and to use health facility or HEWs for delivery. | **Care seeking behaviour in pregnancy complications:** Mother’s education ^NS^, low SES^↑^ |
| Spangler et al. 2014^(50)^ | Design: Cross-sectional (2 surveys)  Duration: 2 years during 2010 & 2012 surveys  Population: Women who gave birth in the past year  Quality: Good  Country: Ethiopia | HEWs, volunteer community health development agents, local TBAs; Health education, community meetings for mobilisation | Between baseline & endline surveys, delivery by SBA and PNC increased and use of untrained providers decreased. | **MaNHEP programme participation**: Distance ^NS^, mother’s education ^NS^, high SES^↑^  **SBA utilisation:** Mother’s education^NS^, low SES^↑^  **PNC:** Low SES^↑^ |
| Stollak et al. 2016^(46)^ | Design: Cross-sectional  Duration: 2 years (2011-13)  Population: Women who gave birth  Quality: Good  Country: Guatemala | Volunteer community peer educators; Peer counselling and home visit to promote healthy behaviours and appropriate use of health facilities, reporting vital events. | The intervention led to greater health facility delivery in CHW partner communities than non-partner communities (70% vs 30%) | **Health facility delivery:** Greater distance^↓^ , high SES^↑^, mother’s education ^NS^ |
| Tesfaye et al. 2014^(129)^* | Design: Cross-sectional  Duration: 2 years (2010-12)  Population: Women who gave birth in the past year  Quality: Good  Country: Ethiopia | HEWs, CHDAs, and TBAs; education and promotion through community and family meetings, registering for ANC and notify HEWs for conducting PNCs | Significant increase in women receiving PNC from a HEW was found which was associated with their participation in MNH family meetings and receipt of ANCs. | **PNC:** Mother’s education^NS^, wealth (mobile phone ownership)^NS^ |
| Vellakkal et al. 2017^(86)^ | Design: Quasi-experimental (survey data)  Duration: Pre-(1999-2006), Post-(2007-12)  Population: Households in deprived states  Quality: Good  Country: India | ASHA and NRHM CHWs; Cash transfers to women for institutional delivery and to ASHAs for ANC | Increase in ANC and institutional delivery was found steeper  in late post-NRHM periods (2011-12). Inequity declined in ANC, PNC and in receipt of financial incentives. | **Facility delivery:** Low SES^↑^, low education of either women or their husbands^↑^ |
| Wagner et al. 2018^(102)^ | Design: Cross-sectional  Duration: Not reported  Population: pregnant women  Quality: Fair  Country: India | CHW; Identify high-risk pregnant women, provide health education and referral | CHW support resulted in improvement in health facility delivery rates (90%) | **Facility delivery:** Women’s occupation ^NS^, SES quintile^NS^ |

**Table A4-2. Studies on Child-Focused CHW interventions**

| Authors & Year | Characteristics | CHW Intervention | Summary results | Outcome by Equity stratifier |
| --- | --- | --- | --- | --- |
| Ara et al 2018^(133)^ | Design: Cluster RCT  Duration: Two years  Population: Mother-infant pairs  Quality: High  Country: Bangladesh | Peer counsellors; home visits, peer educational counselling for mothers and family members | Peer counselling resulted in more mothers practising early and exclusive breastfeeding practices | **Early breastfeeding:** Mother’s education ^↑^  **Exclusive breastfeeding:** Mother’s education^NS^ |
| Ashenafi et al. 2014^(31)^* | Design: Quasi-experimental (survey data)  Duration: 1 year  Population: Mothers with <5 children, rural  Quality: Good  Country: Ethiopia | HEW; scaled up iCCM with treatment and increased intensity of services to improve care-seeking behaviour in childhood sicknesses | iCCM implementation in HEW services resulted in increased care seeking behaviour for sick newborns | **Care seeking behaviour:** SES quintile^NS^, greater distance^↓^, no road access^↓^ |
| Bâ et al. 2018^(85)^* | Design: Repeat cross-sectional  Duration: 3 years (2008-10)  Population: Children aged under 10 years  Country: Senegal  Quality: Good | Community health agents; home visits, promotion and home delivery of seasonal malaria chemoprevention (SMC) | Door-to-door can achieve high equitable coverage for SMC and high-quality delivery. Higher coverage of receipts of three treatments was noted among school-age children (96%) than pre-school children (90%). | **Chemoprevention:** Mother’s education^NS^, SES quintile^NS^  **Bednet use:** SES quintile^↑^ (Education was not checked) |
| Gill et al. 2014^(134)^* | Design: Cross-sectional  Duration: 2 years  Population: Rural live-born neonates  Quality: Fair  Country: Zambia | TBA or Auxillary CHWs; home visits, treatment and referral for neonatal infection | TBAs conducted a  home visits in 50% participants and referred 11% for suspected sepsis. | **Newborn survival:** Mother’s education^NS^, girl babies^↑^  **Exclusive breastfeeding:** Girl babies ^↑^ |
| Gope et al. 2019^(90)^* | Design: Non RCT (pre-post)  Duration: 3 years  Population: under 3 children and their mothers; rural  Quality: Fair  Country: India | Community-based facilitator; home visits, counselling, participatory Learning plus in another group crèches providing food supply & growth monitoring services. | Combination of Crèches, PLA meetings and home visits were more effective in reducing wasting, stunting & underweight in children | **Wasting:** Most marginalised families^↓^ (defined as those from Scheduled Tribes and poorest wealth quintiles). |
| Ijumba et al. 2015^(107)^* | Design: Cluster RCT  Duration: around 1 year  Population: pregnant and post-partum women with newborn  Quality: Good (low risk)  Country: S. Africa | CHWs; maternal and newborn care package (advanced), ANC and PNCs and mobile messages for new pregnancy notification | CHWs with advanced care package intervention doubled exclusive breastfeeding practices at 12 weeks post-partum (aOR: 2·31, 95% CI: 1·82, 2·93) but had no impact on early breastfeeding initiation. | **Exclusive breastfeeding:** Mother’s education^NS^, household wealth ^NS^ |
| Jefferds et al. 2015^(80)^* | Design: Cross-sectional surveys  Duration: 3 months and 15 months  Population: Mothers of children aged 6–23 months  Quality: Good  Country: Nepal | Female community health volunteers (FCHVs); routine distribution of micronutrient supplementation and behaviour change support | The use of CHWs (female health volunteers) was found to be associated with increased efficiency over health facilities for micronutrient powder (MNP) sachets distribution in Nepal | **Supplementation coverage:** Gender^NS^, mother’s education^↑^, lower SES quintile^↑^ |
| Johri *et al.*  2015^(99)^ | Design: Cluster RCT  Duration: 4 months  Population: Mothers of children aged 0–23 months  Quality: Good (low risk)  Country: India | Community volunteers; Home visits, education for improving knowledge, awareness and attitudes towards immunisation, community mobilisation | Intervention resulted in better vaccination knowledge in participating mothers | **Child vaccination knowledge:** High mother’s education^↑^, high SES^↑^ |
| Kawakatsu et al. 2017^(43)^ | Design: Cross-sectional  Duration: 10 years  Population: Children under five and their families  Quality: Good  Country: Kenya | CHW; health education on the prevention of diarrhoea, water safety, sanitation and hygiene. | CHWs can have a positive health effect through promoting appropriate health-seeking behaviour and treatment for childhood diarrhoea. | **Prevalence of diarrhoea:** Rural residence^↓^, male baby^↑^, gender of HH head ^NS^, mother’s education ^NS^, wealth index^NS^, water treatment available^↓^ & hand washing facility available^NS^  **Appropriate diarrhoea treatment:** Residence ^NS^, gender of baby & HH head ^NS^, mother’s education ^NS^, SES quintile^↑^ |
| Kawakatsu et al. 2015^(100)^ | Design: Cross-sectional  Duration: Not reported  Population: Mothers of under five children  Quality: Good  Country: Kenya | CHW and CHEW; Mass immunisation campaigns, home visits, health education and behaviour, logistics for vaccination. | With CHW intervention, the full vaccination coverage was 76.6% and participation in the  campaigns could improve complete vaccination coverage | **Vaccination coverage:** Gender ^NS^, SES quintile^↑^ |
| Kimani-Murage et al. 2017^(92)^ | Design: Cluster RCT  Duration: 2 years  Population: pregnant girls and women (12-49 years old) in urban poor setting  Quality: Good (low risk)  Country: Kenya | CHW; Home visits, education & nutritional counselling, detection and referral of HIVs. | CHW intervention was equally effective in both arms receiving CHW intervention with or without maternal infant and young child  nutrition (MIYCN) messages. | **Exclusive breastfeeding:** Female baby^↓^, mother’s education^NS^ & unemployment^↑^, ethnicity^NS^, religion^NS^, low SES quintile^↑^ |
| Kimani-Murage et al. 2016^(106)^ | Design: Non RCT (Pre and post intervention)  Duration: 3 years  Population: Mother-infant pair  Quality: Good (low risk)  Country: Kenya | CHW; Home visits, education & nutritional counselling on maternal nutrition, EBF and complementary feeding +some HIV counselling and referral | Both intervention and controls with or without advanced CHW components on counselling led to a 53% improvement in EBF rates at 6 months compared to the pre-intervention phase | **Exclusive breastfeeding:** Gender^NS^, mother’s education ^NS^ & formal employment^↓^, minority ethnicity^↓^, SES quintile ^NS^ |
| Lee et al. 2019^(108)^ | Design: Cross-sectional  Duration: Not reported  Population: Children aged between 6-18 months  Quality: Good  Country: Ethiopia & India | CHW; Management of vaccination process: recording status and informing parents about vaccination benefits and risks | The gap in vaccination between literate and illiterate mothers was smaller in communities with CHWs compared to communities without. | **Vaccination coverage:** Gender ^NS^, SES quintile ^NS^, mother’s literacy ^↑^, father’s literacy ^NS^ |
| Manu et al. 2016^(23)^ | Design: Prospective cohort  Duration: One year  Population: Sick newborns  Quality: Poor  Country: Ghana | Community-based surveillance volunteer (CBSV); Home visits, promote newborn care practices and service use, weigh and assess newborns, and refer to health facilities | CBSV intervention increased care seeking in newborn illnesses. 69.8% of the recently  delivered women received CBSV assessment visits and 10% newborns were referred to facility where service was not sometimes satisfactory. | **Care seeking for newborn illnesses:** Rural residence^↑^, low SES quintile^↑^  **Compliance with referrals:** Rural residence^↑^, low SES quintile^↑^ |
| Matovu et al 2014^(27)^* | Design: Cross-sectional  Duration: NA  Population: Caregivers of under 5 children who had received treatment for malaria or pneumonia  Quality: Good  Country: Uganda | CMDs; distributed artemether-lumefantrine and amoxycillin for malaria and pneumonia, respectively for children under 5 | Indirect costs and travel time for health facility visits were higher for rural caregivers. Seeking care from CMDs is more relevant and cheaper and likely to ensure more prompt management of illness. | **Direct treatment costs for seeking care from CMDs:** Rural^↓^ due to lower travel cost, travel time is longer for rural  **Opportunity costs for healthcare-seeking from CMDs:** Travel time, waiting time and total time spent to get treatment in rural location^↑^, |
| Mitra et al. 2018^(109)^ | Design: Cross-sectional  Duration: around 1 year  Population: Newborns (0-9 days old)  Quality: Good  Country: Bangladesh | CHW; Home visits, health messages on newborn and postnatal care, supplies of iron and folic acid, and delivering a clean birthing kit. | With CHW intervention, neonatal infection rate was 14.5% and risk factors included home delivery, unclean cord care, low birth weight, birth asphyxia and previous child death. Higher pregnancy order lowered the risk of infections. | **Neonatal infection risk:** Gender ^NS^, mother's and father's education ^NS^, religion^NS^, SES quintile ^NS^ |
| Miyaguchi et al. 2014^(34)^* | Design: Cross-sectional survey data  Duration: Not reported  Population: Caregivers of under 5 children with illness history  Quality: Good  Country: Nepal | FCHVs; Diagnosis and treatment of acute respiratory infection and diarrhoeal disease. | Most of the participants and 92.3% of those who had illnesses but did not seek care for childhood illnesses from FCHVs were unaware of their services. | **Health service utilisation:** Greater distance^↓^, Mother’s education ^NS^, SES quintile ^NS^, caste ^NS^ |
| Nair et al. 2017^(101)^ | Design: Cluster RCT  Duration: 18 months  Population: Pregnant women and children  Quality: Good (low risk)  Country: India | CHWs; Home visits, nutritional counselling participatory learning, promotion and identification of undernourished children | In intervention clusters, more pregnant women and children attained minimum dietary diversity, more mothers washed their hands before feeding children, fewer children were underweight, and fewer infants died. | **Children's length for age Z scores:** Gender ^NS^, high SES quintile^↑^. |
| Shah et al. 2014 ^(52)^ | Design: Cross-sectional  Duration: 10 months  Population: Pregnant and post-partum women  Quality: Good  Country: Bangladesh | Home visits, maternal & newborn health package delivery (clean delivery kit, infection prevention & messages | Reducing NMR caused by preterm would decrease it by 31% & preparedness for birth & newborn care was associated with lower risk. | **Neonatal mortality in pre-term babies:** Male babies^↑^, mother’s & father’s education ^NS^, religion ^NS^, low SES quintile^↑^ |
| Sharkey et al. 2014^(88)^* | Design: Case studies, survey data  Duration: 2 years during 2010 & 2012 (intervention) surveys  Population: stakeholders in iCCM programmes  Quality: No QA as secondary data  Country: Mozambique & Niger | CHW; iCCM integration with treatment of childhood fever, malaria, pneumonia, and diarrhoea and health promotion | Results indicated a trend of progress in diagnosis and treatment seeking behaviours from appropriate providers as a result of CHWs & iCCM integration in the communities. The equity ratio for early treatment of childhood illness increased from 0.04 to 0.83 in low SES. | **Care–seeking for childhood illnesses within 24 hours:** More distance^↓^, low SES quintile^↑^ |
| Shaw et al. 2015^(30)^* | Design: Cross-sectional  Duration: 3 years  Population: Caregivers of children under 5  Quality: Good  Country: Ethiopia | HEW; iCCM integration, basic health services, childhood disease prevention, treatment control and referral. | High utilisation of HEW services for childhood illnesses (>90%) was found in both iCCM upgraded and non-upgraded zones; even higher in iCCM zones. | **Utilisation for HEW driven care for childhood fever, diarrhoea, pneumonia:** Low distance^↑^, gender^NS^, religion ^NS^, low mother’s education^↑^ |
| Soremekun *et al.*  2018 ^(89)^* | Design: Cross-sectional  Duration: Not reported; VHT in place since 2011  Population: Caregivers of children under 5  Quality: Fair  Country: Uganda | Village Health Team (VHT); iCCM training and implementation for treatment of malaria, diarrhoea and pneumonia in children under 5 years of age | 20% of children with illness were first taken to a CHW and CHWs were more likely to provide the appropriate treatment than other providers and CHW can increase its coverage  from 47% up to 64%. | **First care sought for child illnesses from CHWs:** Mother’s education^NS^ and occupation^NS^, Muslims^↓^, low SES^↑^ |
| Yirgu et al. 2017^(123)^ | Design: Population based cohort Duration: MaNHEP working Since 2010  Population: Pregnant women  Quality: Good  Country: Ethiopia | HEW and CHWs in MaNHEP; health education for bringing positive behavioural change | The neonatal mortality rate in the intervention area was 18.6 per 1000 live births which was 3 times lower than national average. | **Neonatal mortality:** Gender ^NS^, occupation ^NS^, high mother’s and father’s education^↓^ |

**Table A4-3. Studies of Maternal and Child-Focused CHW Interventions**

| Authors & Year | Characteristics | CHW Intervention | Summary results | Outcome by Equity stratifier |
| --- | --- | --- | --- | --- |
| Adams et al. 2015^(103)^* | Design: Cross-sectional  Duration: 5 years  Population: Women who gave birth recently in poor urban area  Country: Bangladesh  Quality: Good | Shasthya Sebikas and Kormis, Urban Birth Attendants and Manoshi midwives; Home visits, health education, follow-up, referral and provided skilled birth attendance. | Manoshi intervention and their presence in participant’s support network resulted in greater use of SBA and PNC, and feeding colostrum to their newborn. | **Use of SBA:** Mother’s education^NS^, SES quintile^NS^, Social capital^↑^  **Colostrum feeding:** Mother’s education^NS^, high SES quintile^↑^, Social capital^↑^  **PNC:** Mother’s education^NS^, high SES quintile^↑^, Social capital^↑^ |
| Angeles et al. 2019^(54)^* | Design: Quasi-experimental surveys  Duration: 2006-2013  Population: slum and non-slum residents in urban  Quality: Good  Country: Bangladesh | CHW; home visits, CHW conducted ANC, PNC, reproductive health services and facilitate use of SBA and also provide it. | Intervention had reduced the gaps in utilisation of SBA and family planning between slum and non-slum but no significant effect on childhood stunting. | **Use of SBA:** Slum residence^↓^, greater distance ^↓^, mothers’ education^↑^, high possession of assets^↑^  **Use of family planning:** Slum residence^↓^, greater distance ^↓^, mother’s education^↑^, low possession of assets^↑^.  **Stunting:** Slum residence^↑^, greater distance^↑^, mother’s education^↓^, low possession of assets ^↑^. |
| Avery et al. 2017^(93)^ | Design: Non RCT, quasi-experimental  Duration: 3 years  Population: pregnant/post-partum women, rural  Quality: Good  Country: Kenya | CHVs; implementation of a new monitoring and tracking tool to identify and track pregnant women and under 5 children | Intervention improved  ≥4 ANC visits, facility delivery, PNC, early and exclusive breastfeeding practices | **ANC:** Asset tertile^↑^_,_ mother’s education^↑^  **Facility delivery:** Mother’s education^↑^ Asset tertile^NS^  **Postnatal care:** Asset tertile^NS^, mother’s education^NS^  **Exclusive Breastfeeding:** Asset tertile^↑^, mother’s education^NS^  **Early Breastfeeding:** Asset tertile^NS^, mother’s education^NS^  **Stunting:** Slum residence^↑^, greater distance^↑^, high mother’s education^↓^, low possession of assets ^↑^. |
| Balakrishnan et al. 2016^(156)^* | Design: Cross-sectional  Duration: Data of 3 years  Population: women and children, rural  Quality: No QA, secondary data  Country: India | agents de santé communautaire (ASC); mobile tracking for Continuum of Care Services (CCS) in pregnancy and MNCH outcomes till the child was 6 years old | Intervention blocks had higher coverage of all the eight indicators for MNCH services | **ANC, facility delivery, PNC, early breastfeeding initiation, antetetanus vaccination, iron folate supplementation:** Caste^NS^ |
| Boone et al. 2017^(53)^* | Design: Cluster RCT  Duration: around 3 years  Population: Women <50 with children  Quality: Good (low risk)  Country: India | VHWs, midwives; culturally appropriate health promotion and education, referral, midwives delivered ANC, PNC, SBA, infection prevention, subsidised rate of care | Intervention improved health knowledge and service utilisation, and reduced neonatal mortality significantly, but not maternal mortality | **Neonatal mortality:** Distance^NS^, tribes^NS^ |
| Chudasama et al. 2014^(25)^ | Design: Cross-sectional  Duration: No info  Population: AWWs, mothers, children and adolescents  Quality: Fair  Country: India | AWW (Angawadi worker); health promotion, check-up and referral services along with supplementation for children, pregnant & lactating mothers, immunisation | Supplementation coverage was reported in 48.3% in children and  immunisation for children recorded in only 10% Anganwadi centres.  Regular health checkup was done in 30%  and referral of sick children  was done from only 8.3% centres. | **Coverage of supplementary nutrition:** Residence^NS^.  **Acceptance of supplementary nutrition:** Rural ^↑^  **Adolescent health services receipt:** Rural ^↑^ |
| Ekirapa-Karacho et al. 2017^(94)^ | Design: Quasi-experimental  Duration: Two years  Population: Rural women and newborns  Quality: Good  Country: Uganda | CHWs; community mobilisation and health promotion, training of health workers | Intervention led improvements in ANC attendance, facility delivery and newborn care | **ANC:** Mother’s education and occupation^NS^, wealth index^↑^, minority religion^↓^  **Facility delivery:** Mother’s education and occupation^NS^, wealth index^NS^, religion^NS^  **Newborn care practices:** Mother’s education^↑^ and occupation^NS^, minority religion^↑^ |
| Geldsetzer *et al.*  2017b^(22)^ | Design: Cross-sectional  Duration: Not reported Population: individuals and households; rural  Quality: Good  Country: Eswatini | CHW, Rural Health Motivators (RHM); Home visits, education, referral, follow up and referral, hygiene and sanitation, growth monitoring & promotion | Only 44.5% households  reported that they had ever been visited by a CHW. | **CHW visit coverage:** Rural^↑^, Men^↑^, high education^↓^, occupation ^NS^, low household wealth^↑^ |
| Luckow et al. 2017^(119)^* | Design: Non-RCT, Quasi-experimental  Duration: 3 years  Population: Women aged 18–49 years  Quality: Good  Country: Liberia | CHW; Free health services with iCCM scale up, home visits, treatment and referral, maternal and newborn care and ANC education, children’s growth monitoring. | The proportion of children receiving health care for illnesses from formal providers and facility delivery significantly increased **which was mostly provided by CHWs.** | **Facility delivery:** Agricultural communities^↑^  **Formal care for diarrhoea:** Agricultural communities^↑^  **Formal care for fever:** Agricultural communities^↑^  **Formal care for acute respiratory illness:** Agricultural communities vs gold mining ^NS^ |
| Kosec et al. 2015^(81)^ | Design: Cross-sectional  Duration: Not reported  Population: Community members  Quality: Good  Country: India | FLWs, ASHAs, AWWs; Home visits, nutrition education, supplementation for children, pregnant & lactating women, promote immunisation and family planning, follow-up & referral | Monetary immunisation incentives for  AWWs and that for ASHAs for institutional delivery were predictors of HH receipt of immunisation services and pregnancy care. | **Complete receipt of immunisation:** Household head’s education^↑^, same caste as ASHA^↑^, same caste as AWW^↓^, SES quintile ^NS^  **Coverage of food supplement:** Household head’s education^NS^, same caste as AWW ^NS^, lower SES quintile^↑^ |
| Vilms et al. 2017^(24)^* | Design: Cross-sectional  Duration: Not reported  Population: Infants and caregivers  Quality: Good  Country: India | FLW, ASHAs's auxiliary nurse midwives, AWWs; Home visits, education and promotion on newborn care and FLW conducted PNCs | Girl neonates had lower rates of reported illness & lower odds of receiving care & PNC checkup which was more profound in poorer families. Gender differences in immunisation  and FLW visits were not found. | **Prevalence of sickness in neonates:** Female neonates^↓^, residence ^NS^, adolescent and Muslim mothers^↑^, mother’s education ^NS^, wealth index ^NS^  **Vaccination:** Residence ^NS^, gender^NS^, Muslim^↓^, mother’s education ^NS^, high wealth index^↑^  **FLW advised seeking care for neonatal illnesses:** Rural residence^↑^, gender^NS^, marginalised caste^↑^, Muslims^↓^, high wealth index^↑^  **Receipt of care for neonatal illness:** Residence^NS^, female neonates^↓^, religion ^NS^, high wealth index^↑^  **Facility check-up at one month:** Residence^NS^, female neonates^↓^, mother’s education ^↑^, castes ^NS^, religion ^NS^, wealth index ^NS^  **PNC visit by FLW at one month:** Rural residence^↑^, mother’s education ^NS^, castes ^NS^, religion ^NS^, wealth index ^NS^ |
| Yaya et al. 2015^(32)^ | Design: Cross-sectional  Duration: NA  Population: People in 75 villages  Quality: Good  Country: Ethiopia | HEWs; health promotion, child vaccinations, family planning, antenatal care, and assisting normal deliveries, register birth and deaths. | The validated data of HEW conducted survey showed that it was possible to obtain a high-coverage birth registration using HEWs and measure MMR in a community with an existing functional system of CHWs. | **MMR:** Distance ^NS^, Mother’s education ^NS^, father’s education^↓^, no driveable road access^↑^  **Completeness of registration of birth:** Greater distance^↓^ |
| Yitayal *et al.* 2014^(83)^ | Design: Cross-sectional  Duration: 4 years  Population: Mothers  Quality: Good  Country: Ethiopia | HEW; Home visits, primary health care services and family training on basic hygiene and environmental sanitation, family health care, and disease prevention and control | The proportion of community utilisation of health extension service was 39%. Occupation, knowledge about HEW, community participation were predictors of utilisation. | **HEW service utilisation** (includes both maternal and other services): Mother’s occupation^NS^, high family income^↑^ |

**Table A4-4. Studies of Adolescent, Youths, and Adults-Focused CHW Interventions**

| Authors & Year | Characteristics | CHW Intervention | Summary results | Outcome by Equity stratifier |
| --- | --- | --- | --- | --- |
| Bello et al. 2017^(141)^ | Design: Cluster RCT  Duration: approximately 2 years.  Population: Rural  Quality: Good (low risk)  Country: Malawi | Trained unpaid informal providers (IPs); home visits, education, promotion, TB and HIV disease recognition, sputum specimen collection, appropriate referrals for testing and treatment, and raising community  awareness. | The engagement of unpaid IPs increased TB and HIV testing rates but treatment initiation rates only improved for HIV patients (ART). | **HIV tests:** Greater distance^↓^, male^↓^  **HIV treatment initiation:** Greater distance^↓^, Gender^NS^  **TB tests:** Greater distance^↓^, female^↓^  **TB treatment initiation:**  Greater distance^↓^, female^↑^ |
| Borg et al. 2018^(41)^* | Design: Cluster RCT  Duration: Around 6 weeks  Population: Adolescents  Quality: Fair (Moderate risk)  Country: Bangladesh | Accredited Social Health Activists (ASHAs); home visits, testing (assessments of hearing score), follow-up & referral | The community-based approach performed as good as the centre-based approach on five out of  seven International Outcome Inventory for  Hearing Aids (IOI-HA) outcome measures. | Hearing aid use: Rural location^↓^, Gender^NS^  Improved activity: Rural location^↑^, Gender^NS^  Activity limitation: Rural location^↓^, Gender^NS^  Satisfaction: Rural location^↑^, Gender^NS^  Participation restriction: Location^NS^, Gender^NS^  Impact on others: : Location^NS^, Gender^NS^  Quality of life: : Location^NS^, Gender^NS^ |
| Chami et al. 2017^(35)^ | Design: Cross-sectional  Duration: -  Population: Survey at individuals and households level  Quality: Good  Country: Uganda | Community Medicine distributors (CMDs); mass drug administration (MDA) for hookworm and filariasis infection | CMDs were more likely to offer and accurately  administer the drugs to influential individuals, and households that were closer to the network. Compliance was associated with trusting CMDs for health advice. | **Coverage:** More distance^↓^, gender^NS^, high social status^↑^, high education^↓^, Muslim household head^↓^, Majority tribe ^NS^, no latrine^↓^, home owner ^NS^, home quality ^NS^, fishmonger^NS^ or other occupation^NS^  **Compliance:** Gender^NS^, social status^NS^, high education^↓^, Muslim household head ^NS^, Majority tribe ^↑^, no latrine^NS^, home owner ^NS^, home quality^NS^, fishmonger as occupation^NS^, other occupation^↑^ |
| Coker et al. 2015^(137)^ | Design: Cluster RCT  Duration: 9 months intervention, 18 months follow-up  Population: Muslim HIV-infected adults  Quality: Poor (high risk)  Country: Nigeria | Peer-educators (PE); Group 1: standard care (SC), Group 2: SC+daily reminders and follow-up+ adherence support by PE, Group 3: Group 1+2+ home visits by PE | Antiretrovirals adherence will improve significantly regardless of whether HIV-infected adults received peer-education-based medication or not. | **Viral load suppression:** Gender^NS^, education^NS^, marital status^NS^.  **Retention in follow-up:** Female^↑^ |
| Cros et al. 2019^(82)^ | Design: Quasi-experimental  Duration: 2 years (2012-13)  Population: Household survey  Quality: Fair  Country: Haiti | CHWs; not detailed | The CHW service resulted in a pro-poor service utilisation (inequality index of  -0.22) and households seeking  care from CHWs were 3.5 times less likely to incur catastrophic health expenditure (measure of financial risk). | **Utilisation of CHW outpatient services for CDs and NCDs**: Low SES^↑^  **CHE:** Place of residence^NS^ |
| Fatti et al. 2018^(42)^ | Design: Retrospective cohort  Duration: 6 years (2004-10)  Population: Adolescents and youth aged 10 to 24 years who are on antiretrovirals (ART)  Quality: Good  Country: South Africa | Community based support (CBS) workers; home visits, ART-related education, psychosocial support, screening for infections and support to access government grants. | Participants who received CBS had reduced mortality, adjusted hazard ratio and lower losses to follow-up rates and lower viral suppression failure than those without CBS. | **Loss to follow-up & mortality:** Place of residence^NS^, Gender ^NS^ |
| Geldsetzer et al. 2017^(118)^ | Design: Cross-sectional  Duration: Not reported  Population: Individuals and households; rural  Quality: Good  Country: Eswatini | CHW, Rural Health Motivators (RHM); home visits, providing information on hygiene, sanitation, communicable diseases, referral of ill household members, attending medical emergencies and follow up, promoting adult literacy | 49% of participants in the survey stated that they distrust the national CHW cadre with  confidential health information (e.g. HIV). | **Trust RHMs with confidential health information:** Low education^↑^, poorest and least educated households^↑^, being employed^↑^ |
| Getnet et al. 2017^(26)^ | Design: Cross-sectional  Duration: Existing HEW programme from 2003.  Population: Somali pastoralists  Quality: Good  Country: Ethiopia | HEWs; preventive health actions and increased health awareness, TB identification and referral. | The HEW’s contribution in identifying and referring presumptive TB cases is limited  in Ethiopian Somali pastoralist region. | **Pulmonary TB referral:** Rural^↑^, time to travel to health post^NS^, patient’s gender ^NS^, education ^NS^, family income^NS^ |
| Hailemariam et al. 2019^(40)^ | Design: Cross-sectional  Duration: Half a year  Population: People with severe mental disorders  Quality: Good  Country: Ethiopia | HEWs; Identify and refer people with probable SMD | Integrating mental healthcare into primary care through HEW resulted in high levels of coverage (81%)  gender and socio-economic status. | **Coverage in mental heath access:** Gender ^NS^, SES ^NS^, education^NS^, disability ^NS^ (physical or sensory impairment) **as Non-access:** Higher disability score^↓^ |
| Linn et al. 2018^(140)^* | Design: Cross-sectional  Duration: -  Population: Survey  Quality: Good  Country: Myanmar | Village health volunteers (VHV); Diagnosis and treatment for malaria and link with health facilities | VHVs provided screening services to a higher proportion of children aged over 5 years (21.8% vs 17.3%) and females (43.7% vs 41.8%) compared to BHS. The proportion of patients receiving treatment was higher from VHVs(96.6% vs 94.9%) too. This was true for early treatment (within 24 hours) but rates of complete treatment was lower among VHV treated patients (80.9% vs 88.2%). | **Coverage of diagnosis of malaria:** Female^↑^, pregnant female^↓^  **Malaria treatment receipt:** Gender^NS^ |
| Lusli et al 2016^(142)^ | Design: Cluster RCT (Mixed methods)  Duration: 2 years (2012-13)  Population: People affected by leprosy  Quality: Poor/High risk  Country: Indonesia | Lay and peer counsellors (tained on counselling); five counselling sessions (two individual, one family, and two group) | Counselling can be effective in reducing stigma and have different impacts on stigma and participation restrictions on men and women | **Leprosy-related stigma reduction:** Female^↑^ |
| Matsumoto-Takahashi et al. 2014^(198)^* | Design: Cross-sectional  Duration: active since 1990  Population: Previously diagnosed malaria-positive cases by the microscopists,  Quality: Poor  Country: Philippines | Microscopists;  diagnose malaria, and prescribe anti-malarials, community awareness-raising activities | Apart from providing early diagnosis and treatment, microsco-  pists played a significant role in self-implemented preventive measures against malaria through their awareness-raising activities | **Self-implemented preventive measures against malaria:** Tagalog (predominant) ethnicity^↑^ |
| Naidoo et al. 2018^(36)^ | Design: Cross-sectional  Duration: 3 years  Population: Adults in households to report  Quality: Good  Country: S. Africa | CHWs; register households, home visits and education to prevent HIV infection, identifying at risk population to test for HIV, referring HIV-infected people for antiretroviral therapy  (ART), providing adherence support and follow-up | Greater visit coverage and frequency reported in Greater Giyani was possibly due to better organisational structures.  96% and 67% of the participants reported having discussed any disease and particularly HIV with the CHW, respectively. | **Coverage and frequency of CHW visits**: Location Giani^↑^, distance to the health facility^NS^, source of income^NS^, gender^NS^  **Implementation fidelity (measurement of content):** Females^↑^ (more likely to know HIV status than men) |
| Russell et al. 2015^(124)^ | Design: Cross-sectional  Duration: Few months  Population: Households (survey)  Quality: Fair  Country: Nigeria | CHPs; monthly home visits for social behaviour change intervention (information, encouragement and logistics support), mobilisation of community to increase the correct and consistent use of insecticide treated bednets and workshops. | CHW home visit increased the odds of bednet use (OR =  17.11; 95% CI 4.45–65.79) and it is significantly influenced by social support from friends and families. | **Use of bednet among households that own at least one:** Occupation^NS^, SES^NS^,  social support^↑^, low education^↑^ |
| Shidaye et al. 2017^(136)^* | Design: Quasi experimental  Duration: 1.5 years  Population: Individuals with depression; rural  Quality: Fair  Country: India | CHWs; group meetings and household visits to increase awareness about mental disorders and to inform people about services available, identify cases of depression, provide mental health “first aid”, and refer individuals with greater needs to the next tier of workers. | Contact coverage, defined as  individuals with depression who sought treatment for symptoms  of depression, was six-times higher after the intervention started and improvements in mental health literacy noted. | **Contact coverage for current depression:** SES^NS^, gender^NS^, being married ^NS^, scheduled caste/tribe^↓^, religion^NS^, education^NS^ |
| Teklehaimanot et al. 2016^(33)^ | Design: Cross-Sectional  Duration: -  Population: Men aged 15-59 and women aged 15-49; rural  Quality: Good  Country: Ethiopia | CHWs and HEWs; Voluntary counseling and testing (VCT) for HIV/AIDS | VCT uptake was higher among youth and better-educated individuals. Women from households with greater economic wealth were more likely to be tested. | **VCT uptake in rural women:** Greater distance^↓^, farming occupation of household head^↓^, high SES^↑^, male-headed household^↓^, Muslim^↑^, high education^↑^  **VCT uptake in rural men:** Distance^NS^, farming occupation of household head^↓^, SES (wealth index)^NS^, male-headed household ^NS^, Muslim^↑^, high education^↑^ |

**Footnotes for Table A4-1 to A4-4:**

*CHW-directed health service promotion & delivery of ANC, PNC and/or SBA service and/or treatment. Distance denotes to ‘Distance to Health Facility’. Educational attainment or employment is compared with no education or not employed. ^↑^ denotes ‘increases or high’, ^↓^ denotes ’reduces or lower’, ^NS^ denotes ‘not significant’.

Terms used: Community Health Volunteers (CHV), Maternal and Neonatal Health (MNH), Antenatal and Postnatal care (ANC, PNC), Skilled Birth Attendance (SBA), Village Health Volunteer and Workers (VHV, VHW), TBA (Traditional Birth Attendants), Maternal, Neonatal and Child Health (MNCH), Reproductive, maternal and neonatal health (RMNH), Continuum of Care (CoC), Accredited Social Health Activists (ASHAs), Anganwadi workers (AWWs), Frontline Workers (FLWs), Integrated Community Case Management (iCCM), National Rural Health Mission (NRHM), Micronutrient (MNP), Neonatal mortality Rate (NMR) and Maternal Mortality Rate (MMR), Maternal and Newborn Health in Ethiopia Partnership (MaNHEP), Community Medicine Distributors: CMDs, Community Health Promoters: CHPs
