# Supplementary material for: Community health workers and health equity in low- and middle-income countries: systematic review and recommendations for policy and practice
Source: Int J Equity Health. 2022 Apr 11;21:49. doi: 10.1186/s12939-021-01615-y (PMC8996551; doi:10.1186/s12939-021-01615-y)
Supplement: Supplementary file 5 — Additional file 5. Funnel Plots to Investigate Publication Bias: Presents funnel plots that were used to investigate publication bias. [file 12939_2021_1615_MOESM5_ESM.docx]

**Additional File 5: Funnel Plots to Investigate Publication Bias**

**Figure A1: Assessing publications bias for studies comparing facility delivery and distance of households from health facility**

**
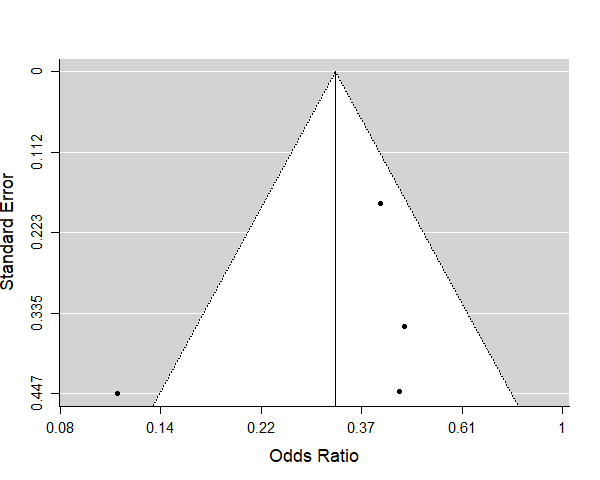
**

**Figure A2: Funnel plot for association for between high and low SES and antenatal care**

**
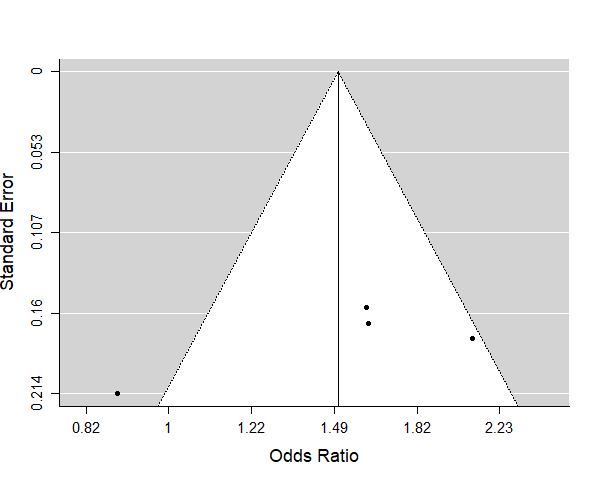
**

**Figure A3: Funnel plot for the association between high and low SES and exclusive breastfeeding**

**
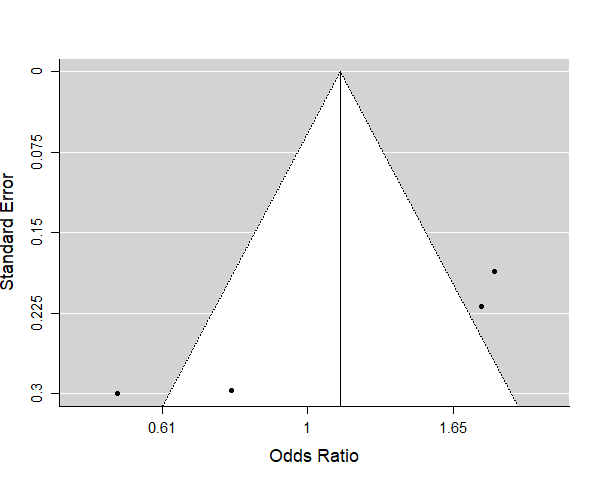
**

**Figure A4: Funnel plot for association between SES and institutional delivery
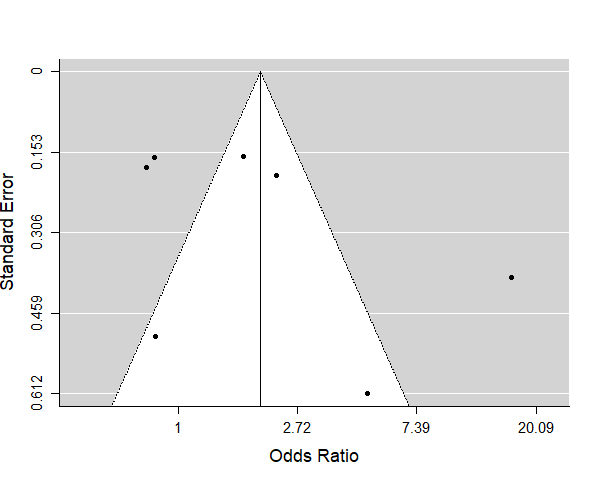
**

**Figure A5: Funnel plot for the association between SES and postnatal care**

**
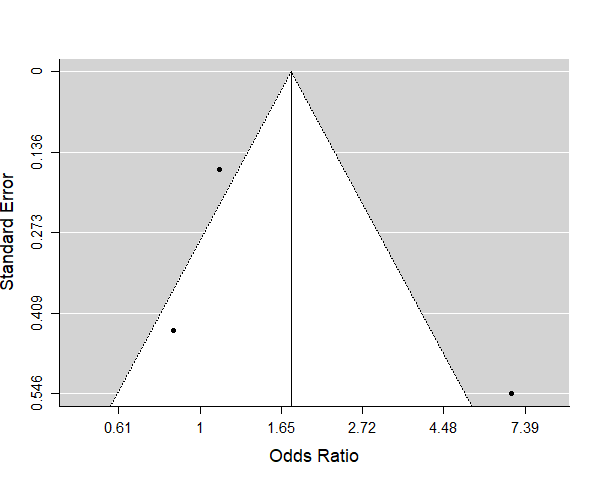
**

**Figure A6: Funnel plot for the association between education level and antenatal care (ANC)
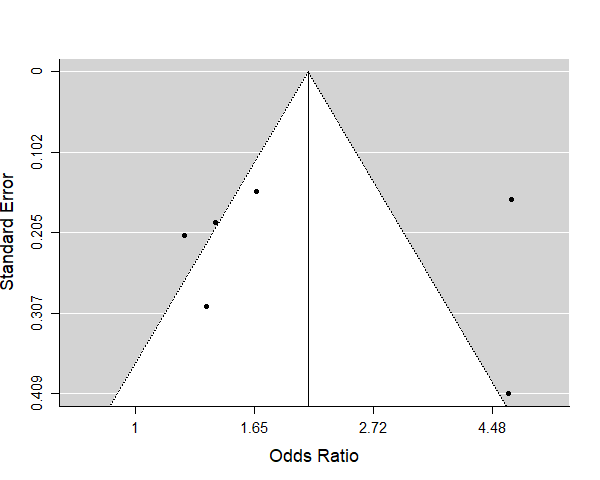
**

**Figure A7: Funnel plot for association between level of education and exclusive breastfeeding
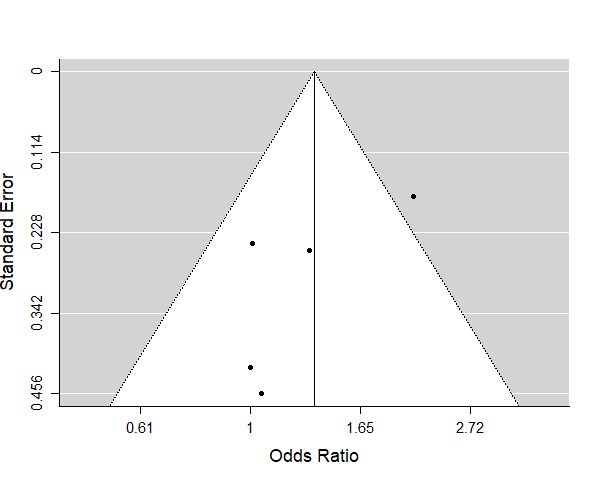
**

**Figure A8: Funnel plot for association between level of education and institutional delivery.**

**
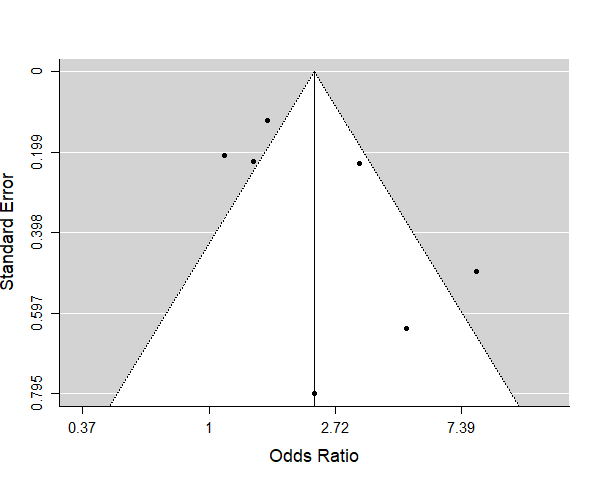
**

**Figure A9: Funnel plot for association between level of education and postnatal care**

**
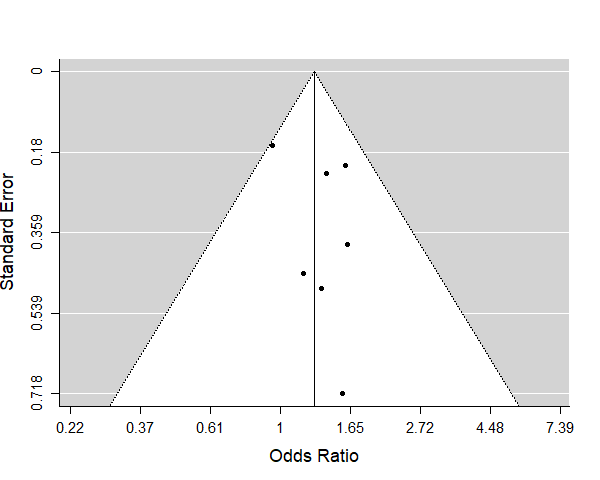
**

**Figure A10: Funnel plot for association between level of education and skilled birth care**

**
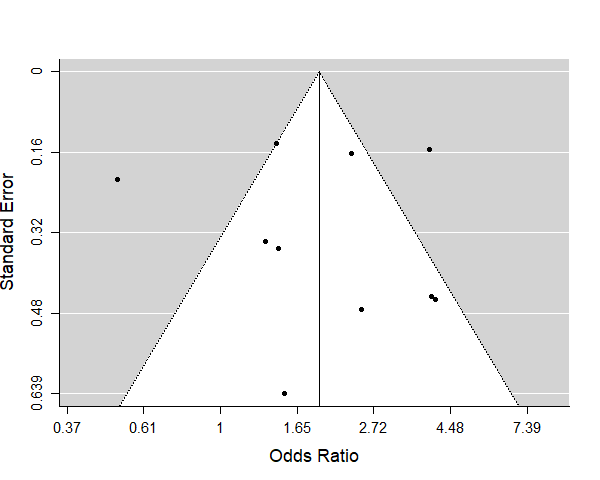
**
